# Supplementary material for: A chemical catalyst enabling histone acylation with endogenous acyl-CoA
Source: Nat Commun. 2023 Sep 22;14:5790. doi: 10.1038/s41467-023-41426-z (PMC10517024; doi:10.1038/s41467-023-41426-z)

Supplementary information

A chemical catalyst enabling histone acylation with endogenous Acyl-CoA

Misuzu Habazaki[a], Shinsuke Mizumoto[a], Hidetoshi Kajino[a], Tomoya Kujirai[b], Hitoshi Kurumizaka[b], Shigehiro A. Kawashima[a*], Kenzo Yamatsugu[a*], Motomu Kanai[a*].

[a]Graduate School of Pharmaceutical Sciences, The University of Tokyo 7-3-1 Hongo, Bunkyo-ku, Tokyo, JAPAN 113-0033

[b]Institute for Quantitative Biosciences, The University of Tokyo, 1-1-1 Yayoi, Bunkyo-ku, Tokyo, 113-0032, Japan

Correspondence to

Motomu Kanai (kanai@mol.f.u-tokyo.ac.jp)

Kenzo Yamatsugu (yamatsugu@mol.f.u-tokyo.ac.jp)

Shigehiro A. Kawashima (skawashima@mol.f.u-tokyo.ac.jp)

Contents

Supplementary Figures **S1**

Supplementary Methods **S19**

General S19

Materials S19

LC/MS S19

Analytical HPLC S19

Preparative HPLC S20

Synthetic procedures S21

Chemical experiments S43

Biochemical experiments S43

Supplementary Tables **S45**

Supplementary References **S50**

Supplementary Spectral Data **S51**

Supplementary Figures

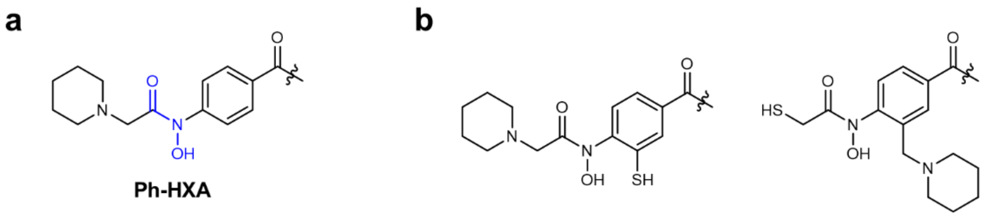

Supplementary Fig. 1 Strategy of the initial catalyst design

a Chemical structure of Ph-HXA, which was previously reported as a superior nucleophilic catalyst to DMAP under physiological conditions[1]. **b** Chemical structures of initially targeted catalysts. These hydroxamic acid catalysts with both a piperidine moiety and a thiol group could not be synthesized due to instability of synthetic intermediates.

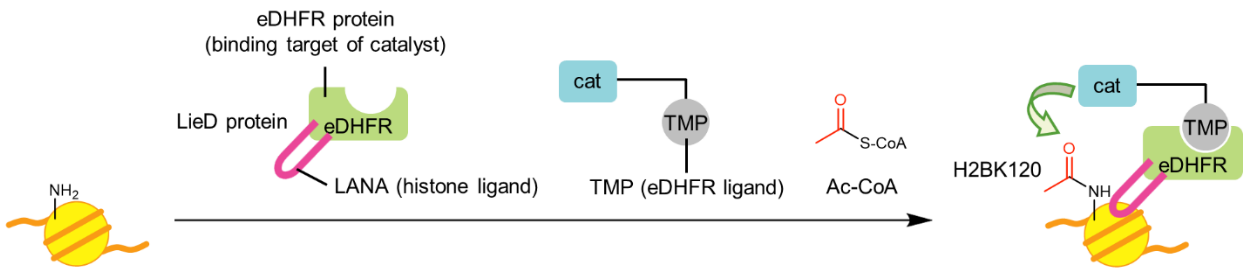

Supplementary Fig. 2 Regioselective acetylation of histone H2BK120

Schematic illustration of LieD system for histone H2BK120-selective acetylation[2]. LieD is LANA peptide-inserted eDHFR protein, which binds to the histone acidic patch through the LANA ligand. The catalyst conjugated with eDHFR ligand TMP can be targeted to histones through eDHFR-TMP interaction.

a

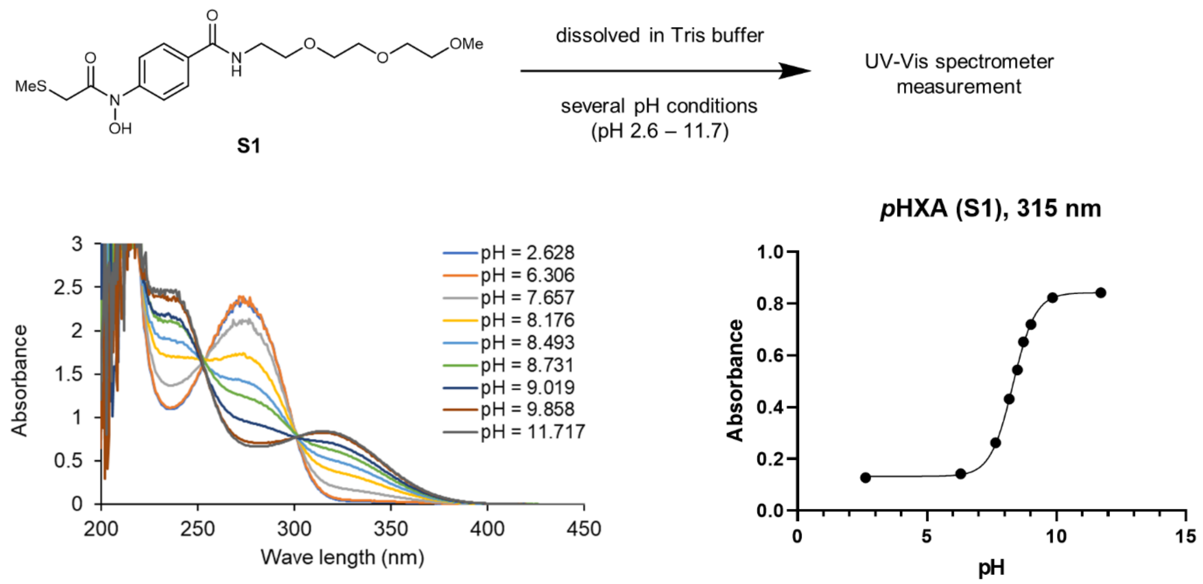

$$f(\mathrm{pH}) = a + \frac{b}{1 + 10^{(\mathrm{pH}-\mathrm{p}Ka)}}$$

pK_a = 8.323

b

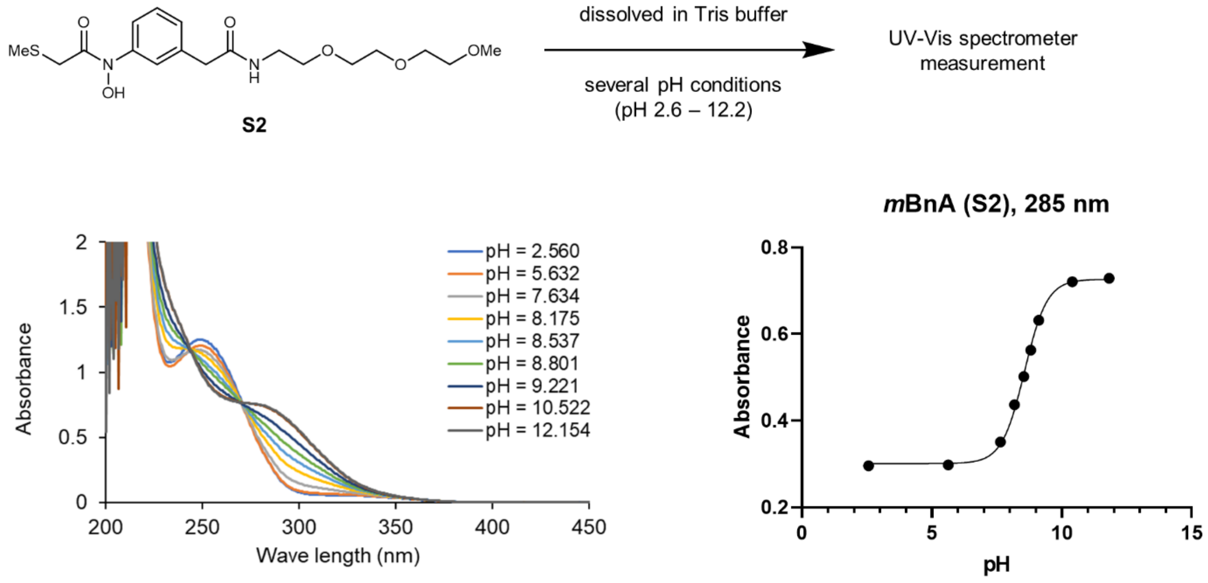

$$f(\mathrm{pH}) = a + \frac{b}{1 + 10^{(\mathrm{pH}-\mathrm{p}Ka)}}$$

pK_a = 8.564

Supplementary Fig. 3 pK_a determination of the hydroxamic acid catalysts

a-**b** Absorption spectra of catalyst *p*HXA (**a**) and *m*BnA (**b**) were measured with UV-Vis spectrophotometer under variable pH conditions (left) and the absorbance at the indicated wave length are plotted (right). To eliminate the influence of acidic protons other than hydroxamic acid, *S*-methylated derivatives (**S1**, **S2**, 500 μM) were used. An equation used for the fitting is shown[1]. Source data are provided as a Source Data file.

*p*HXA-TMP(gly$_2$)

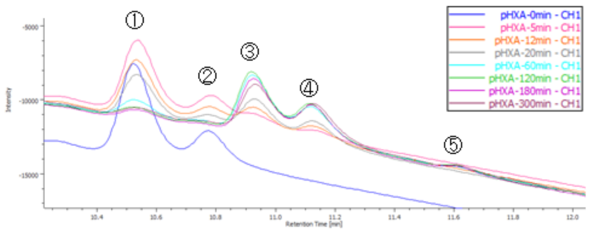

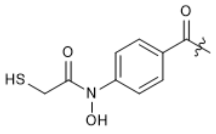

① "SH" species of hydroxamic acid

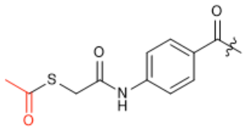

④ "SAc" intermediate of amide

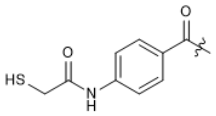

② "SH" species of amide

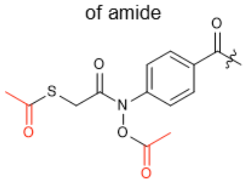

⑤ "SAc, OAc" intermediate of hydroxamic acid

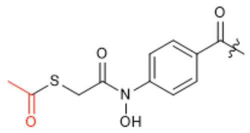

③ "SAc" intermediate of hydroxamic acid

*m*HXA-TMP(gly$_2$)

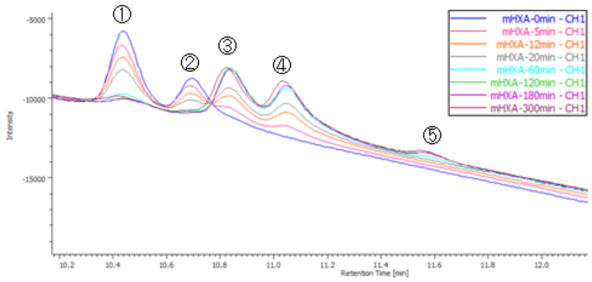

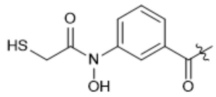

① "SH" species of hydroxamic acid

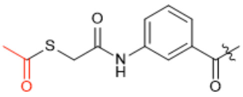

④ "SAc" intermediate of amide

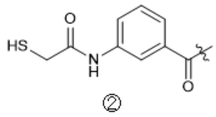

② "SH" species of amide

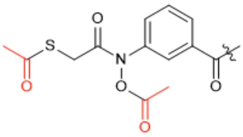

⑤ "SAc, OAc" intermediate of hydroxamic acid

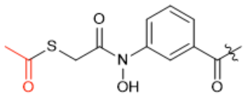

③ "SAc" intermediate of hydroxamic acid

*m*BnA-TMP(gly$_2$)

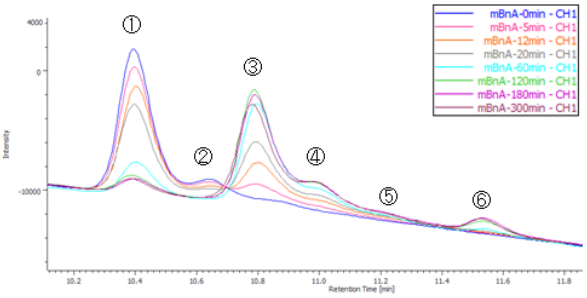

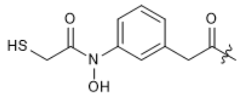

① "SH" species of hydroxamic acid

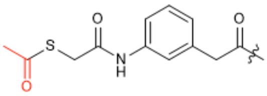

④ "SAc" intermediate of amide

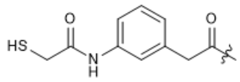

② "SH" species of amide

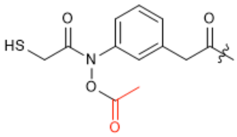

⑤ "OAc" intermediate of hydroxamic acid

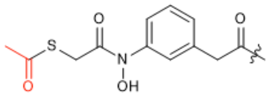

③ "SAc" intermediate of hydroxamic acid

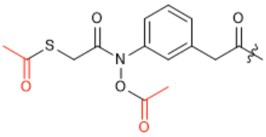

⑥ "SAc, OAc" intermediate of hydroxamic acid

Supplementary Fig. 4 *S*,*S*- and *S*,*O*-acetyl transfer reaction of the catalysts

HPLC traces of the *S*,*S*- and *S*,*O*-acetyl transfer reaction. Each catalyst (25 μM) was reacted with Ac-CoA (5 mM) in the presence of TCEP (200 μM) for the indicated time. The structures of the intermediate corresponding to each peak were identified by LC/MS. Representative data from two independent experiments are shown. Amide species were generated presumably due to the reduction by TCEP. For the yield calculations of “SAc” and “OAc” intermediates, see the Methods section.

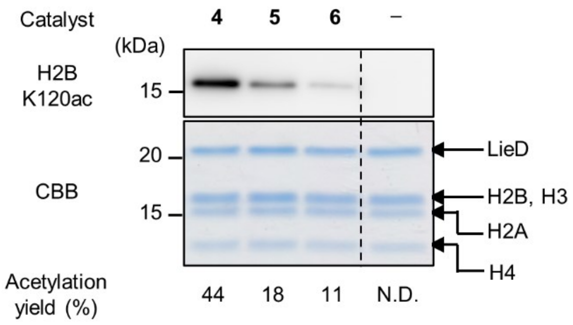

Supplementary Fig. 5 H2BK120-acetylation abilities of the negative control catalysts in test tube reaction

Recombinant nucleosome (0.35 μM) was reacted with the indicated catalysts (5 μM, *m*BnA-TMP(gly$_1$) **4**, *m*BnA-OMe-TMP(gly$_1$) **5**, and *m*BnA-SMe-TMP(gly$_1$) **6**) in the presence of LieD-protein ligand (2 μM), Ac-CoA (1 mM), and TCEP (200 μM) at 37 °C for 5 h, and its acetylation was detected with anti-H2BK120ac antibody by western blot analysis. Total proteins were visualized by CBB staining. Dividing lines have been used to indicate where noncontiguous sections of a gel have been aligned for ease of comparison. Representative data from two independent experiments are shown. The acetylation yields at H2BK120 were quantified with LC–MS/MS analysis. The mean chemical yields of two independent experiments are shown. “N.D.” denotes “not detected”. Source data are provided as a Source Data file.

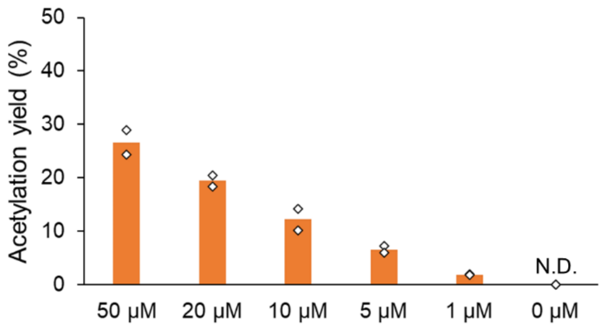

Supplementary Fig. 6 Working concentration range of Ac-CoA in H2BK120 acetylation by *m*BnA-TMP(gly$_1$) catalyst (4) in test-tube reactions

Recombinant nucleosome (0.35 μM) was reacted with *m*BnA-TMP(gly$_1$) **4** (5 μM) in the presence of LieD (2 μM), the indicated concentration of Ac-CoA, and TCEP (200 μM) at 37 °C for 10 h. The acetylation yields at H2BK120 were quantified with LC–MS/MS analysis. The mean chemical yields of the two independent experiments are shown. The overlaid dot plots present the individual data points of each experiment. "N.D." denotes "not detected". Source data are provided as a Source Data file.

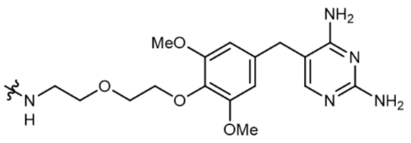

gly$_2$ linker-TMP

Compound	Structure
DSH-TMP(gly$_2$) (**S3**)	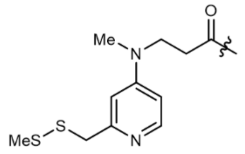
Ph-HXA-TMP(gly$_2$) (**S4**)	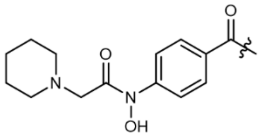
YZ-TMP(gly$_2$) (**S5**)	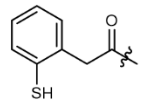

Supplementary Fig. 7 Chemical structures of catalysts with previously developed catalytic centers for acetylation of H2BK120

Previously reported lysine acetylation catalyst centers DSH (**Fig. 2a**)[3] and Ph-HXA (**Supplementary Fig. 1a**)[1], and YZ[4] were conjugated to the TMP ligand.

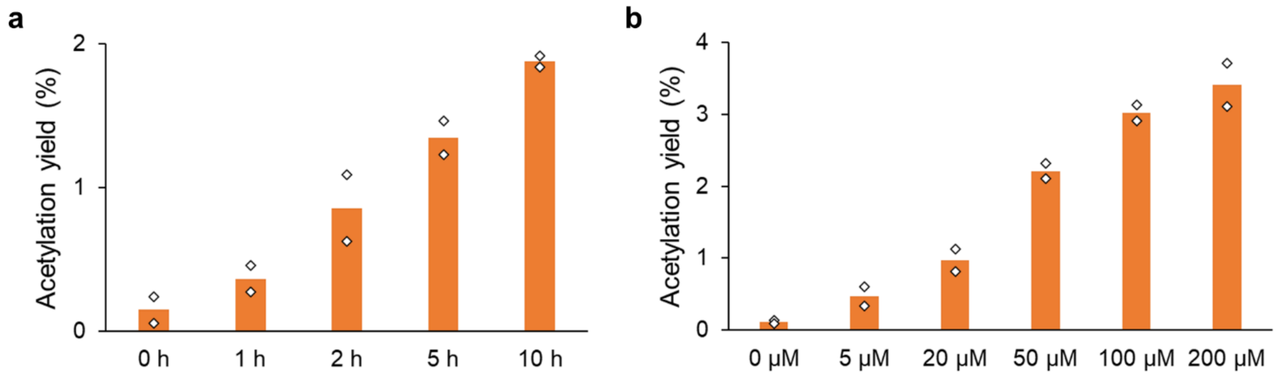

Supplementary Fig. 8 Reaction time- and catalyst concentration-dependence of histone H2BK120 acetylation by *m*BnA-TMP(gly$_1$) catalyst (4) in cells

a LieD-transfected HEK293T cells were treated with **4** (100 μM) for the indicated time. **b** LieD-transfected HEK293T cells were treated with the indicated concentration of **4** for 10 h. Histone proteins were acid-extracted, and H2BK120ac yields were quantified with LC–MS/MS analysis. The mean chemical yields of the two independent experiments are shown. The overlaid dot plots present the individual data points of each experiment. Source data are provided as a Source Data file.

a b c
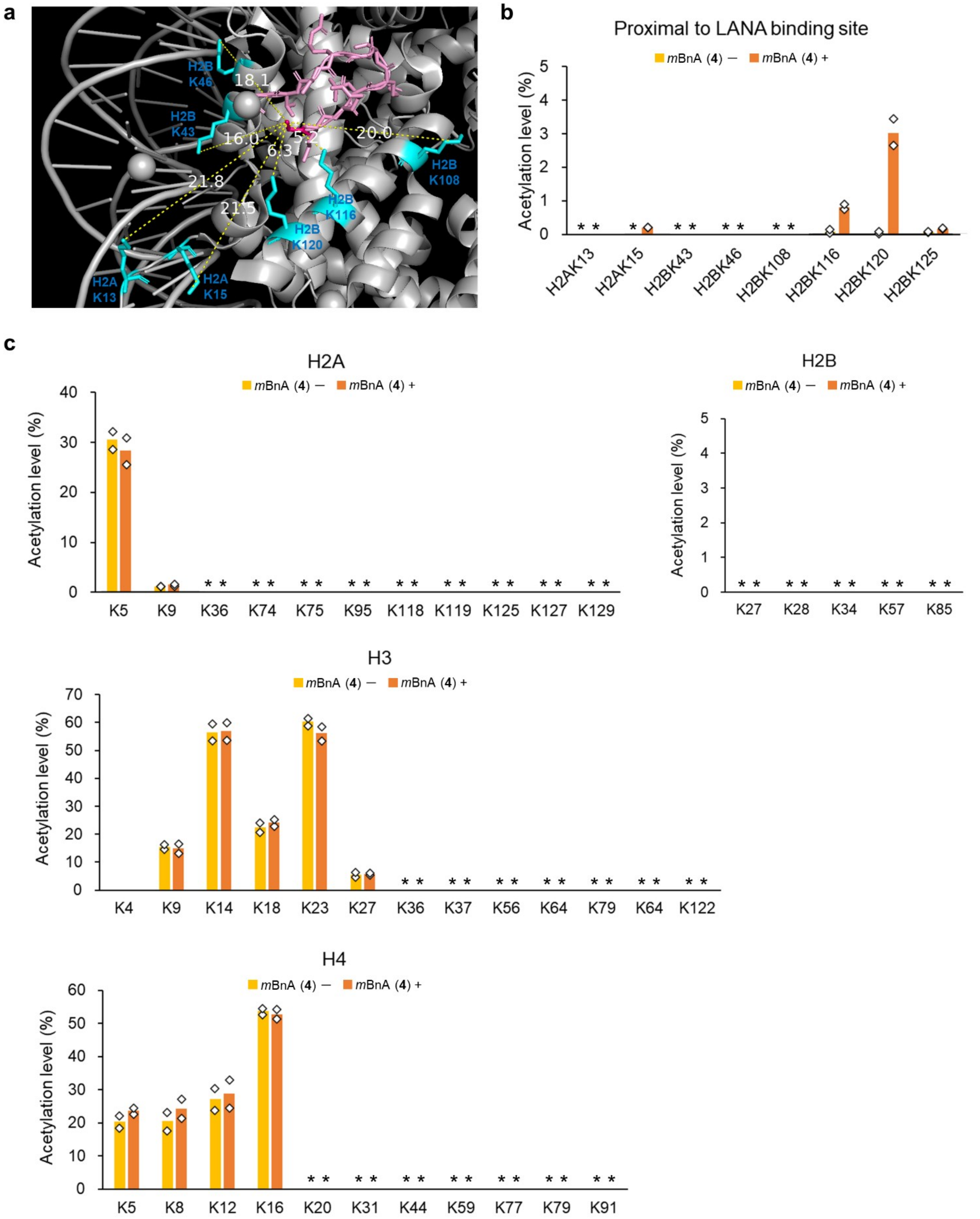

Supplementary Fig. 9 Effects of *m*BnA-TMP(gly$_1$) catalyst (4)-treatment on histone acetylation levels in cells

LieD-transfected HEK293T cells were treated with **4** (100 μM) for 10 h. Histone proteins were acid-extracted, and acetylation levels at the indicated histone lysine residues were quantified with LC–MS/MS analysis. **a** The histone lysine residues within 25 Å from *N*-terminus of LANA peptide (residue 5-15, indicated in pink) are indicated in cyan (PDB: 5GTC). H2BK125 is not shown in this figure but is also present within 25 Å (PDB: 1ZLA). **b** The acetylation levels at the lysine residues in **a** and H2BK125, which are proximal to the LANA binding site. **c** The acetylation levels of other histone lysine residues. H2BK5, 11, 12, 15, 16, 20, 23, 24, and H3K115 were not analyzed due to the difficulty in detecting the digested peptide with LC–MS/MS. The mean chemical yields of the two independent experiments are shown. The overlaid dot plots present the individual data points of each experiment. The asterisks denote “not detected”. Source data are provided as a Source Data file.

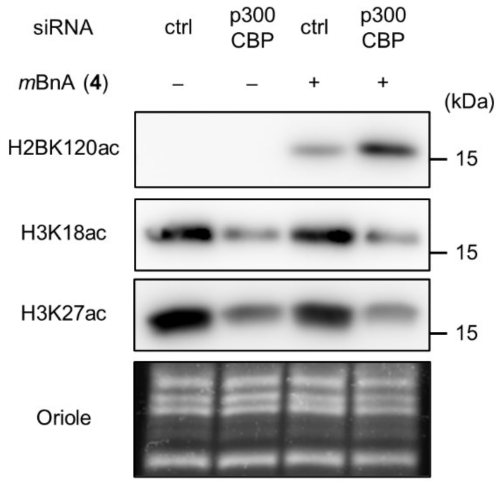

Supplementary Fig. 10 HAT enzyme-independency of H2BK120 acetylation in cells

For knockdown of H2BK120ac writer enzymes p300 (KAT3B) and CBP (KAT3A), HEK293T cells were transfected with control or p300- and CBP-specific siRNA. After transfection of the LieD protein plasmid, the cells were treated with catalyst **4** (100 μM) for 10 h. H2BK120 acetylation, H3K18 acetylation, and H3K27 acetylation in the whole-cell extract were detected with anti-H2BK120ac, anti-H3K18ac, and anti-H3K27ac antibodies, respectively, by western blot analysis. Total histone proteins were visualized with Oriole staining. Representative data from two independent experiments are shown. The reduction of acetylation levels at p300/CBP target lysine residues H3K18 and H3K27[5,6] demonstrates the success of RNAi-mediated inhibition.

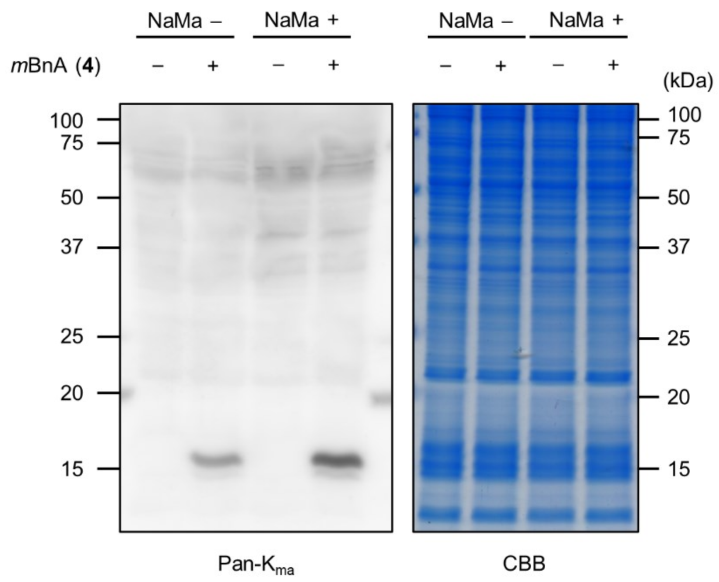

Supplementary Fig. 11 Histone-protein selectivity of *m*BnA-TMP(gly$_1$) catalyst (4)-promoted acylation in cells

LieD-transfected HEK293T cells were incubated in the presence or absence of disodium malonate (NaMa, 20 mM) and then treated with **4** (100 μM) for 10 h. Malonylated proteins in the whole-cell extract were detected using pan-K$_{ma}$ antibody by western blot analysis. Total proteins were visualized with CBB staining. Representative data from two independent experiments are shown.

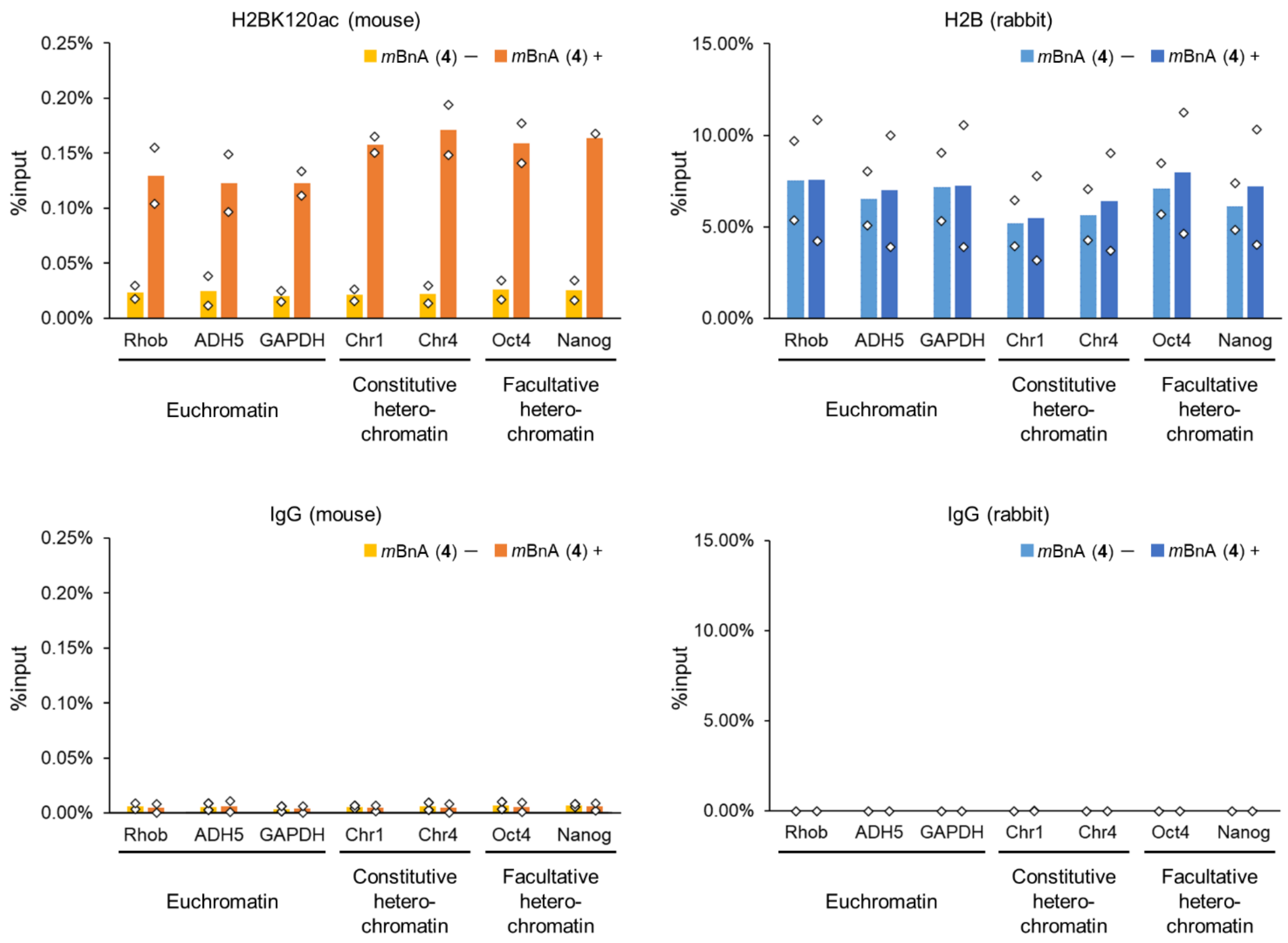

Supplementary Fig. 12 ChIP-qPCR analysis of H2BK120 acetylation levels at several gene regions promoted by *m*BnA-TMP(gly$_1$) catalyst (4)

LieD-transfected HEK293T cells were treated with **4** (100 μM) for 10 h, and were analyzed by ChIP assays using anti-H2BK120ac, anti-H2B, normal mouse IgG, and normal rabbit IgG antibodies. Immunoprecipitated DNA was assessed by real-time PCR using primers specific for indicated gene locus. The mean chemical yields of the two independent experiments are shown. The overlaid dot plots present the individual data points of each experiment. Source data are provided as a Source Data file.

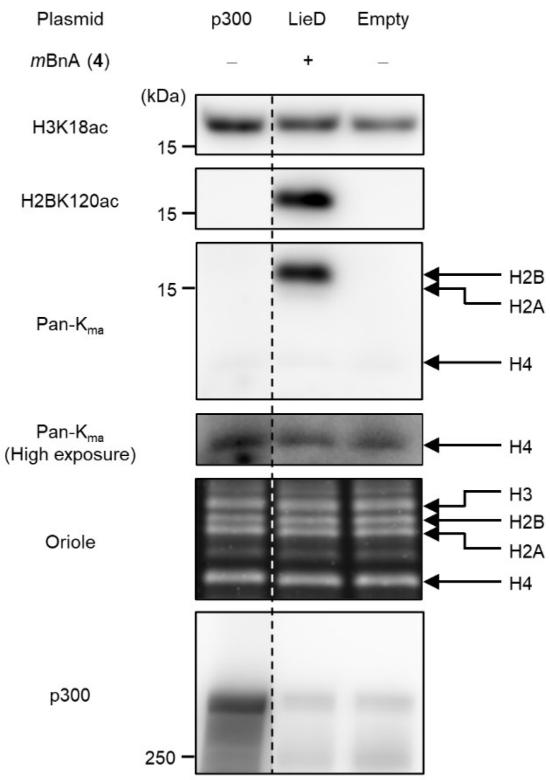

Supplementary Fig. 13 Comparison of *m*BnA- and p300-mediated histone acylations in cells. HEK293T cells were transfected with the indicated plasmids, and then treated with or without *m*BnA-TMP(gly$_1$) (**4**, 100 μM) for 10 h. Acetylated H3K18 and H2BK120, malonylated histones, and p300 in the whole-cell extract were detected using anti-H3K18ac, anti-H2BK120ac, pan-K$_{ma}$, and anti-p300 antibodies, respectively, by western blot analysis. Total histone proteins were visualized with Oriole staining. Dividing lines have been used to indicate where noncontiguous sections of a gel have been aligned for ease of comparison. Representative data from two independent experiments are shown.

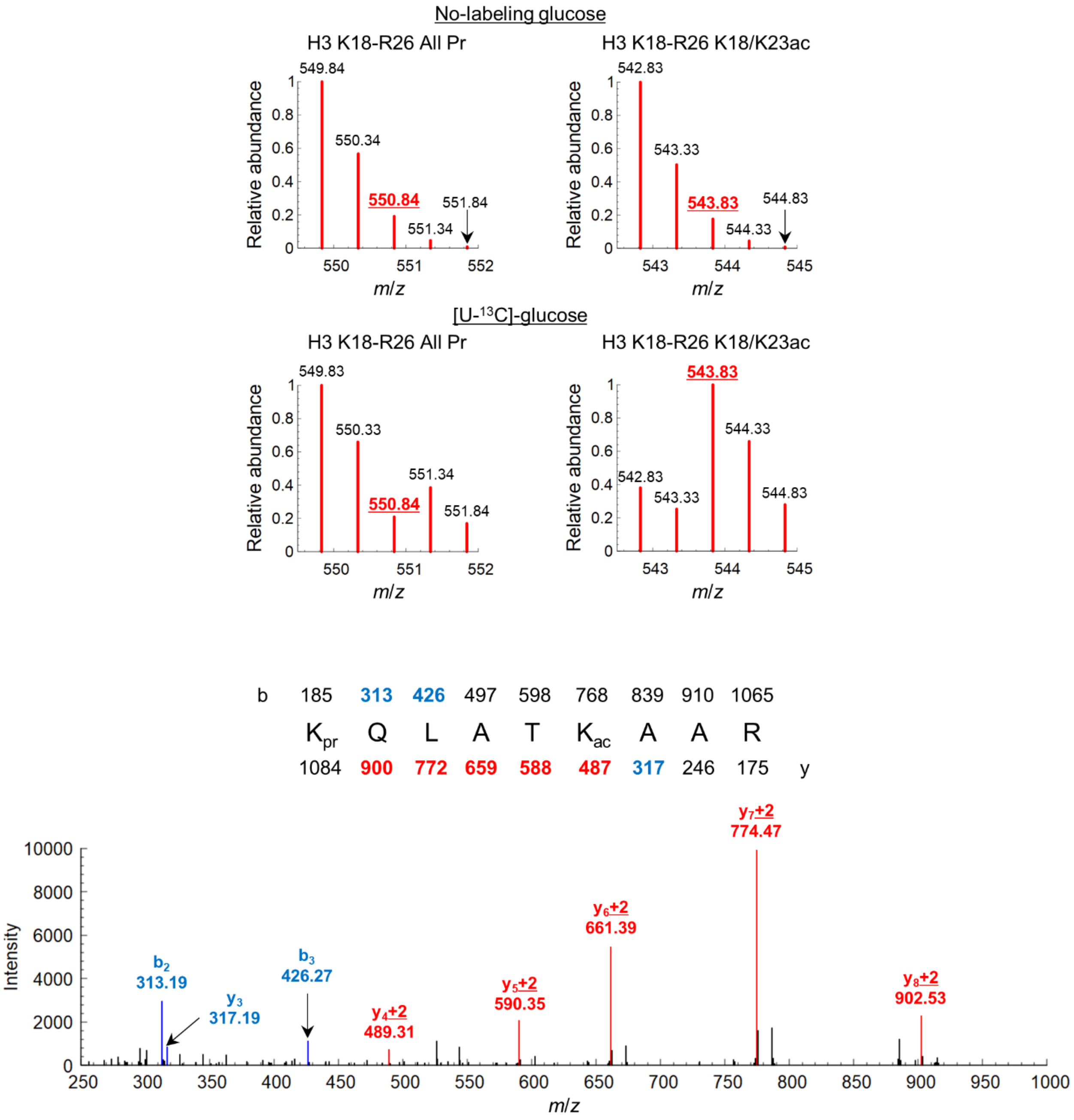

Supplementary Fig. 14 Detection of isotopic labeling at H3K23Ac as a positive control of endogenous Ac-CoA labeling

The analysis was conducted as in **Fig. 4c–d**. Since H3K23 is a target lysine residue of a histone acetyl transferase, the observed +2 Da shift indicates that endogenous Ac-CoA was labeled with ^{13}C. Source data are provided as a Source Data file.

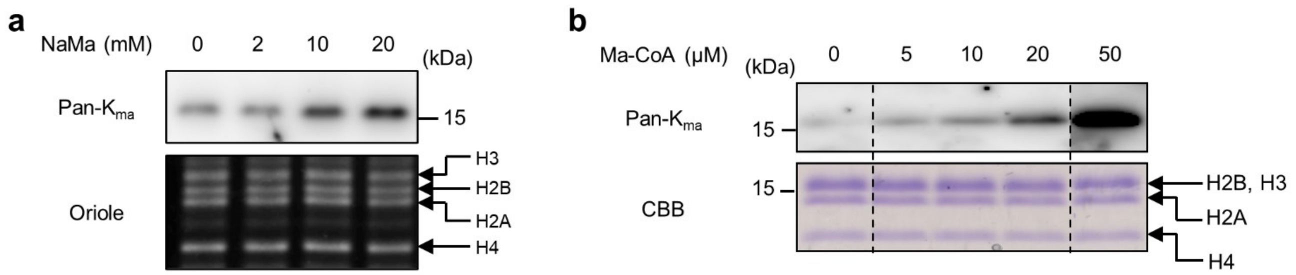

Supplementary Fig. 15 Ma-CoA concentration-dependence of histone malonylation by *m*BnA-TMP(gly$_1$) catalyst (4)

a Dependence of histone malonylation mediated by **4** under various concentrations of NaMa. The culture media of LieD-transfected cells containing 1 g/L glucose were replaced with the media supplemented with the indicated concentrations of NaMa. After 24 h of incubation, the cells were treated with *m*BnA-TMP(gly$_1$) (**4**, 100 μM) for 10 h. Malonylated histones in the whole-cell extract were detected using pan-K$_{ma}$ antibody by western blot analysis. Total histone proteins were visualized with Oriole staining. Representative data of three independent experiments are shown. **b** Ma-CoA concentration-dependence of histone malonylation by **4** in test tube reaction. Recombinant nucleosome (0.35 μM) was reacted with *m*BnA-TMP(gly$_1$) **4** (5 μM) in the presence of LieD-protein ligand (2 μM), the indicated concentration of Ma-CoA, and TCEP (200 μM) at 37 °C for 10 h, and its malonylation was detected with pan-K$_{ma}$ antibody by western blot analysis. Total proteins were visualized by CBB staining. Dividing lines have been used to indicate where noncontiguous sections of a gel have been aligned for ease of comparison. Representative data from two independent experiments are shown.

a

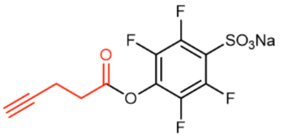

b

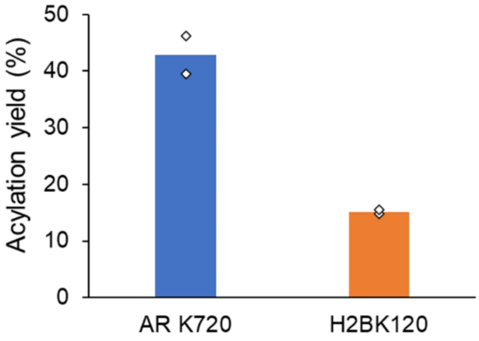

Supplementary Fig. 16 Comparison of the reactivity between AR K720 of androgen receptor and H2BK120 of histone

a Chemical structure of the amine-reactive sulfotetrafluorophenyl (STP) pentynoate probe for quantification of lysine reactivity[7]. **b** Quantitative lysine reactivity analysis. Recombinant androgen receptor (AR, 0.35 μM) and nucleosome (0.175 μM, 0.35 μM as H2BK120) were incubated with STP pentynoate (1 mM) at room temperature for 1 h, and acylation yield of each lysine residue was determined by liquid chromatography-tandem mass spectrometry (LC–MS/MS) analysis. The mean chemical yields of the two independent experiments are shown. The overlaid dot plots present the individual data points of each experiment. Source data are provided as a Source Data file.

Supplementary Methods

General

NMR spectra were recorded on JEOL JNM-ECX500 (500 MHz for ^{1}H NMR, 126 MHz for ^{13}C NMR) or JEOL ECS400 (392 MHz for ^{1}H NMR, 99 MHz for ^{13}C NMR) spectrometer. Chemical shifts were reported in ppm on the δ scale relative to residual $CHCl_3$ (δ = 7.24 for ^{1}H NMR and δ = 77.0 for ^{13}C NMR), CHD_2OD (δ = 3.31 for ^{1}H NMR and δ = 49.0 for ^{13}C NMR), and $CHD_2S(O)CD_3$ (δ = 2.49 for ^{1}H NMR and 39.5 for ^{13}C NMR) as an internal reference, respectively. Analytical HPLC was conducted by using a JASCO HPLC system equipped with a UV-2075 spectrometer, PU-2080 pumps, a DG-2080-54 degasser, and an MX-2080-32 mixer. Preparative HPLC was conducted by using a JASCO HPLC system equipped with a UV-2075 spectrometer, PU-2086 pumps, a DG-2080-53 degasser, an MX-2080-32 mixer or Shimadzu HPLC system equipped with an SPD-20A spectrometer, and LC-6AD pumps. ESI-MS spectra were measured on Agilent Technologies 6120 (for LC/MS), and Bruker micrOTOF II spectrometer (for HRMS). Absorption spectra were measured on Shimadzu UV-1800. LC–MS/MS analyses were conducted with an AB Sciex Triple TOF 4600 equipped with an Eksigent ekspert microLC 200.

Materials

Thin layer chromatography (TLC) was performed on Merck Kieselgel 60 F254 (0.25 mm, 1 mm) plates. Column chromatography was performed with Kanto silica gel 60 (40-50 mesh) or Yamazen UNIVERSAL Premium packed silica-gel. Ac-CoA sodium salt was purchased from nacalai tesque (Kyoto, Japan). [U-^{13}C]-glucose was purchased from SHOKO SCIENCE (Tokyo, Japan). STP ester probe (Pentynoic acid STP) was purchased from Funakoshi (Tokyo, Japan). Other chemicals were used as received from commercial sources (Sigma-Aldrich Japan (Tokyo, Japan), Tokyo chemical Industry (Tokyo, Japan), Kanto chemical (Tokyo, Japan), FUJIFILM Wako Pure Chemical Corporation (Osaka, Japan) or Combi-Blocks (San Diego, CA, USA)), unless otherwise stated. DSH-TMP(gly$_2$) (**S3**)[8], Ph-HXA-TMP(gly$_2$) (**S4**)[1], TMP(gly$_2$)-NH_2 (**S12**)[8], TMP(gly$_1$)-NH_2 (**S31**)[8], 2-(2-(2-methoxyethoxy)ethoxy)ethan-1-amine (**S43**)[9], and 2-mercaptophenylacetic acid (**S44**)[4] were synthesized as reported.

LC/MS

LC/MS was performed using YMC-Triart C18 (2.0 mm I.D. x 50 mm) column at 40 °C with a gradient of acetonitrile in 0.1% aqueous formic acid listed below at a flow rate of 0.2 mL/min.
Method: 2% Acetonitrile for 2 min, followed by a linear gradient of 2-90% acetonitrile over 13 min. UV: 230 nm.

Analytical HPLC

Analytical HPLC was performed using YMC-Pack PROTEIN-RP (4.6 mm I.D. x 150 mm) column at 40 °C with 2% acetonitrile in 0.1% aqueous TFA for 3 min, followed by a linear gradient of 2-90% acetonitrile over 12 min at a flow rate of 1 mL/min. UV: 230 nm.

Preparative HPLC

Preparative HPLC was performed using YMC-Triart C18 (20 mm I.D. x 250 mm) column at 40 °C with a gradient of acetonitrile in 0.1% aqueous TFA listed below at a flow rate of 10 mL/min.

Method: 20% Acetonitrile for 5 min, followed by a linear gradient of 20-100% acetonitrile over 80 min. UV: 230 nm.

Synthetic procedures

Synthesis of *p*HXA-TMP(gly$_2$) (**1**)

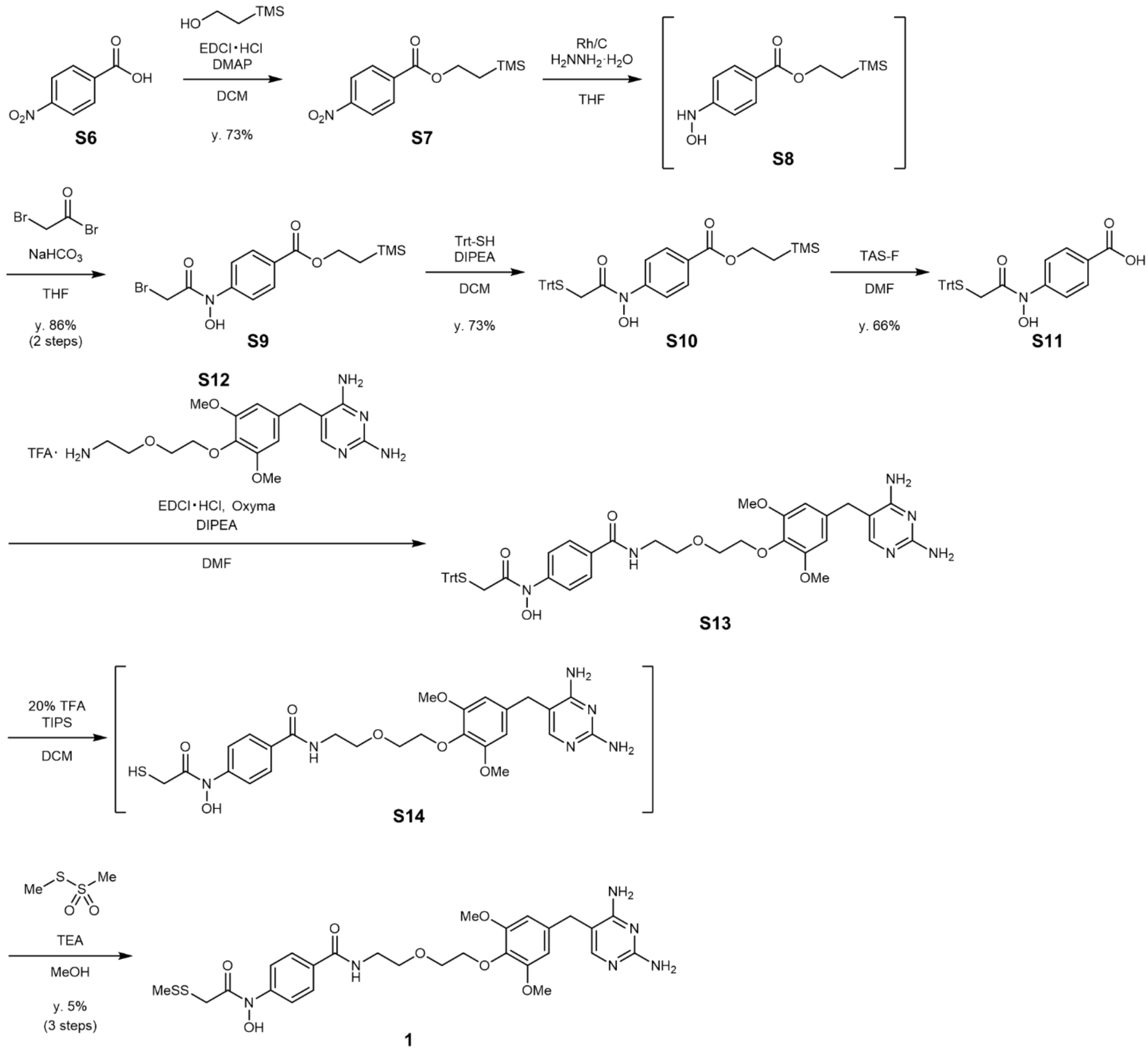

2-(Trimethylsilyl)ethyl 4-nitrobenzoate (S7):

To a stirred solution of **S6** (1.00 g, 5.98 mmol) in DCM (12.0 mL), 2-(trimethylsilyl)ethanol (937 μL, 6.58 mmol), 1-(3-dimethylaminopropyl)-3-ethylcarbodiimide hydrochloride (EDCI・HCl, 1.38 g, 8.18 mmol) and DMAP (147 mg, 1.20 mmol) were added, and the mixture was stirred at r.t. for 45 min. To the reaction mixture, saturated NH_4Cl aq. was added, and the aqueous layer was extracted three times with DCM. Combined organic layers were washed with saturated NaCl aq., dried over Na_2SO_4, filtered, and concentrated to afford crude **S7**, which was purified with silica gel column chromatography (0 to 14% EtOAc/hexane) and then preparative TLC (1 mm plates, EtOAc/hexane = 1/20) to afford **S7** (1.16 g, 4.34 mmol, y. 73%) as colorless oil.

^{1}H NMR ($CDCl_3$, 392 MHz) δ 8.26 (d, *J* = 9.0 Hz, 2H), 8.17 (d, *J* = 9.0 Hz, 2H), 4.44 (t, *J* = 8.5 Hz, 2H), 1.13 (t, *J* = 8.5 Hz, 2H), 0.07 (s, 9H); ^{13}C NMR ($CDCl_3$, 99 MHz) δ 164.8, 150.4, 136.0, 130.6, 123.5, 64.3, 17.4, -1.50; ESI-HRMS: *m/z* calcd for $C_{12}H_{17}NO_4Si$ $[M+Na]^+$: 290.0819. Found: 290.0817.

2-(Trimethylsilyl)ethyl 4-(hydroxyamino)benzoate (S8):

To a stirred solution of **S7** (500 mg, 1.87 mmol) in THF (9.35 mL), rhodium 5% on carbon (50.0 mg, 0.0109 mmol) was added and then cooled to 0 °C. To the mixture, hydrazine monohydrate (109 μL, 2.44 mmol) was added, and the mixture was stirred at 0 °C for 1 h. The reaction mixture was filtered through Celite to afford crude **S8** solution in ca. 30 mL THF, which was used in the next step immediately without further purification.

2-(Trimethylsilyl)ethyl 4-(2-bromo-*N*-hydroxyacetamido)benzoate (S9):

To a stirred solution of crude **S8** in ca. 30 mL THF at 0 °C, $NaHCO_3$ (1.57 g, 18.7 mmol) was added, and the mixture was stirred for 30 min. 2-Bromoacetyl bromide (810 μL, 9.35 mmol) in THF (5.00 mL) was added dropwise, and the mixture was stirred at 0 °C for 45 min. Water was added, and most of THF was removed under reduced pressure. Precipitates were filtered, and then washed with 10% EtOAc/hexane to afford **S9** (602 mg, 1.61 mmol, y. 86% for 2 steps) as white solid.

^{1}H NMR ($CDCl_3$, 500 MHz) δ 8.63 (brs, 1H), 7.89 (br, 2H), 7.62 (br, 2H), 4.37 (t, *J* = 8.2 Hz, 2H), 4.21 (brs, 2H), 1.09 (t, *J* = 8.2 Hz, 2H), 0.05 (s, 9H); ^{13}C NMR ($CDCl_3$, 126 MHz) δ 166.7, 166.4, 144.1, 130.2, 127.1, 119.4, 63.8, 28.2, 17.3, -1.46; ESI-HRMS: *m/z* calcd for $C_{14}H_{20}BrNO_4Si$ $[M+Na]^+$: 396.0237. Found: 396.0232.

2-(Trimethylsilyl)ethyl 4-(*N*-hydroxy-2-(tritylthio)acetamido)benzoate (S10):

To a stirred solution of **S9** (602 mg, 1.61 mmol) in DCM (7.00 mL), triphenylmethanethiol (534 mg, 1.93 mmol) and diisopropylethylamine (DIPEA, 422 μL, 2.42 mmol) were added, and the mixture was stirred at r.t. for 30 min. The reaction mixture was washed with saturated NH_4Cl aq. and saturated NaCl aq., dried over Na_2SO_4, filtered, and concentrated to afford crude **S10**, which was purified with silica gel column chromatography (0 to 2% MeOH/DCM) to afford **S10** (702 mg, 4.34 mmol, y. 73%) as pale orange solid.

^{1}H NMR (DMSO-d_6, 500 MHz) δ 10.89 (s, 1H), 7.94 (d, *J* = 8.3 Hz, 2H), 7.74 (d, *J* = 8.3 Hz, 2H), 7.35 (m, 12H), 7.24 (t, *J* = 6.3 Hz, 3H), 4.35 (t, *J* = 7.4 Hz, 2H), 3.31 (s, 2H), 1.07 (t, *J* = 7.4 Hz, 2H), 0.04 (s, 9H); ^{13}C NMR (DMSO-d_6, 126 MHz) δ 168.2, 165.2, 144.9, 144.0, 129.7, 129.1, 128.1, 126.9, 125.6, 118.7, 66.0, 62.6, 36.0, 16.9, -1.44; ESI-HRMS: *m/z* calcd for $C_{33}H_{35}NO_4SSi$ $[M+Na]^+$: 592.1948. Found: 592.1943.

4-(*N*-Hydroxy-2-(tritylthio)acetamido)benzoic acid (S11):

To a stirred solution of **S10** (400 mg, 0.762 mmol) in DMF (4.00 mL), tris(dimethylamino)sulfonium difluorotrimethylsilicate (TAS-F, 581 mg, 2.11 mmol) in DMF (2.00 mL) was added, and the mixture was stirred at r.t. for 110 min. To the reaction mixture, saturated NH_4Cl aq. was added, and the aqueous layer

was extracted twice with EtOAc. Combined organic layers were washed with saturated NaCl aq., dried over Na_2SO_4, filtered, and concentrated to afford crude **S11**, which was purified with silica gel column chromatography (4 to 11% MeOH/DCM), and was then washed with 10% EtOAc/hexane to afford **S11** (218 mg, 0.465 mmol, y. 66%) as pale orange solid.

^{1}H NMR (CD_3OD, 500 MHz) δ 7.99 (d, J = 8.0 Hz, 2H), 7.69 (br, 2H), 7.44 (d, J = 6.3 Hz, 6H), 7.29 (dd, J = 7.2, 6.3 Hz, 6H), 7.22 (t, J = 7.2 Hz, 3H), 3.35 (s, 2H); ^{13}C NMR (CD_3OD, 126 MHz) δ 170.7, 169.4, 146.2, 145.7, 132.8, 131.2, 130.7, 129.0, 128.0, 114.3, 68.1, 37.0; ESI-HRMS: *m/z* calcd for $C_{28}H_{23}NO_4S$ $[M+Na]^+$: 492.1240. Found: 492.1240.

***N*-(2-(2-(4-((2,4-Diaminopyrimidin-5-yl)methyl)-2,6-dimethoxyphenoxy)ethoxy)ethyl)-4-(*N*-hydroxy-2-(tritylthio)acetamido)benzamide (S13):**

To a stirred solution of **S12**[8] (60.0 mg, 0.126 mmol) in DMF (2.51 mL), **S11** (64.9 mg, 0.138 mmol), EDCI·HCl (48.2 mg, 0.251 mmol), ethyl cyanohydroxyiminoacetate (Oxyma, 35.7 mg, 0.251 mmol), and DIPEA (87.6 μL, 0.503 mmol) were added, and the mixture was stirred at r.t. for 3 h. To the reaction mixture, water was added, and the aqueous layer was extracted twice with EtOAc. Combined organic layers were washed with saturated NaCl aq., dried over Na_2SO_4, filtered, and concentrated to afford crude **S13**. The crude sample was partially purified with silica gel column chromatography (0 to 17% MeOH/DCM) and then preparative TLC (1 mm plates, MeOH/EtOAc = 1/4) to afford **S13** (23.5 mg), which was used next step without further purification due to difficult isolation.

ESI-HRMS: *m/z* calcd for $C_{45}H_{46}N_6O_7S$ $[M+H]^+$: 815.3221. Found: 815.3230.

***N*-(2-(2-(4-((2,4-Diaminopyrimidin-5-yl)methyl)-2,6-dimethoxyphenoxy)ethoxy)ethyl)-4-(*N*-hydroxy-2-mercaptoacetamido)benzamide (S14):**

To a stirred solution of **S13** (23.5 mg) in DCM (461 μL), triisopropylsilane (TIPS, 17.7 μL, 0.0865 mmol) and TFA (115 μL) were added, and the mixture was stirred at r.t. for 10 min. The reaction mixture was concentrated and dissolved in water. The water layer was washed with ether, and concentrated to afford crude **S14** (13.9 mg), which was used in the next step immediately without further purification.

***N*-(2-(2-(4-((2,4-Diaminopyrimidin-5-yl)methyl)-2,6-dimethoxyphenoxy)ethoxy)ethyl)-4-(*N*-hydroxy-2-(methylsulfinothioyl)acetamido)benzamide (1):**

To a stirred solution of **S14** (13.9 mg) in MeOH (485 μL), *S*-methyl methanesulfonothioate (2.50 μL, 0.0267 mmol) and triethylamine (TEA, 11.9 μL, 0.0850 mmol) were added, and the mixture was stirred at r.t. for 80 min. The mixture was concentrated to afford crude **1**, which was purified with preparative HPLC (20-100% MeCN in 0.1% TFA/H_2O) to afford **1** (4.2 mg, 0.0068 mmol, y. 5% for 3 steps) as pale pink solid.

^{1}H NMR (CD_3OD, 60 °C, 500 MHz) δ 7.83 (d, J = 8.6 Hz, 2H), 7.71 (d, J = 8.6 Hz, 2H), 7.26 (s, 1H), 6.54 (s, 2H), 4.10 (t, J = 4.7 Hz, 2H), 3.91 (s, 2H), 3.79 (t, J = 4.7 Hz, 2H), 3.77 (s, 6H), 3.73 (t, J = 5.2 Hz, 2H), 3.67 (s, 2H), 3.60 (t, J = 5.2 Hz, 2H), 2.47 (s, 3H); ^{13}C NMR (DMSO-d_6, 60 °C, 126 MHz) δ 168.1, 165.5,

163.9, 154.2, 152.8, 143.3, 139.6, 135.6, 132.7, 130.3, 127.3, 118.8, 108.7, 106.6, 71.5, 69.3, 68.7, 55.9, 41.8, 31.8, 30.1, 22.3; ESI-HRMS: *m/z* calcd for $C_{27}H_{34}N_6O_7S_2$ $[M+H]^+$: 619.2003. Found: 619.1999.

Synthesis of *m*HXA-TMP(gly$_2$) (**2**)

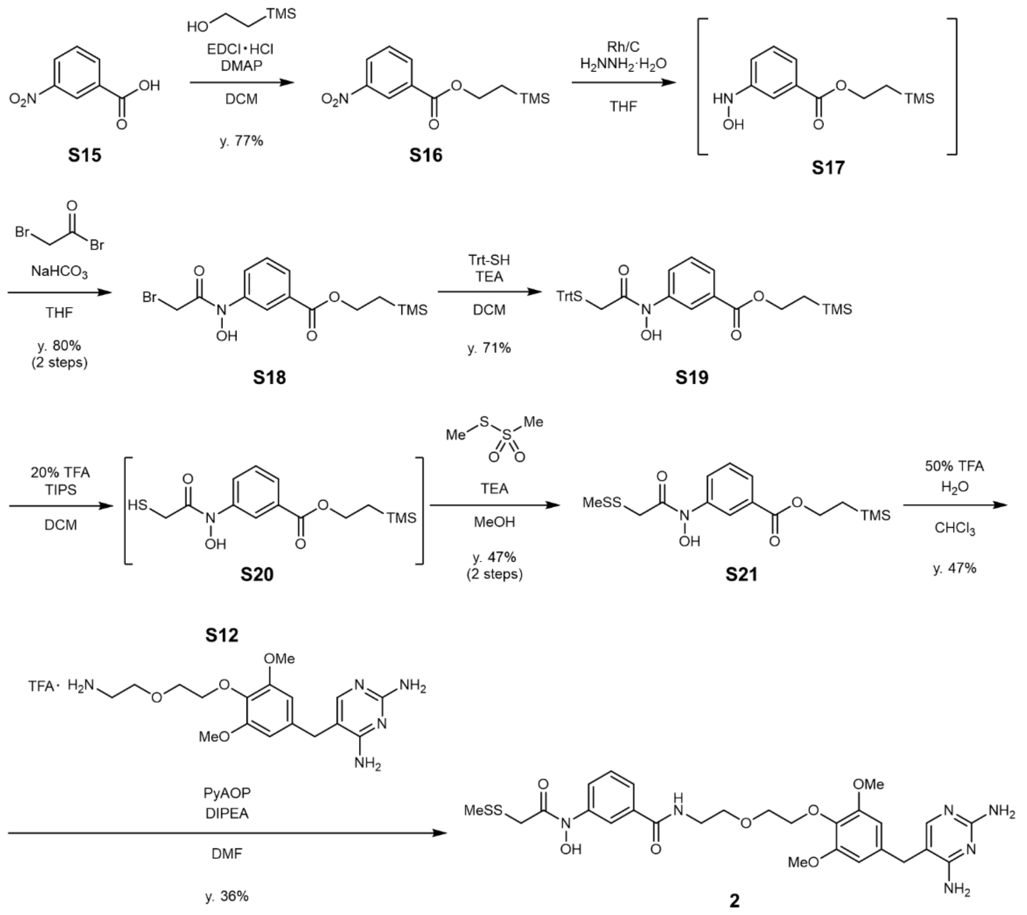

2-(Trimethylsilyl)ethyl 3-nitrobenzoate (S16):

To a stirred solution of **S15** (500 mg, 2.99 mmol) in DCM (60.0 mL), 2-(trimethylsilyl)ethanol (760 μL, 3.29 mmol), EDCI·HCl (688 mg, 3.59 mmol) and DMAP (439 mg, 3.59 mmol) were added, and the mixture was stirred at r.t. for 1.5 h. To the reaction mixture, water was added, and the aqueous layer was extracted twice with DCM. Combined organic layers were washed with saturated NaCl aq., dried over Na_2SO_4, filtered, and concentrated to afford crude **S16**, which was purified with silica gel column chromatography (10 to 40% EtOAc/hexane) to afford **S16** (613 mg, 2.29 mmol, y. 77%) as colorless oil.

^{1}H NMR ($CDCl_3$, 500 MHz) δ 8.83 (s, 1H), 8.38 (d, *J* = 8.0 Hz, 1H), 8.34 (d, *J* = 8.0 Hz, 1H), 7.63 (dd, *J* = 8.0, 8.0 Hz, 1H), 4.51 (t, *J* = 8.4 Hz, 2H), 1.15 (t, *J* = 8.4 Hz, 2H), 0.07 (s, 9H); ^{13}C NMR ($CDCl_3$, 126 MHz) δ 164.6, 148.2, 135.2, 132.4, 129.5, 127.2, 124.5, 64.3, 17.4, -1.51; ESI-HRMS: *m/z* calcd for $C_{12}H_{17}NO_4Si$ $[M+Na]^+$: 290.0819. Found: 290.0824.

2-(Trimethylsilyl)ethyl 3-(hydroxyamino)benzoate (S17):

To a stirred solution of **S16** (587 mg, 2.20 mmol) in THF (11.0 mL), rhodium 5% on carbon (118 mg, 0.0257 mmol) was added and then cooled to 0 °C. To the mixture, hydrazine monohydrate (256 μL, 5.27 mmol) was added, and the mixture was stirred at 0 °C for 1 h. The reaction mixture was filtered through Celite to

afford crude **S17** solution in ca. 30 mL THF, which was used in the next step immediately without further purification.

2-(Trimethylsilyl)ethyl 3-(2-bromo-*N*-hydroxyacetamido)benzoate (S18):

To a stirred solution of crude **S17** in ca. 30 mL THF at 0 °C, $NaHCO_3$ (1.85 g, 22.0 mmol) was added, and the mixture was stirred for 45 min. 2-Bromoacetyl bromide (952 μL, 11.0 mmol) in THF (3.00 mL) was added dropwise, and the mixture was stirred at 0 °C for 50 min. To the reaction mixture, water was added, and most of the THF was removed under reduced pressure. The mixture was extracted twice with EtOAc, washed with saturated NaCl aq., dried over Na_2SO_4, filtered, and concentrated to afford crude **S18**, which was purified with silica gel column chromatography (1st: 20 to 50% EtOAc/hexane, 2nd: 0 to 20% MeOH/DCM, 3rd: 0 to 10% MeOH/DCM, 4th: 17 to 33% EtOAc/hexane) to afford **S18** (654 mg, 1.75 mmol, y. 80% for 2 steps) as colorless oil.

^{1}H NMR (DMSO-d_6, 60 °C, 500 MHz) δ 8.27 (s, 1H), 7.91 (d, *J* = 7.7 Hz, 1H), 7.76 (d, *J* = 8.0 Hz, 1H), 7.52 (dd, *J* = 8.0, 7.7 Hz, 1H), 4.40 (t, *J* = 8.2 Hz, 2H), 4.37 (s, 2H), 1.10 (t, *J* = 8.2 Hz, 2H), 0.06 (s, 9H); ^{13}C NMR (DMSO-d_6, 60 °C, 126 MHz) δ 172.1, 165.4, 141.7, 130.4, 128.6, 124.7, 123.4, 119.6, 62.7, 60.3, 16.9, -1.63; ESI-HRMS: *m/z* calcd for $C_{14}H_{20}BrNO_4Si$ $[M+Na]^+$: 396.0237. Found: 396.0224.

2-(Trimethylsilyl)ethyl 3-(*N*-hydroxy-2-(tritylthio)acetamido)benzoate (S19):

To a stirred solution of **S18** (654 mg, 1.75 mmol) in DCM (35.0 mL), triphenylmethanethiol (532 mg, 1.92 mmol) and TEA (731 μL, 5.25 mmol) were added, and the mixture was stirred at r.t. for 100 min. To the reaction mixture, water was added, and the aqueous layer was extracted twice with EtOAc. Combined organic layers were washed with saturated NaCl aq., dried over Na_2SO_4, filtered, and concentrated to afford crude **S19**, which was purified with silica gel column chromatography (1st: 10 to 45% EtOAc/hexane, 2nd: 0 to 5% MeOH/DCM, 3rd: 10 to 45% EtOAc/hexane) to afford **S19** (707 mg, 1.24 mmol, y. 71%) as orange solid.

^{1}H NMR (DMSO-d_6, 60 °C, 500 MHz) δ 8.22 (s, 1H), 7.83 (d, *J* = 8.0 Hz, 1H), 7.75 (d, *J* = 7.7 Hz, 1H), 7.48 (dd, *J* = 8.0, 7.7 Hz, 1H), 7.40 (d, *J* = 7.7 Hz, 6H), 7.33 (dd, *J* = 7.7, 7.3 Hz, 6H), 7.24 (t, *J* = 7.3 Hz, 3H), 4.41 (t, *J* = 8.7 Hz, 2H), 3.34 (s, 2H), 1.11 (t, *J* = 8.7 Hz, 2H), 0.07 (s, 9H); ^{13}C NMR (DMSO-d_6, 60 °C, 126 MHz) δ 167.6, 165.1, 143.9, 141.3, 130.3, 128.8, 128.4, 127.7, 126.5, 125.1, 124.3, 120.5, 66.0, 62.6, 35.4, 16.7, -1.76; ESI-HRMS: *m/z* calcd for $C_{33}H_{35}NO_4SSi$ $[M+Na]^+$: 592.1948. Found: 592.1949.

2-(Trimethylsilyl)ethyl 3-(*N*-hydroxy-2-mercaptoacetamido)benzoate (S20):

To a stirred solution of **S19** (170 mg, 0.298 mmol) in DCM (4.77 mL), TIPS (183 μL, 0.895 mmol) and TFA (1.19 mL) were added, and the mixture was stirred at r.t. for 10 min. The mixture was concentrated. The residue was roughly purified with silica gel column chromatography (0 to 10% MeOH/DCM) to afford crude **S20** (119.6 mg), which was used in the next step immediately without further purification.

2-(Trimethylsilyl)ethyl 3-(*N*-hydroxy-2-(methylsulfinothioyl)acetamido)benzoate (S21):

To a stirred solution of **S20** (97.6 mg) in MeOH (5.96 mL), *S*-methyl methanesulfonothioate (27.9 μL, 0.298 mmol) and TEA (49.9 μL, 0.358 mmol) were added, and the mixture was stirred at r.t. for 1 h. To the reaction mixture, water was added, and the aqueous layer was extracted twice with EtOAc. Combined organic layers were washed with saturated NaCl aq., dried over Na_2SO_4, filtered, and concentrated to afford crude **S21**, which was purified with silica gel column chromatography (20 to 25% EtOAc/hexane) to afford **S21** (52.7 mg, 0.141 mmol, y. 47% for 2 steps) as yellow oil.

^{1}H NMR (DMSO-d_6, 60 °C, 500 MHz) δ 10.84 (brs, 1H), 8.26 (s, 1H), 7.90 (d, *J* = 8.0 Hz, 1H), 7.74 (d, *J* = 7.7 Hz, 1H), 7.52 (dd, *J* = 8.0, 7.7 Hz, 1H), 4.40 (t, *J* = 8.0 Hz, 2H), 3.96 (s, 2H), 2.47 (s, 3H), 1.10 (t, *J* = 8.0 Hz, 2H), 0.06 (s, 9H); ^{13}C NMR (DMSO-d_6, 60 °C, 126 MHz) δ 168.1, 165.2, 141.5, 130.4, 128.6, 125.2, 124.3, 120.4, 62.7, 41.6, 22.4, 16.7, -1.69; ESI-HRMS: *m/z* calcd for $C_{15}H_{23}NO_4S_2Si$ $[M+Na]^+$: 396.0730. Found: 396.0741.

3-(*N*-Hydroxy-2-(methylsulfinothioyl)acetamido)benzoic acid (S22):

A solution of **S21** (45.0 mg, 0.120 mmol) in $CHCl_3$ (1.19 mL), water (23.9 μL) and TFA (1.19 mL) was stirred at 50 °C for 40 min. The mixture was concentrated to afford crude **S22**, which was purified with preparative HPLC (20-100% MeCN in 0.1% TFA/H_2O) to afford **S22** (15.5 mg, 0.0567 mmol, y. 47%) as white solid.

^{1}H NMR (DMSO-d_6, 60 °C, 500 MHz) δ 8.22 (s, 1H), 7.87 (d, *J* = 8.3 Hz, 1H), 7.74 (d, *J* = 7.7 Hz, 1H), 7.50 (dd, *J* = 8.3, 7.7 Hz, 1H), 3.96 (s, 2H), 2.47 (s, 3H); ^{13}C NMR (DMSO-d_6, 60 °C, 126 MHz) δ 168.1, 166.8, 141.4, 131.4, 128.5, 125.6, 124.2, 120.9, 41.6, 22.4; ESI-HRMS: *m/z* calcd for $C_{10}H_{11}NO_4S_2$ $[M+Na]^+$: 296.0022. Found: 296.0028.

***N*-(2-(2-(4-((2,4-Diaminopyrimidin-5-yl)methyl)-2,6-dimethoxyphenoxy)ethoxy)ethyl)-3-(*N*-hydroxy-2-(methylsulfinothioyl)acetamido)benzamide (2):**

To a stirred solution of **S22** (10.6 mg, 0.0388 mmol) in DMF (776 μL), **S12** (20.4 mg, 0.0427 mmol), PyAOP (50.6 mg, 0.0970 mmol) and DIPEA (33.8 μL, 0.194 mmol) were added, and the mixture was stirred at r.t. for 3 h. The mixture was concentrated to afford crude **2**, which was purified with preparative HPLC (20-100% MeCN in 0.1% TFA/H_2O) to afford **2** (8.6 mg, 0.014 mmol, y. 36%) as white solid.

^{1}H NMR (CD_3OD, 60 °C, 392 MHz) δ 8.07 (s, 1H), 7.78 (d, *J* = 7.6 Hz, 1H), 7.65 (d, *J* = 7.9 Hz, 1H), 7.41 (dd, *J* = 7.9, 7.6 Hz, 1H), 7.26 (s, 1H), 6.53 (s, 2H), 4.09 (t, *J* = 4.5 Hz, 2H), 3.88 (s, 2H), 3.81-3.76 (m, 8H), 3.72 (t, *J* = 5.2 Hz, 2H), 3.66 (s, 2H), 3.60 (t, *J* = 5.2 Hz, 2H), 2.47 (s, 3H); ^{13}C NMR (DMSO-d_6, 60 °C, 126 MHz) δ 165.7, 164.0, 154.2, 152.8, 141.3, 140.9, 139.7, 139.2, 135.6, 134.9, 132.7, 128.1, 125.1, 123.4, 108.7, 106.6, 71.6, 69.4, 68.7, 56.0, 41.6, 36.8, 31.9, 22.4; ESI-HRMS: *m/z* calcd for $C_{27}H_{34}N_6O_7S_2$ $[M+H]^+$: 619.2003. Found: 619.1997.

Synthesis of *m*BnA-TMP(gly$_2$) (**3**)

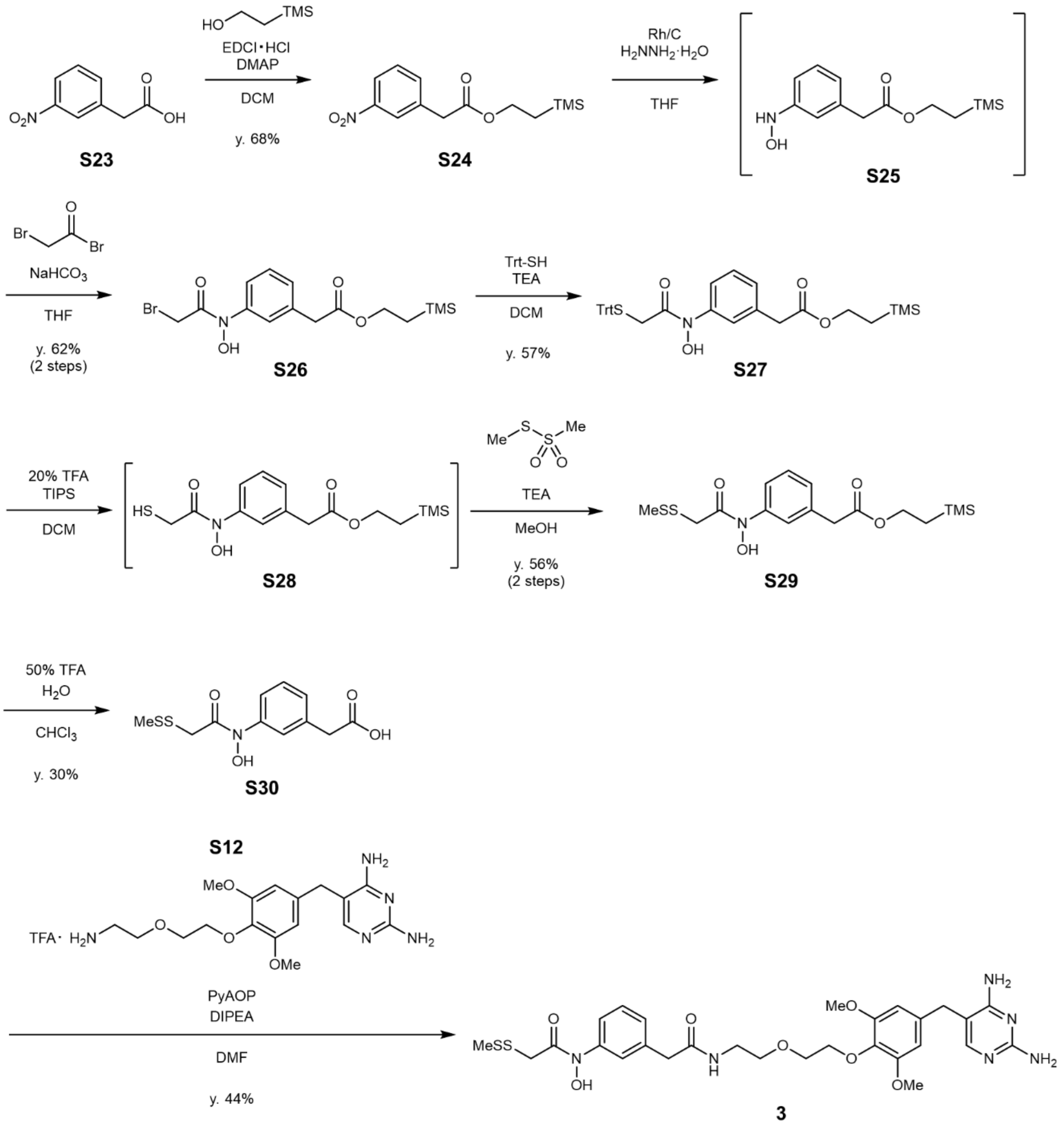

2-(Trimethylsilyl)ethyl 2-(3-nitrophenyl)acetate (S24):

To a stirred solution of **S23** (3.00 g, 16.6 mmol) in DCM (82.8 mL), 2-(trimethylsilyl)ethanol (4.2 mL, 18.2 mmol), EDCI･HCl (3.81 g, 19.9 mmol) and DMAP (2.43 g, 19.9 mmol) were added, and the mixture was stirred at r.t. for 1.5 h. To the reaction mixture, water was added, and the aqueous layer was extracted twice with DCM. Combined organic layers were washed with saturated NaCl aq., dried over Na_2SO_4, filtered, and concentrated to afford crude **S24**, which was purified with silica gel column chromatography (5 to 25% EtOAc/hexane) to afford **S24** (3.15 g, 11.2 mmol, y. 68%) as pale yellow oil.

^{1}H NMR (CDCl$_3$, 500 MHz) δ 8.08 (s, 1H), 8.03 (d, *J* = 8.3 Hz, 1H), 7.56 (d, *J* = 7.7 Hz, 1H), 7.42 (dd, *J* = 8.3, 7.7 Hz, 1H), 4.14 (t, *J* = 8.6 Hz, 2H), 3.65 (s, 2H), 0.93 (t, *J* = 8.6 Hz, 2H), -0.05 (s, 9H); ^{13}C NMR

(CDCl$_3$, 126 MHz) δ 170.2, 148.0, 135.9, 135.4, 129.2, 124.1, 121.9, 63.4, 40.6, 17.0, -1.79; ESI-HRMS: *m/z* calcd for $C_{13}H_{19}NO_4Si$ [M+Na]$^+$: 304.0976. Found: 304.0979.

2-(Trimethylsilyl)ethyl 2-(3-(hydroxyamino)phenyl)acetate (S25):

To a stirred solution of **S24** (3.00 g, 10.7 mmol) in THF (50.0 mL), rhodium 5% on carbon (285 mg, 0.0623 mmol) was added and then cooled to 0 °C. To the mixture, hydrazine monohydrate (1.24 mL, 25.6 mmol) was added, and the mixture was stirred at 0 °C for 30 min. The reaction mixture was filtered through Celite to afford crude **S25** solution in ca. 85 mL THF, which was used in the next step immediately without further purification.

2-(Trimethylsilyl)ethyl 2-(3-(2-bromo-*N*-hydroxyacetamido)phenyl)acetate (S26):

To a stirred solution of crude **S25** in ca. 85 mL THF at 0 °C, $NaHCO_3$ (8.96 g, 107 mmol) was added, and the mixture was stirred for 30 min. 2-Bromoacetyl bromide (2.77 mL, 32.0 mmol) in THF (15.0 mL) was added dropwise, and the mixture was stirred at 0 °C for 1 h. To the reaction mixture, water was added, and most of the THF was removed under reduced pressure. The mixture was extracted twice with EtOAc, washed with saturated NaCl aq., dried over Na_2SO_4, filtered, and concentrated to afford crude **S26**, which was purified with silica gel column chromatography (1st: 20 to 50% EtOAc/hexane, 2nd: 0 to 5% MeOH/DCM) to afford **S26** (2.58 g, 6.64 mmol, y. 62% for 2 steps) as yellow oil.

^{1}H NMR (CDCl$_3$, 60 °C, 392 MHz) δ 8.86 (brs, 1H), 7.42-7.32 (m, 3H), 7.11 (br, 1H), 4.14 (t, *J* = 8.3 Hz, 2H), 3.99 (brs, 2H), 3.53 (s, 2H), 0.94 (t, *J* = 8.3 Hz, 2H), -0.01 (s, 9H); ^{13}C NMR (CDCl$_3$, 60 °C, 126 MHz) δ 171.4, 166.8, 140.2, 137.0, 135.0, 129.5, 128.9, 128.7, 63.3, 58.2, 41.1, 17.3, -1.66; ESI-HRMS: *m/z* calcd for $C_{15}H_{22}BrNO_4Si$ [M+Na]$^+$: 410.0394. Found: 410.0397.

2-(Trimethylsilyl)ethyl 2-(3-(*N*-hydroxy-2-(tritylthio)acetamido)phenyl)acetate (S27):

To a stirred solution of **S26** (1.00 g, 2.58 mmol) in DCM (51.5 mL), triphenylmethanethiol (783 mg, 2.83 mmol) and TEA (1.08 mL, 7.73 mmol) were added, and the mixture was stirred at r.t. for 2 h. To the reaction mixture, water was added, and the aqueous layer was extracted twice with DCM. Combined organic layers were washed with saturated NaCl aq., dried over Na_2SO_4, filtered, and concentrated to afford crude **S27**, which was purified with silica gel column chromatography (1st: 0 to 5% MeOH/DCM, 2nd: 20 to 40% EtOAc/hexane) to afford **S27** (849 mg, 1.45 mmol, y. 57%) as orange oil.

^{1}H NMR (DMSO-*d*$_6$, 60 °C, 500 MHz) δ 10.51 (s, 1H), 7.50 (s, 1H), 7.45 (d, *J* = 8.0 Hz, 1H), 7.39 (d, *J* = 8.0 Hz, 6H), 7.33 (dd, *J* = 8.0, 7.1 Hz, 6H), 7.29 (dd, *J* = 8.0, 7.7 Hz, 1H), 7.25 (t, *J* = 7.1 Hz, 3H), 7.07 (d, *J* = 7.7 Hz, 1H), 4.16 (t, *J* = 8.2 Hz, 2H), 3.62 (s, 2H), 3.28 (s, 2H), 0.96 (t, *J* = 8.2 Hz, 2H), 0.02 (s, 9H); ^{13}C NMR (DMSO-*d*$_6$, 60 °C, 126 MHz) δ 180.8, 170.5, 143.9, 141.1, 134.5, 128.8, 128.0, 127.7, 127.4, 126.5, 125.8, 123.0, 65.9, 62.0, 40.4, 35.4, 16.6, -1.81; ESI-HRMS: *m/z* calcd for $C_{34}H_{37}NO_4SSi$ [M+Na]$^+$: 606.2105. Found: 606.2100.

2-(Trimethylsilyl)ethyl 2-(3-(*N*-hydroxy-2-mercaptoacetamido)phenyl)acetate (S28):

To a stirred solution of **S27** (380 mg, 0.651 mmol) in DCM (10.4 mL), TIPS (400 μL, 1.95 mmol) and TFA (2.60 mL) were added, and the mixture was stirred at r.t. for 10 min. The mixture was concentrated. The residue was roughly purified with silica gel column chromatography (0 to 10% MeOH/DCM) to afford crude **S28** (278.5 mg), which was used in the next step immediately without further purification.

2-(Trimethylsilyl)ethyl 2-(3-(*N*-hydroxy-2-(methylsulfinothioyl)acetamido)phenyl)acetate (S29):

To a stirred solution of **S28** (278.5 mg) in MeOH (13.0 mL), *S*-methyl methanesulfonothioate (60.9 μL, 0.651 mmol) and TEA (109 μL, 0.781 mmol) were added, and the mixture was stirred at r.t. for 1 h. To the reaction mixture, water was added, and the aqueous layer was extracted twice with EtOAc. Combined organic layers were washed with saturated NaCl aq., dried over Na_2SO_4, filtered, and concentrated to afford crude **S29**, which was purified with silica gel column chromatography (20 to 50% EtOAc/hexane) to afford **S29** (140 mg, 0.362 mmol, y. 56% for 2 steps) as pale orange oil.

^{1}H NMR (DMSO-d_6, 60 °C, 500 MHz) δ 10.65 (s, 1H), 7.57 (s, 1H), 7.54 (d, *J* = 8.0 Hz, 1H), 7.32 (dd, *J* = 8.0, 7.4 Hz, 1H), 7.08 (d, *J* = 7.4 Hz, 1H), 4.16 (t, *J* = 8.2 Hz, 2H), 3.94 (s, 2H), 3.64 (s, 2H), 2.47 (s, 3H), 0.96 (t, *J* = 8.2 Hz, 2H), 0.03 (s, 9H); ^{13}C NMR (DMSO-d_6, 60 °C, 126 MHz) δ 170.5, 167.5, 141.3, 134.6, 128.1, 125.8, 121.1, 118.9, 62.0, 41.7, 40.4, 22.4, 16.7, -1.78; ESI-HRMS: *m/z* calcd for $C_{16}H_{25}NO_4S_2Si$ $[M+Na]^+$: 410.0886. Found: 410.0889.

2-(3-(*N*-Hydroxy-2-(methylsulfinothioyl)acetamido)phenyl)acetic acid (S30):

A solution of **S30** (100 mg, 0.258 mmol) in $CHCl_3$ (2.55 mL), water (51.1 μL) and TFA (2.55 mL) was stirred at 50 °C for 40 min. The mixture was concentrated to afford crude **S30**, which was purified with preparative HPLC (20-100% MeCN in 0.1% TFA/H_2O) to afford **S30** (22.3 mg, 0.0776 mmol, y. 30%) as white solid.

^{1}H NMR (DMSO-d_6, 60 °C, 500 MHz) δ 7.56 (s, 1H), 7.53 (d, *J* = 8.0 Hz, 1H), 7.32 (dd, *J* = 8.0, 7.4 Hz, 1H), 7.09 (d, *J* = 7.4 Hz, 1H), 3.94 (s, 2H), 3.59 (s, 2H), 2.47 (s, 3H); ^{13}C NMR (DMSO-d_6, 60 °C, 126 MHz) δ 172.1, 167.6, 141.3, 135.3, 128.2, 126.1, 121.4, 119.1, 41.7, 40.7, 22.5; ESI-HRMS: *m/z* calcd for $C_{11}H_{13}NO_4S_2$ $[M+Na]^+$: 310.0178. Found: 310.0180.

***N*-(3-(2-((2-(2-(4-((2,4-Diaminopyrimidin-5-yl)methyl)-2,6-dimethoxyphenoxy)ethoxy)ethyl)amino)-2-oxoethyl)phenyl)-*N*-hydroxy-2-(methylsulfinothioyl)acetamide (3):**

To a stirred solution of **S30** (10.0 mg, 0.0348 mmol) in DMF (696 μL), **S12** (18.3 mg, 0.0383 mmol), PyAOP (45.4 mg, 0.0870 mmol) and DIPEA (30.3 μL, 0.174 mmol) were added, and the mixture was stirred at r.t. for 4.5 h. The mixture was concentrated to afford crude **3**, which was purified with preparative HPLC (20-100% MeCN in 0.1% TFA/H_2O) to afford **3** (9.7 mg, 0.015 mmol, y. 44%) as white solid.

^{1}H NMR (CD_3OD, 60 °C, 392 MHz) δ 7.54 (s, 1H), 7.48 (d, *J* = 7.6 Hz, 1H), 7.30 (dd, *J* = 7.6, 7.2 Hz, 1H), 7.25 (s, 1H), 7.17 (d, *J* = 7.2 Hz, 1H), 6.56 (s, 2H), 4.06 (t, *J* = 4.6 Hz, 2H), 3.80 (s, 6H), 3.78 (brs, 2H), 3.72 (t, *J* = 4.6 Hz, 2H), 3.67 (s, 2H), 3.59 (t, *J* = 5.4 Hz, 2H), 3.54 (s, 2H), 3.38 (t, *J* = 5.4 Hz, 2H), 2.45 (s, 3H); ^{13}C NMR (DMSO-d_6, 60 °C, 126 MHz) δ 170.0, 167.6, 164.1, 154.4, 153.0, 141.2, 139.7, 136.7, 135.8,

132.8, 128.0, 125.9, 121.3, 118.9, 108.8, 106.7, 71.7, 69.5, 69.0, 56.0, 48.4, 42.2, 41.8, 32.0, 22.4; ESI-HRMS: *m/z* calcd for $C_{28}H_{36}N_6O_7S_2$ $[M+H]^+$: 633.2160. Found: 633.2162.

Synthesis of *m*BnA-TMP(gly$_1$) (**4**)

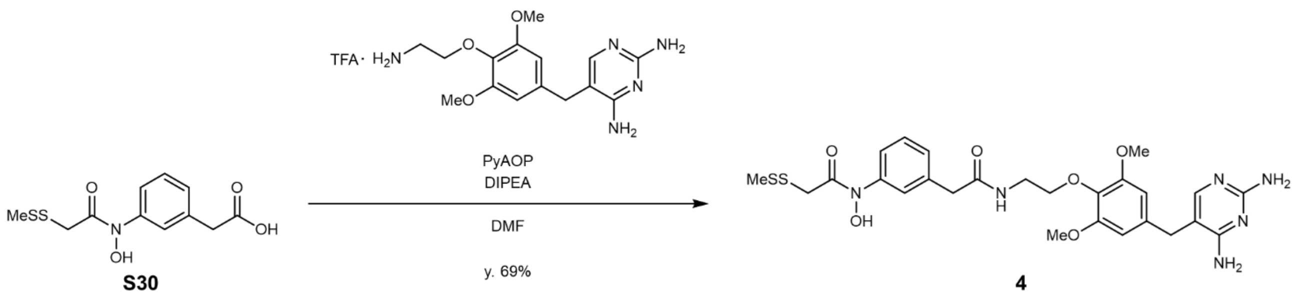

***N*-(3-(2-((2-(4-((2,4-Diaminopyrimidin-5-yl)methyl)-2,6-dimethoxyphenoxy)ethyl)amino)-2-oxoethyl)phenyl)-*N*-hydroxy-2-(methylsulfinothioyl)acetamide (4):**

To a stirred solution of **S30** (10.0 mg, 0.0348 mmol) in DMF (696 μL), **S31**[8] (16.6 mg, 0.0383 mmol), PyAOP (45.4 mg, 0.0870 mmol) and DIPEA (30.3 μL, 0.174 mmol) were added, and the mixture was stirred at r.t. for 4.5 h. The mixture was concentrated to afford crude **4**, which was purified with preparative HPLC (20-100% MeCN in 0.1% TFA/H_2O) to afford **4** (14.1 mg, 0.0240 mmol, y. 69%) as white solid.

^{1}H NMR (CD_3OD, 60 °C, 500 MHz) δ 7.57 (s, 1H), 7.50 (d, *J* = 8.0 Hz, 1H), 7.34 (dd, *J* = 8.0, 7.2 Hz, 1H), 7.26 (s, 1H), 7.19 (d, *J* = 7.2 Hz, 1H), 6.55 (s, 2H), 4.00 (t, *J* = 5.4 Hz, 2H), 3.83 (brs, 2H), 3.78 (s, 6H), 3.68 (s, 2H), 3.58 (s, 2H), 3.47 (t, *J* = 5.4 Hz, 2H), 2.44 (s, 3H); ^{13}C NMR (DMSO-d_6, 60 °C, 126 MHz) δ 170.0, 167.7, 164.1, 154.5, 153.0, 141.3, 139.8, 136.6, 135.3, 133.1, 128.1, 125.9, 121.3, 119.0, 108.8, 106.6, 71.1, 56.1, 48.5, 42.3, 41.8, 32.1, 22.5; ESI-HRMS: *m/z* calcd for $C_{26}H_{32}N_6O_6S_2$ $[M+H]^+$: 589.1898. Found: 589.1899.

Synthesis of *m*BnA-OMe -TMP(gly$_1$) (**5**)

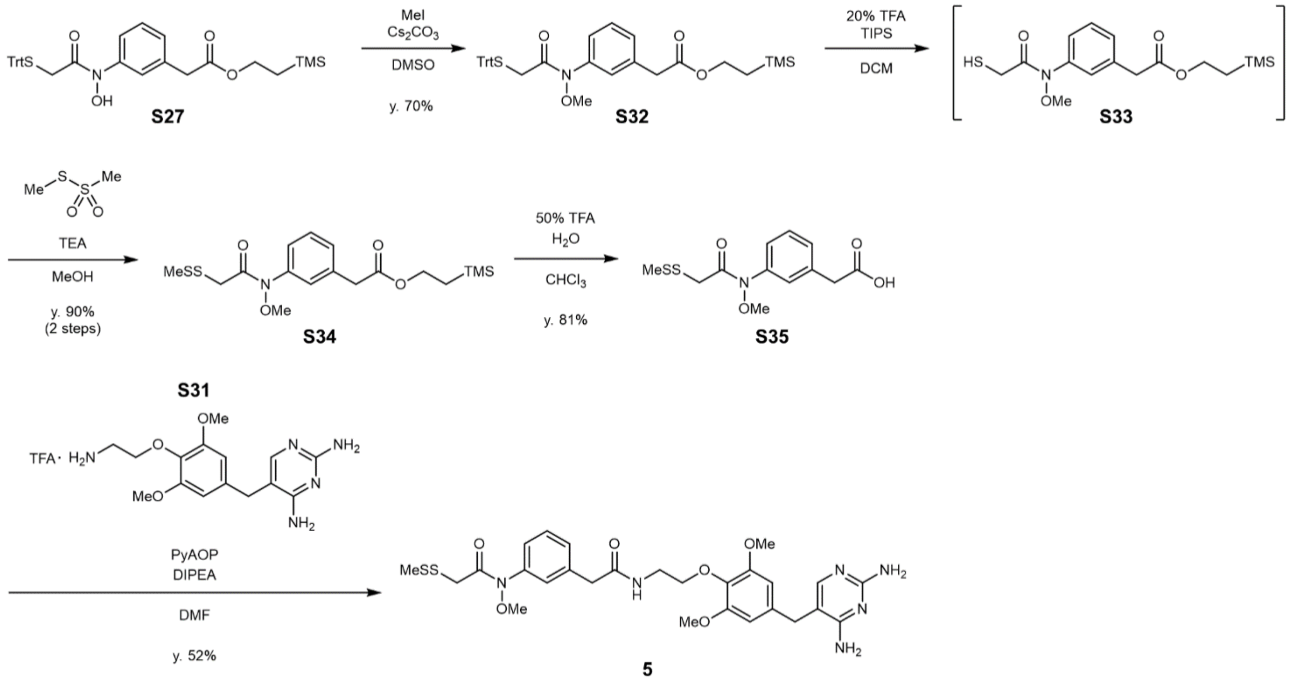

2-(Trimethylsilyl)ethyl 2-(3-(*N*-methoxy-2-(tritylthio)acetamido)phenyl)acetate (S32):

To a stirred solution of **S27** (200 mg, 0.343 mmol) in DMSO (6.85 mL), iodomethane (64.0 μL, 1.03 mmol) and Cs_2CO_3 (167 mg, 0.514 mmol) were added, and the mixture was stirred at r.t. for 30 min. To the reaction mixture, saturated NH_4Cl aq. was added, and the aqueous layer was extracted twice with EtOAc. Combined organic layers were washed with saturated NaCl aq., dried over Na_2SO_4, filtered, and concentrated to afford crude **S32**, which was purified with silica gel column chromatography (5 to 30% EtOAc/hexane) to afford **S32** (144 mg, 0.240 mmol, y. 70%) as pale orange amorphous solid.

^{1}H NMR (DMSO-d_6, 60 °C, 500 MHz) δ 7.36-7.29 (m, 14H), 7.26-7.22 (m, 4H), 7.18 (d, *J* = 7.4 Hz, 1H), 4.15 (t, *J* = 8.3 Hz, 2H), 3.65 (s, 2H), 3.47 (s, 3H), 3.16 (s, 2H), 0.95 (t, *J* = 8.3 Hz, 2H), 0.00 (s, 9H); ^{13}C NMR (DMSO-d_6, 60 °C, 126 MHz) δ 170.3, 166.7, 143.7, 137.4, 135.1, 128.8, 128.5, 127.7, 127.5, 126.5, 123.5, 121.4, 66.4, 62.0, 61.4, 40.1, 34.5, 16.6, -1.87; ESI-HRMS: *m/z* calcd for $C_{35}H_{39}NO_4SSi$ $[M+Na]^+$: 620.2261. Found: 620.2254.

2-(Trimethylsilyl)ethyl 2-(3-(2-mercapto-*N*-methoxyacetamido)phenyl)acetate (S33):

To a stirred solution of **S32** (110 mg, 0.184 mmol) in DCM (2.94 mL), TIPS (113 μL, 0.552 mmol) and TFA (736 μL) were added, and the mixture was stirred at r.t. for 10 min. The mixture was concentrated. The residue was roughly purified with silica gel column chromatography (25% EtOAc/hexane) to afford crude **S33** (86.4 mg), which was used in the next step immediately without further purification.

2-(Trimethylsilyl)ethyl 2-(3-(*N*-methoxy-2-(methylsulfinothioyl)acetamido)phenyl)acetate (S34):

To a stirred solution of **S33** (86.4 mg) in MeOH (3.68 mL), *S*-methyl methanesulfonothioate (17.2 μL, 0.184 mmol) and TEA (30.8 μL, 0.221 mmol) were added, and the mixture was stirred at r.t. for 1 h. To the reaction mixture, water was added, and the aqueous layer was extracted twice with EtOAc. Combined organic layers were washed with saturated NaCl aq., dried over Na_2SO_4, filtered, and concentrated to afford crude **S34**, which was purified with silica gel column chromatography (17% EtOAc/hexane) to afford **S34** (61.7 mg, 0.165 mmol, y. 90% for 2 steps) as pale orange oil (*note*: a ca. 1:1 mixture of *cis-trans* isomers at hydroxamic acid (major isomer A and minor isomer B)).

^{1}H NMR (DMSO-d_6, 60 °C, 500 MHz) δ 7.41 (s, 1H), 7.37 (br, 2H), 7.19 (br, 1H), 4.18-4.16 (m, 4H), 4.03 (s, 2H, isomer B), 3.90 (s, 2H, isomer A), 3.72 (s, 3H, isomer A), 3.69 (s, 3H, isomer B), 3.68 (s, 2H, isomer A), 3.66 (s, 2H, isomer B), 2.46 (s, 3H, isomer A), 2.41 (s, 3H, isomer B), 0.95 (t, *J* = 8.0 Hz, 2H), 0.01 (s, 9H); ^{13}C NMR (DMSO-d_6, 60 °C, 126 MHz) δ 170.3, 167.2 (isomer A), 167.0 (isomer B), 137.6 (isomer B), 137.5 (isomer A), 135.2 (isomer A), 135.1 (isomer B), 128.5 (isomer A), 128.5 (isomer B), 127.4, 123.3, 121.3, 62.0, 61.7 (isomer B), 61.7 (isomer A), 41.4, 40.6 (isomer B), 40.1 (isomer A), 22.3 (isomer A), 21.2 (isomer B), 16.6, -1.87; ESI-HRMS: *m/z* calcd for $C_{17}H_{27}NO_4S_2Si$ [M+Na]$^+$: 424.1043. Found: 424.1045.

2-(3-(*N*-Methoxy-2-(methylsulfinothioyl)acetamido)phenyl)acetic acid (S35):

A solution of **S34** (35.0 mg, 0.0871 mmol) in $CHCl_3$ (863 μL), water (17.3 μL) and TFA (863 μL) was stirred at 50 °C for 40 min. The mixture was concentrated to afford crude **S35**, which was purified with preparative HPLC (20-100% MeCN in 0.1% TFA/H_2O) to afford **S35** (21.3 mg, 0.0707 mmol, y. 81%) as pale yellow oil (*note*: a ca. 2:1 mixture of *cis-trans* isomers at hydroxamic acid (major isomer A and minor isomer B)).

^{1}H NMR (DMSO-d_6, 60 °C, 500 MHz) δ 7.49 (s, 1H), 7.46 (m, 2H), 7.29 (d, *J* = 5.7 Hz, 1H), 4.10 (s, 2H, isomer B), 3.98 (s, 2H, isomer A), 3.80 (s, 3H, isomer A), 3.77 (s, 3H, isomer B), 3.70 (s, 2H, isomer A), 3.69 (s, 2H, isomer B), 2.54 (s, 3H); ^{13}C NMR (DMSO-d_6, 60 °C, 126 MHz) δ 171.9, 167.4, 137.7, 135.9, 128.6, 127.7, 123.7, 121.4, 61.9, 41.5 (isomer B), 40.7 (isomer A), 40.4, 22.5; ESI-HRMS: *m/z* calcd for $C_{12}H_{15}NO_4S_2$ [M+Na]$^+$: 324.0335. Found: 324.0338.

***N*-(3-(2-((2-(4-((2,4-Diaminopyrimidin-5-yl)methyl)-2,6-dimethoxyphenoxy)ethyl)amino)-2-oxoethyl)phenyl)-*N*-methoxy-2-(methylsulfinothioyl)acetamide (5):**

To a stirred solution of **S35** (11.0 mg, 0.0365 mmol) in DMF (730 μL), **S31** (17.4 mg, 0.0401 mmol), PyAOP (47.6 mg, 0.0912 mmol) and DIPEA (31.8 μL, 0.182 mmol) were added, and the mixture was stirred at r.t. for 1 h. The mixture was concentrated to afford crude **5**, which was purified with preparative HPLC (20-100% MeCN in 0.1% TFA/H_2O) to afford **5** (11.4 mg, 0.0189 mmol, y. 52%) as pale pink solid (*note*: a ca. 3:1 mixture of *cis-trans* isomers at hydroxamic acid (major isomer A and minor isomer B)).

^{1}H NMR (CD_3OD, 60 °C, 500 MHz) δ 7.45 (s, 1H), 7.42-7.37 (m, 2H), 7.29 (d, *J* = 6.3 Hz, 1H), 7.26 (s, 1H), 6.56 (s, 2H), 4.00 (t, *J* = 5.3 Hz, 2H), 3.78 (br, 8H), 3.73 (s, 3H), 3.68 (s, 2H), 3.61 (s, 2H), 3.48 (t, *J* = 5.3 Hz, 2H), 2.44 (s, 3H); ^{13}C NMR (DMSO-d_6, 60 °C, 126 MHz) δ 170.0, 169.6, 164.0, 154.4, 152.8, 139.8, 137.5, 137.2, 135.3, 133.0, 128.5, 127.4, 123.5, 121.2, 108.7, 106.5, 71.0, 61.8 (isomer A), 59.5

(isomer B), 56.0, 42.0, 40.6, 31.9, 22.4 (isomer A), 20.4 (isomer B), 13.8; ESI-HRMS: *m/z* calcd for $C_{27}H_{34}N_6O_6S_2$ $[M+H]^+$: 603.2054. Found: 603.2053.

Synthesis of *m*BnA-SMe-TMP(gly$_1$) (**6**)

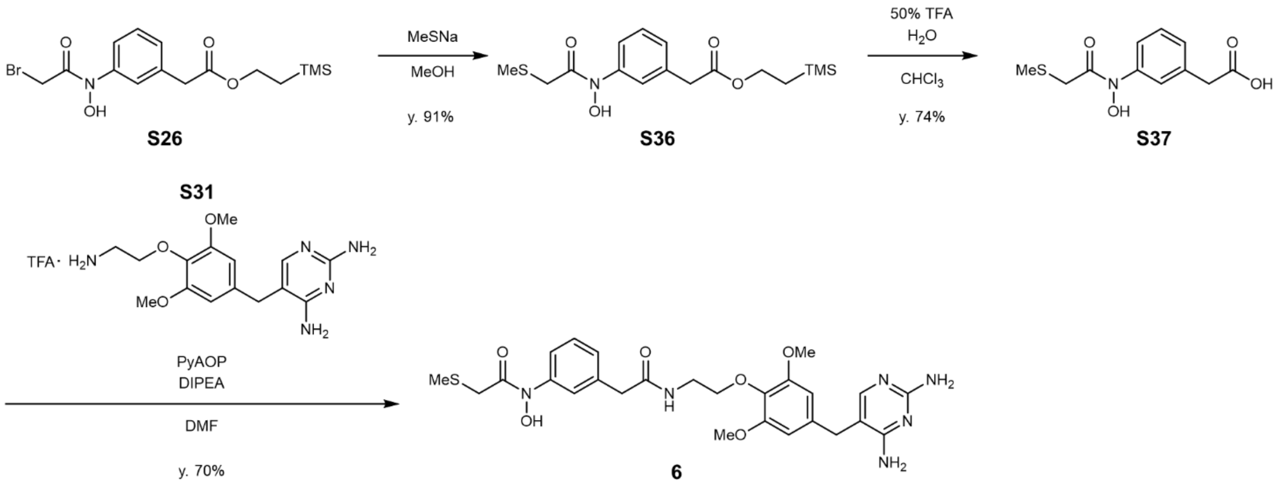

2-(Trimethylsilyl)ethyl 2-(3-(*N*-hydroxy-2-(methylthio)acetamido)phenyl)acetate (S36):

To a stirred solution of **S26** (146 mg, 0.377 mmol) in MeOH (2.26 mL), sodium methanethiolate (31.7 mg, 0.452 mmol) was added, and the mixture was stirred at r.t. for 4.5 h. To the reaction mixture, saturated NH_4Cl aq. was added, and the aqueous layer was extracted twice with DCM. Combined organic layers were washed with saturated NaCl aq., dried over Na_2SO_4, filtered, and concentrated to afford pure **S36** (122 mg, 0.343 mmol, y. 91%) as yellow oil.

^{1}H NMR (DMSO-d_6, 60 °C, 500 MHz) δ 10.55 (brs, 1H), 7.57 (s, 1H), 7.54 (d, *J* = 8.3 Hz, 1H), 7.31 (dd, *J* = 8.3, 7.7 Hz, 1H), 7.07 (d, *J* = 7.7 Hz, 1H), 4.16 (t, *J* = 8.2 Hz, 2H), 3.64 (s, 2H), 3.57 (s, 2H), 2.18 (s, 3H), 0.96 (t, *J* = 8.2 Hz, 2H), 0.02 (s, 9H); ^{13}C NMR (DMSO-d_6, 60 °C, 126 MHz) δ 170.5, 168.2, 141.5, 134.5, 128.0, 125.6, 121.2, 119.0, 62.0, 40.4, 35.4, 16.7, 15.2, -1.81; ESI-HRMS: *m/z* calcd for $C_{16}H_{25}NO_4SSi$ $[M+Na]^+$: 378.1166. Found: 378.1172.

2-(3-(*N*-Hydroxy-2-(methylthio)acetamido)phenyl)acetic acid (S37):

A solution of **S36** (100 mg, 0.281 mmol) in $CHCl_3$ (2.78 mL), water (55.7 μL) and TFA (2.78 mL) was stirred at 50 °C for 30 min. The mixture was concentrated to afford crude **S37**, which was purified with preparative HPLC (20-100% MeCN in 0.1% TFA/H_2O) to afford **S37** (52.8 mg, 0.207 mmol, y. 74%) as orange oil.

^{1}H NMR (DMSO-d_6, 60 °C, 500 MHz) δ 7.56 (s, 1H), 7.52 (d, *J* = 8.0 Hz, 1H), 7.31 (dd, *J* = 8.0, 7.4 Hz, 1H), 7.08 (d, *J* = 7.4 Hz, 1H), 3.58 (s, 2H), 3.57 (s, 2H), 2.18 (s, 3H); ^{13}C NMR (DMSO-d_6, 60 °C, 126 MHz) δ 172.2, 168.4, 141.5, 135.3, 128.2, 126.0, 121.6, 119.2, 40.7, 35.6, 15.4; ESI-HRMS: *m/z* calcd for $C_{11}H_{13}NO_4S$ $[M+Na]^+$: 278.0457. Found: 278.0460.

***N*-(3-(2-((2-(4-((2,4-Diaminopyrimidin-5-yl)methyl)-2,6-dimethoxyphenoxy)ethyl)amino)-2-oxoethyl)phenyl)-*N*-hydroxy-2-(methylthio)acetamide (6):**

To a stirred solution of **S37** (11.8 mg, 0.0462 mmol) in DMF (924 μL), **S31** (22.0 mg, 0.0508 mmol), PyAOP (60.2 mg, 0.116 mmol) and DIPEA (40.3 μL, 0.231 mmol) were added, and the mixture was stirred at r.t.

for 1 h. The mixture was concentrated to afford crude **6**, which was purified with preparative HPLC (20-100% MeCN in 0.1% TFA/H_2O) to afford **6** (17.9 mg, 0.0322 mmol, y. 70%) as orange solid.

^{1}H NMR (CD_3OD, 60 °C, 500 MHz) δ 7.56 (s, 1H), 7.50 (d, *J* = 7.7 Hz, 1H), 7.34 (dd, *J* = 7.7, 7.4 Hz, 1H), 7.26 (s, 1H), 7.19 (d, *J* = 7.4 Hz, 1H), 6.56 (s, 2H), 4.01 (t, *J* = 5.4 Hz, 2H), 3.78 (s, 6H), 3.68 (s, 2H), 3.58 (s, 2H), 3.52 (brs, 2H), 3.47 (t, *J* = 5.4 Hz, 2H), 2.20 (s, 3H); ^{13}C NMR (DMSO-d_6, 60 °C, 126 MHz) δ 169.9, 168.3, 164.1, 154.4, 152.9, 141.4, 139.8, 136.5, 135.3, 133.0, 128.0, 125.7, 121.4, 119.0, 108.7, 106.5, 71.0, 56.0, 48.4, 42.2, 35.6, 32.0, 15.3; ESI-HRMS: *m/z* calcd for $C_{26}H_{32}N_6O_6S$ [M+H]$^+$: 557.2177. Found: 557.2174.

Synthesis of *p*HXA-SMe-Me(gly$_3$) (**S1**)

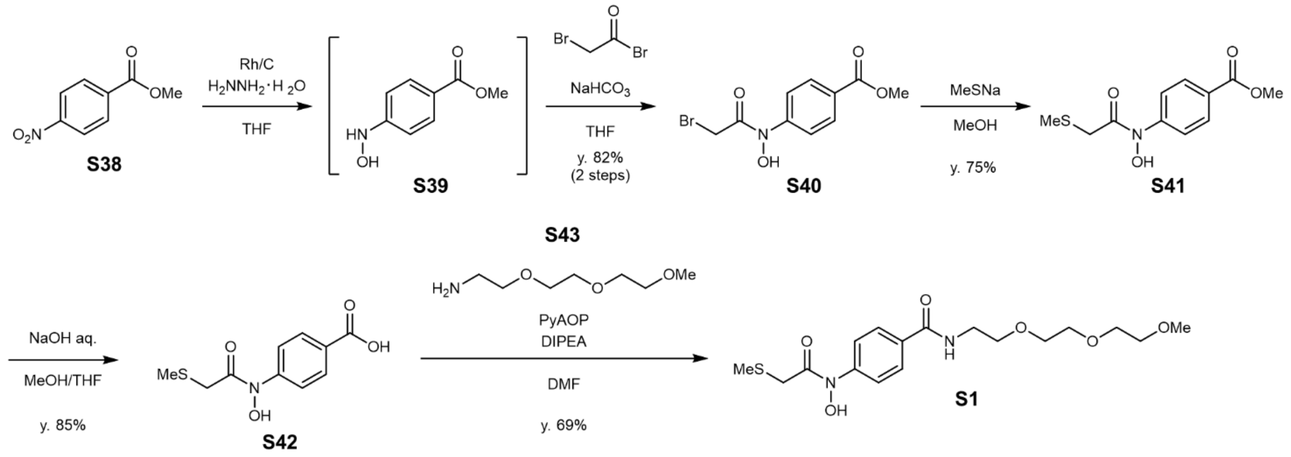

Methyl 4-(hydroxyamino)benzoate (S39):

To a stirred solution of **S38** (600 mg, 3.31 mmol) in THF (16.6 mL), rhodium 5% on carbon (177 mg, 0.0387 mmol) was added and then cooled to 0 °C. To the mixture, hydrazine monohydrate (386 μL, 7.95 mmol) was added, and the mixture was stirred at 0 °C for 1 h. The reaction mixture was filtered through Celite to afford crude **S39** solution in ca. 45 mL THF, which was used in the next step immediately without further purification.

Methyl 4-(2-bromo-*N*-hydroxyacetamido)benzoate (S40):

To a stirred solution of crude **S39** in ca. 45 mL THF at 0 °C, $NaHCO_3$ (2.78 g, 33.1 mmol) was added, and the mixture was stirred for 30 min. 2-Bromoacetyl bromide (1.43 mL, 16.6 mmol) in THF (5.00 mL) was added dropwise, and the mixture was stirred at 0 °C for 1 h. To the reaction mixture, water was added, and most of the THF was removed under reduced pressure. The mixture was extracted twice with EtOAc. Combined organic layers were washed with saturated NaCl aq., dried over Na_2SO_4, filtered, and concentrated to afford crude **S40**, which was washed with 10% EtOAc/hexane to afford **S40** (781 mg, 2.71 mmol, y. 82% for 2 steps) as white solid.

^{1}H NMR ($CDCl_3$/CD_3OD=1/2, 500 MHz) δ 7.90 (d, J = 8.6 Hz, 2H), 7.71 (d, J = 8.6 Hz, 2H), 4.18 (s, 2H), 3.79 (s, 3H); ^{13}C NMR ($CDCl_3$/CD_3OD=1/2, 126 MHz) δ 174.2, 166.7, 144.6, 130.0, 126.2, 119.0, 52.0, 28.2; ESI-HRMS: *m/z* calcd for $C_{10}H_{10}BrNO_4$ $[M+Na]^+$: 309.9685. Found: 309.9688.

Methyl 4-(*N*-hydroxy-2-(methylthio)acetamido)benzoate (S41):

To a stirred solution of **S40** (420 mg, 1.46 mmol) in MeOH (8.73 mL), sodium methanethiolate (123 mg, 1.75 mmol) was added, and the mixture was stirred at r.t. for 1.5 h. To the reaction mixture, saturated NH_4Cl aq. was added, and the aqueous layer was extracted twice with DCM. Combined organic layers were washed with saturated NaCl aq., dried over Na_2SO_4, filtered, and concentrated to afford crude **S41**,

which was purified with silica gel column chromatography (20 to 50% EtOAc/hexane) to afford **S41** (278 mg, 1.09 mmol, y. 75%) as white solid.

^{1}H NMR ($CDCl_3$, 392 MHz) δ 9.09 (s, 1H), 7.78 (d, *J* = 7.6 Hz, 2H), 7.55 (d, *J* = 7.6 Hz, 2H), 3.76 (s, 3H), 3.46 (brs, 2H), 2.01 (s, 3H); ^{13}C NMR ($CDCl_3$, 99 MHz) δ 170.0, 166.5, 144.2, 129.8, 125.9, 119.2, 52.0, 36.3, 15.7; ESI-HRMS: *m/z* calcd for $C_{11}H_{13}NO_4S$ $[M+Na]^+$: 278.0457. Found: 278.0462.

4-(*N*-Hydroxy-2-(methylthio)acetamido)benzoic acid (S42):

To a stirred solution of **S41** (174 mg, 0.682 mmol) in MeOH (6.82 mL) and THF (6.82 mL), 1 N NaOH aq. (6.82 mL) was added, and the mixture was stirred at r.t. for 2.5 h. After completion of the reaction, the mixture was cooled to 0 °C, and pH was adjusted to 4 with 1 N HCl aq. The mixture was concentrated, the residue was dissolved in 20% MeOH/DCM, and then filtered off. The filtrate was concentrated to afford pure **S42** (140 mg, 0.580 mmol, y. 85%) as pale yellow solid (*note*: a ca. 10:1 mixture of *cis-trans* isomers at hydroxamic acid (major isomer A and minor isomer B)).

^{1}H NMR (CD_3OD, 60 °C, 500 MHz) δ 8.22 (d, *J* = 8.6 Hz, 2H, isomer B), 8.14 (d, *J* = 8.6 Hz, 2H, isomer B), 8.01 (d, *J* = 8.6 Hz, 2H, isomer A), 7.77 (d, *J* = 8.6 Hz, 2H, isomer A), 3.62 (s, 2H, isomer A), 3.17 (s, 2H, isomer B), 2.22 (s, 3H, isomer A), 2.16 (s, 3H, isomer B); ^{13}C NMR (CD_3OD, 60 °C, 126 MHz) δ 171.7, 169.9, 146.3, 131.2, 124.1, 121.2, 37.5 (isomer B), 37.1 (isomer A), 16.1; ESI-HRMS: *m/z* calcd for $C_{10}H_{11}NO_4S$ $[M+Na]^+$: 264.0301. Found: 264.0306.

4-(*N*-Methoxy-2-(methylthio)acetamido)-*N*-(2-(2-(2-methoxyethoxy)ethoxy)ethyl)benzamide (S1):

To a stirred solution of **S42** (40.0 mg, 0.166 mmol) in DMF (3.32 mL), **S43**[6] (54.1 mg, 0.332 mmol), PyAOP (173 mg, 0.332 mmol) and DIPEA (144 μL, 0.829 mmol) were added, and the mixture was stirred at r.t. for 1 h. The mixture was concentrated to afford crude **S1**, which was purified with preparative HPLC (20-100% MeCN in 0.1% TFA/H_2O) to afford **S1** (44.1 mg, 0.114 mmol, y. 69%) as orange syrup.

^{1}H NMR (CD_3OD, 392 MHz) δ 7.85 (d, *J* = 8.3 Hz, 2H), 7.79 (d, *J* = 8.3 Hz, 2H), 3.64-3.60 (m, 13H), 3.56 (t, *J* = 5.2 Hz, 2H), 3.50 (t, *J* = 4.5 Hz, 2H), 2.22 (s, 3H); ^{13}C NMR (CD_3OD, 99 MHz) δ 171.6, 169.4, 145.4, 131.8, 128.8, 120.9, 72.8, 71.5, 71.3, 71.2, 70.5, 59.1, 40.9, 37.1, 16.1; ESI-HRMS: *m/z* calcd for $C_{17}H_{26}N_2O_6S$ $[M+Na]^+$: 409.1404. Found: 409.1402.

Synthesis of *m*BnA-SMe-Me(gly$_3$) (**S2**)

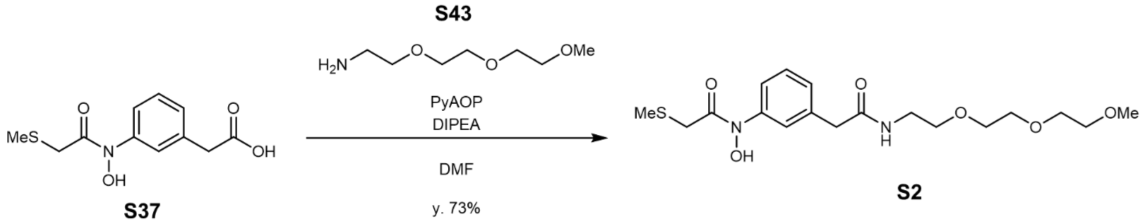

***N*-Methoxy-2-(methylthio)-*N*-(3-(12-oxo-2,5,8-trioxa-11-azatridecan-13-yl)phenyl)acetamide (S2):**
To a stirred solution of **S37** (17.2 mg, 0.0674 mmol) in DMF (1.35 mL), **S43** (22.0 mg, 0.135 mmol), PyAOP (70.3 mg, 0.135 mmol) and DIPEA (58.7 μL, 0.337 mmol) were added, and the mixture was stirred at r.t. for 50 min. The mixture was concentrated to afford crude **S2**, which was purified with preparative HPLC (20-100% MeCN in 0.1% TFA/H_2O) to afford **S2** (19.6 mg, 0.0489 mmol, y. 73%) as yellow oil.
^{1}H NMR (CD_3OD, 60 °C, 392 MHz) δ 7.51 (s, 1H), 7.46 (d, *J* = 8.1 Hz, 1H), 7.29 (dd, *J* = 8.1, 7.6 Hz, 1H), 7.14 (d, *J* = 7.6 Hz, 1H), 3.58-3.55 (m, 7H), 3.50-3.48 (m, 8H), 3.33 (t, *J* = 5.4 Hz, 2H), 3.27 (s, 2H), 2.18 (s, 3H); ^{13}C NMR (DMSO-d_6, 60 °C, 126 MHz) δ 169.8, 168.3, 141.4,136.6, 127.9, 125.7, 121.3, 118.9, 71.2, 69.6, 69.5, 69.5, 68.9, 57.9, 42.2, 38.7, 35.5, 15.3; ESI-HRMS: *m/z* calcd for $C_{18}H_{28}N_2O_6S$ [M+Na]$^+$: 423.1560. Found: 423.1568.

Synthesis of YZ-TMP(gly$_2$) (**S5**)

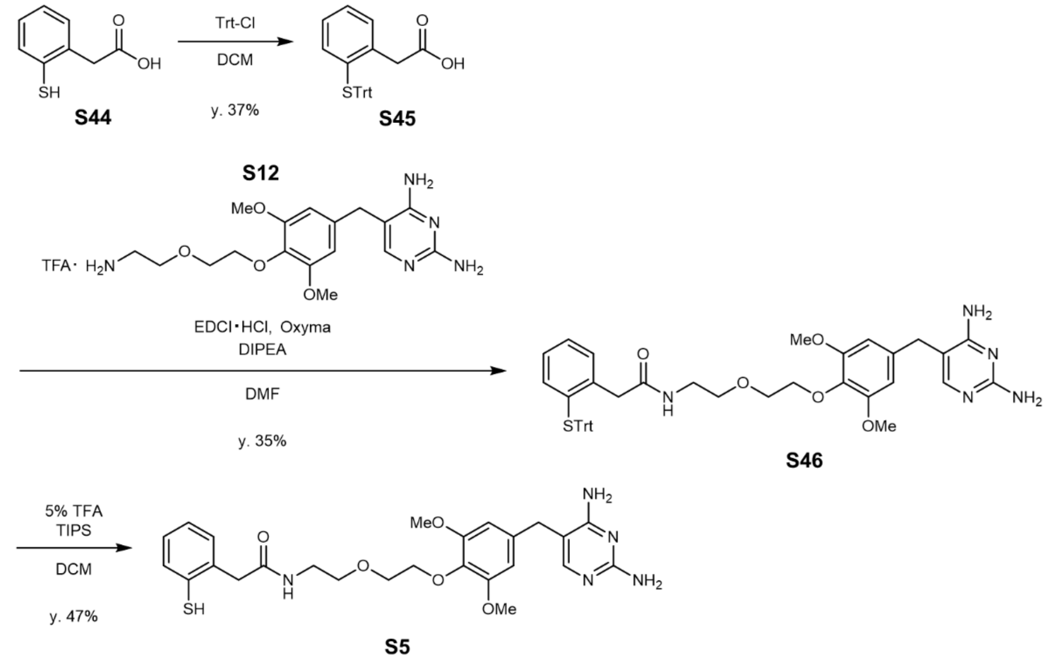

2-(2-(Tritylthio)phenyl)acetic acid (S45):

To a stirred solution of **S44**[4] (200 mg, 1.19 mmol) in DCM (1.20 mL), trityl chloride (331 mg, 1.19 mmol) was added, and the mixture was stirred at r.t. for 3 h. The reaction mixture was quenched by 1 N NaOH aq., and pH was adjusted to 4 with 1 N HCl aq. The aqueous layer was extracted twice with EtOAc. Combined organic layers were washed with saturated NaCl aq., dried over Na_2SO_4, filtered, and concentrated to afford crude **S45**, which was purified with silica gel column chromatography (10 to 30% EtOAc/hexane) to afford **S45** (178 mg, 0.434 mmol, y. 37%) as pale brown solid.

^{1}H NMR ($CDCl_3$, 500 MHz) δ 7.34 (dd, *J* = 3.4 Hz, 6H), 7.25-7.18 (m, 11H), 7.11 (d, *J* = 7.4 Hz, 1H), 6.98 (t, *J* = 7.4 Hz, 1H), 3.22 (s, 2H); ^{13}C NMR ($CDCl_3$, 126 MHz) δ 176.8, 144.0, 139.3, 136.1, 134.0, 130.3, 130.0, 128.8, 127.6, 127.3, 126.8, 71.3, 38.7; ESI-HRMS: *m/z* calcd for $C_{27}H_{22}O_2S$ [M+Na]$^+$: 433.1233. Found: 433.1236.

***N*-(2-(2-(4-((2,4-Diaminopyrimidin-5-yl)methyl)-2,6-dimethoxyphenoxy)ethoxy)ethyl)-2-(2-(tritylthio)phenyl)acetamide (S46):**

To a stirred solution of **S45** (20.0 mg, 0.0487 mmol) in DMF (974 μL), **S12** (25.6 mg, 0.0536 mmol), EDCI·HCl (18.7 mg, 0.0974 mmol), Oxyma (13.8 mg, 0.0974 mmol), DIPEA (33.9 μL, 0.195 mmol) were added, and the mixture was stirred at r.t. for 2 h. To the reaction mixture, water was added, and the aqueous layer was extracted twice with EtOAc. Combined organic layers were washed with saturated NaCl aq., dried over Na_2SO_4, filtered, and concentrated to afford crude **S46**, which was purified with silica gel column chromatography (0 to 10% MeOH/DCM) and then preparative TLC (0.25 mm plates, MeOH/DCM = 1/10) to afford **S46** (12.8 mg, 0.0169 mmol, 35%) as white solid.

^{1}H NMR ($CDCl_3$, 392 MHz) δ 7.81 (s, 1H), 7.33-7.30 (m, 8H), 7.23 (d, *J* = 3.1 Hz, 6H), 7.16 (t, *J* = 5.4 Hz, 3H), 6.93 (dd, *J* = 7.6, 4.5 Hz, 1H), 6.40 (s, 2H), 5.92 (br, 1H), 4.80 (brs, 2H), 4.61 (brs, 2H), 4.09 (t, *J* = 5.1 Hz, 2H), 6.10 (s, 6H), 3.72 (t, *J* = 4.8 Hz, 2H), 3.68 (s, 2H), 3.57 (t, *J* = 4.8 Hz, 2H), 3.42 (t, *J* = 5.1 Hz, 2H), 3.12 (s, 2H); ^{13}C NMR ($CDCl_3$, 126 MHz) δ 170.8, 162.7, 162.1, 156.7, 153.6, 144.0, 140.4, 135.8, 135.6, 133.9, 133.8, 130.3, 130.0, 128.8, 127.6, 127.0, 126.8, 106.3, 104.9, 72.1, 71.3, 70.2, 69.7, 56.1, 41.4, 39.3, 34.7; ESI-HRMS: *m/z* calcd for $C_{44}H_{45}N_5O_5S$ $[M+H]^+$: 756.3214. Found: 754.3220.

***N*-(2-(2-(4-((2,4-Diaminopyrimidin-5-yl)methyl)-2,6-dimethoxyphenoxy)ethoxy)ethyl)-2-(2-mercaptophenyl)acetamide (S5):**

To a stirred solution of **S46** (14.0 mg, 0.0185 mmol) in DCM (342 μL), TIPS (11.4 μL, 0.0556 mmol) and TFA (18.0 μL) were added, and the mixture was stirred at r.t. for 1 h. To the reaction mixture, saturated $NaHCO_3$ was added, and pH was adjusted to 4 by 1 N HCl. The aqueous layer was extracted twice with 5% MeOH/DCM followed by twice with 20% MeOH/DCM. Combined organic layers were washed with saturated NaCl aq., dried over Na_2SO_4, filtered, and concentrated to afford crude **S5**, which was purified with silica gel column chromatography (1st: 0 to 33% MeOH/DCM, 2nd: 5 to 33% MeOH/DCM) to afford **S5** (4.5 mg, 0.019 mmol, y. 47%) as white solid.

^{1}H NMR (CD_3OD, 392 MHz) δ 7.35 (dd, *J* = 5.4, 3.6 Hz, 1H), 7.24-7.21 (m, 2H), 7.10 (d, *J* = 3.6 Hz, 1H), 7.09 (d, *J* = 3.1 Hz, 1H), 6.57 (s, 2H), 4.05 (t, *J* = 4.5 Hz, 2H), 3.81 (s, 6H), 3.72 (t, *J* = 4.5 Hz, 2H), 3.66 (s, 2H), 3.65 (s, 2H), 3.60 (t, *J* = 5.4 Hz, 2H), 3.40 (t, *J* = 5.4 Hz, 2H); ^{13}C NMR (CD_3OD, 126 MHz) δ 173.1, 165.9, 157.4, 154.9, 143.1, 137.7, 137.6, 137.0, 134.4, 132.9, 132.1, 129.8, 129.1, 110.5, 107.2, 73.5, 71.3, 70.4, 56.7, 41.6, 40.7, 34.0; ESI-HRMS: *m/z* calcd for $C_{50}H_{60}N_{10}O_{10}S_2$ $[M+2H]^{2+}$: 513.2040. Found: 513.2036 (*note*: detected as a disulfide dimer).

Chemical experiments

Determination of pK_a values of the catalysts

An appropriate amount of NaOH (3 M) or HCl (3 M) was added to Tris Buffer (50 mM Tris-HCl (pH 7.5), 100 mM NaCl) to basify or acidify the solution, respectively. To the pH-adjusted solution, **S1** or **S2** (final 500 μM) was added, the pH value was measured, and the solution was analyzed with UV-Vis spectrophotometer.

Biochemical experiments

In-cell acetylation of histone with p300/CBP siRNA treatment

The 12 well plate was pre-treated with poly-D-lysine, washed with PBS, and then air dried. 2.8 x 10^5 HEK293T cells in 1 mL DMEM++ were seeded into wells of a 12-well plate with the transfection of control siRNA-A (SantaCruz, sc-37007, 24 pmol) or p300 siRNA (SantaCruz, sc-29431, 12 pmol) and CBP siRNA (SantaCruz, sc-29244, 12 pmol) using Lipofectamine RNAiMax transfection reagent (Invitrogen, 13778075) according to the manufacturer’s instructions (Reverse transfection). After 24 h, the medium was carefully removed and replaced with 1 mL DMEM++. The cells were transfected with pcDNA5/TO-LieD@R52-FLAG plasmid (1.25 μg per well) using Lipofectamine LTX and Plus reagents according to the manufacturer’s instructions. After 8 h, the medium was carefully removed and replaced with 1 mL DMEM+++ supplemented BSO (100 μM). After 16 h, the medium was carefully removed and replaced with 0.4 mL Opti-MEM supplemented with catalyst-TMP (100 μM), BSO (100 μM) and 0.5% DMSO. After 10 h, the cells were detached by pipetting vigorously, transferred to an Eppendorf tube, centrifuged (304 g, 5 min, 4 °C), and washed with PBS. For immunoblotting of whole cell extracts, the cells were lysed with CRB++++ buffer on ice for 30 min. After centrifugation (21,130 g, 20 min), the supernatant was used for western blotting with indicated antibodies or SDS-PAGE followed by Oriole staining.

In-cell acylation of histone with *m*BnA(gly$_1$)-TMP **4** and overexpressed p300

2.8 x 10^5 HEK293T cells in 1 mL DMEM++ were seeded into wells of a 12-well plate. After 24 h incubation, the cells were transfected with pcDNA3.1-p300 plasmid (Addgene, #23252, 1.25 μg per well), pcDNA5/TO-LieD@R52-FLAG (1.25 μg per well), or pcDNA3 empty vector (1.25 μg per well) using Lipofectamine LTX and Plus reagents according to the manufacturer’s instructions. After 12 h, the medium was carefully removed and replaced with 1 mL DMEM+++ supplemented with BSO (100 μM). After 24 h, the medium was carefully removed and replaced with 0.4 mL Opti-MEM supplemented with BSO (100 μM) and 0.5% DMSO for p300-overexpressed cells and control cells, or supplemented with catalyst-TMP (100 μM), BSO (100 μM) and 0.5% DMSO. After 10 h, the cells were detached by pipetting vigorously, transferred to an Eppendorf tube, centrifuged (304 g, 5 min, 4 °C), and washed with PBS. For immunoblotting of whole-cell extracts, the cells were lysed with CRB+++++ buffer on ice for 30 min, and then treated as described in “In-cell acetylation of histone with p300/CBP siRNA treatment”.

ChIP-assay

HEK293T cells were treated as described in “In-cell acetylation of histone using endogenous Ac-CoA”. After acetylation reaction, the medium was replaced with DMEM+++. To fix the cells, 27 μL of 37% formaldehyde was added per 1 mL medium. After incubation at r.t. for 15 min, 80 μL of 1.25 M glycine in PBS was added per 1 mL medium to quench the fixation reaction, and the cells were harvested and washed with PBS twice. The cells were suspended in SDS lysis buffer (50 mM Tris-HCl (pH 8.1), 1% SDS, 10 mM EDTA, protease inhibitor cocktail), incubated on ice for 10 min, and sonicated with BioRaptor® II (BM Equipment Co). The supernatants were diluted 10-fold with ChIP dilution buffer (16.7 mM Tris-HCl (pH 8.1) 0.01% SDS, 1.1% Triton X-100, 1.2 mM EDTA, 167 mM NaCl), determined the DNA concentrations by Nanodrop® lite (Thermo). For H2BK120 and normal rabbit IgG immunoprecipitations, 500 μL of extract (100 ng/μL for DNA) was incubated with 1 μg antibodies for 30 min on ice. For H2B and normal rabbit IgG immunoprecipitations, 100 μL of extract was incubated with 1 mg antibodies for 30 min on ice. After adding Dynabeads® Protein G (20 μL, Veritas), the extract-antibody mixtures were incubated at 4 °C for 2 h with rotating. After washing in LS buffer (20 mM Tris-HCl (pH 8.1), 150 mM NaCl, 0.1% SDS, 1% Triton X-100, 2 mM EDTA), HS buffer (20 mM Tris-HCl (pH 8.1), 500 mM NaCl, 0.1% SDS, 1% Triton X-100, 2 mM EDTA), LiCl buffer (10 mM Tris-HCl (pH 8.1), 0.25 M LiCl, 1% NP-40, 1% sodium deoxycholate, 1 mM EDTA), and TE buffer (10 mM Tris-HCl (pH 8), 1 mM EDTA), the co-immunoprecipitated DNA was extracted at r.t. for 15 min with elution buffer (100 mM $NaHCO_3$, 1% SDS, 10 mM DTT). The supernatant was collected, incubated at 65 °C overnight, and then treated with Proteinase K (Takara, 9034) at 45 °C for 1 h. For input sample, 100 μL of extract (100 ng/μL for DNA) was incubated with 200 mM NaCl at 65 °C overnight, and treated with Proteinase K at 45 °C for 1 h. DNA was purified by PCR Clean-Up Mini Kit (Favorgen) before performing PCR. Purified DNA was analyzed by quantitative PCR using LightCycler® 480 system (Roche) with SYBR™ Green I Master (Roche). The amount of co-immunoprecipitated DNA was divided by the amount of total DNA of input to calculate %input of each antibody. Primers used in ChIP assays are listed in **Supplementary Table 3**.

Profiling of the reactivity of lysine residues using STP ester probe

Recombinant AR (0.35 μM) or nucleosomes (0.175 μM, 0.35 μM as H2BK120) was treated with STP ester probe (1 mM) at r.t. in Tris buffer for 1 h. Proteins in the reaction mixture were precipitated by trichloroacetic acid (TCA, 16.6%). The protein was collected by centrifugation, air-dried, and dissolved in MQ. After DNA was digested by DNase I (Takara, 2270A) for 30 min at 37 °C, the samples were mixed with acetone (74%) and incubated overnight at −30 °C, then the proteins were collected by centrifugation, air-dried, and dissolved in MQ. To the solution, 50 mM aqueous ammonium bicarbonate (NH_4HCO_3 aq.) and 25% propionic anhydride solution (methanol/propionic anhydride, 3:1 (vol/vol)) were added, and pH was adjusted to 8 by adding ammonia solution. After 1 h incubation at r.t., the solvents were removed by Speed-Vac evaporator. The propionylated proteins were treated as described in “General method for protein digestion for LC–MS/MS analysis”.

Supplementary Tables

Supplementary Table 1. LC–MS/MS conditions for the histone-derived peptides

From recombinant nucleosome (H2B)

**Peptide**	**Sequence**	**Digestion**	**Target precursor ions (*m/z*)[a]**	**Fragment ions**	**Collision energy**
H2B G114-K125	GTKAVTKYTSAK	Trypsin Glu-C	711.90 (3Pr, 2H$^+$)<br>704.89 (1Ac, 2H$^+$)<br>697.88 (2Ac, 2H$^+$)<br>690.87 (3Ac, 2H$^+$)<br>723.90 (1Acyl[b], 2H$^+$)<br>735.90 (2Acyl[b], 2H$^+$)<br>747.90 (3Acyl[b], 2H$^+$)	b_3(H$^+$), b_4(H$^+$), b_5(H$^+$), b_6(H$^+$), y_2(H$^+$), y_3(H$^+$), y_4(H$^+$), y_5(H$^+$)	40 V

From endogenous nucleosome (H2A)

**Peptide**	**Sequence**	**Digestion**	**Target precursor ions (*m/z*)[a]**	**Fragment ions**	**Collision energy**
H2A G4-R11	GKQGGKAR	Trypsin	457.26 (2Pr, 2H$^+$)<br>450.26 (1Ac, 2H$^+$)<br>443.25 (2Ac, 2H$^+$)	y_4(H$^+$), y_5(H$^+$), y_6(H$^+$)	35 V
H2A A12-R17	AKAKTR	Trypsin	393.75 (2Pr, 2H$^+$)<br>386.74 (1Ac, 2H$^+$)<br>379.73 (2Ac, 2H$^+$)	b_2(H$^+$), b_3(H$^+$), y_3(H$^+$), y_4(H$^+$)	35 V
H2A K36-R42	KGNYSER	Trypsin	455.22 (Pr, 2H$^+$)<br>448.22 (Ac, 2H$^+$)	y_5(H$^+$), y_6(H$^+$), y_7(H$^+$), y_8(H$^+$)	35 V
H2A D72-R77	DNKKTR	Trypsin	437.24 (2Pr, 2H$^+$)<br>430.24 (1Ac, 2H$^+$)<br>423.23 (2Ac, 2H$^+$)	b_3(H$^+$), y_3(H$^+$)	35 V
H2A L93-R99	LNKLLGR	Trypsin Glu-C	435.28 (Pr, 2H$^+$)<br>428.27 (Ac, 2H$^+$)	y_3(H$^+$), y_4(H$^+$), y_5(H$^+$), y_6(H$^+$)	35 V
H2A V100-E121	VTIAQGGVLPNIQ AVLLPKKTE	Trypsin Glu-C	801.14 (2Pr, 3H$^+$)<br>796.47 (1Ac, 3H$^+$)<br>791.80 (2Ac, 3H$^+$)	y_5(H$^+$), y_6(H$^+$), y_7(H$^+$), y_9(H$^+$)	35 V
H2A S122-K129	SHHKAKGK	Trypsin Glu-C	530.80 (3Pr, 2H$^+$)<br>523.79 (1Ac, 2H$^+$)<br>516.78 (2Ac, 2H$^+$)<br>509.78 (3Ac, 2H$^+$)	b_4(H$^+$), b_5(H$^+$), b_6(H$^+$), b_7(H$^+$), y_2(H$^+$), y_3(H$^+$), y_4(H$^+$)	35 V

From endogenous nucleosome (H2B)

Peptide	Sequence	Digestion	Target precursor ions (*m/z*)[a]	Fragment ions	Collision energy
H2B D25-R29	DGKKR	Trypsin Asp-N	358.21 (2Pr, $2H^+$) 351.20 (1Ac, $2H^+$) 344.19 (2Ac, $2H^+$)	$b_3(H^+)$, $y_2(H^+)$	35 V
H2B K34-P50	KESYSIYVYKVLK QVHP	Trypsin Asp-N	750.41 (3Pr, $3H^+$) 745.74 (1Ac, $3H^+$) 741.07 (2Ac, $3H^+$) 736.40 (3Ac, $3H^+$)	$b_8(H^+)$, $y_5(H^+)$, $y_6(H^+)$, $y_7(H^+)$, $y_8(H^+)$, $y_9(H^+)$	35 V
H2B D51-N67	DTGISSKAMGIM NSFVN	Trypsin Asp-N	914.43 (Pr, $2H^+$) 907.43 (Ac, $2H^+$)	$b_8(H^+)$, $b_9(H^+)$, $b_{11}(H^+)$, $y_6(H^+)$, $y_8(H^+)$	35 V
H2B L80-R86	LAHYNKR	Trypsin	479.27 (Pr, $2H^+$) 472.26 (Ac, $2H^+$)	$y_3(H^+)$, $y_4(H^+)$, $y_5(H^+)$, $y_6(H^+)$	35 V
H2B L106-E113	LAKHAVSE	Trypsin Glu-C	455.75 (Pr, $2H^+$) 448.75 (Ac, $2H^+$)	$b_3(H^+)$, $b_4(H^+)$, $b_5(H^+)$, $b_6(H^+)$	35 V
H2B G114-K125	GTKAVTKYTSSK	Trypsin Glu-C	719.89 (3Pr, $2H^+$) 712.89 (1Ac, $2H^+$) 705.88 (2Ac, $2H^+$) 698.87 (3Ac, $2H^+$)	$b_3(H^+)$, $b_4(H^+)$, $b_5(H^+)$, $y_2(H^+)$, $y_3(H^+)$, $y_4(H^+)$, $y_5(H^+)$, $y_7(H^+)$	40 V
H2B G114-K125 (Treated [U-^{13}C]-glucose)	GTKAVTKYTSSK	Trypsin Glu-C	713.89 (1Ac*, $2H^+$)	Shown in MS/MS charts in **Fig. 4d**	40 V

From endogenous nucleosome (H3)

Peptide	Sequence	Digestion	Target precursor ions (*m/z*)[a]	Fragment ions	Collision energy
H3 T3-R8	TKQTAR	Trypsin	380.72 (Pr, $2H^+$)<br>373.71 (Ac, $2H^+$)	$y_2(H^+)$, $y_3(H^+)$, $y_4(H^+)$, $y_5(H^+)$	35 V
H3 K9-R17	KSTGGKAPR	Trypsin	507.29 (2Pr, $2H^+$)<br>500.28 (1Ac, $2H^+$)<br>493.27 (2Ac, $2H^+$)	$y_5(H^+)$, $y_6(H^+)$, $y_7(H^+)$, $y_8(H^+)$	35 V
H3 K18-R26	KQLATKAAR	Trypsin	549.84 (2Pr, $2H^+$)<br>542.83 (1Ac, $2H^+$)<br>535.82 (2Ac, $2H^+$)	$y_5(H^+)$, $y_6(H^+)$, $y_7(H^+)$, $y_8(H^+)$	35 V
H3 K18-R26 (Treated [U-^{13}C]-glucose)	KQLATKAAR	Trypsin	543.83 (1Ac*, $2H^+$)	Shown in MS/MS charts in **Supplementary Fig. 14**	35 V
H3 K27-R40	KSAPATGGVKKPHR	Trypsin	534.64 (3Pr, $3H^+$)<br>529.97 (1Ac, $3H^+$)<br>525.30 (2Ac, $3H^+$)<br>520.63 (3Ac, $3H^+$)	$y_4(H^+)$, $y_5(H^+)$, $y_6(H^+)$, $y_7(H^+)$, $y_8(H^+)$	35 V
H3 Y54-R63	YQKSTELLIR	Trypsin	653.87 (Pr, $2H^+$)<br>646.86 (Ac, $2H^+$)	$b_3(H^+)$, $y_6(H^+)$, $y_7(H^+)$, $y_8(H^+)$	35 V
H3 K64-R69	KLPFQR	Trypsin	422.76 (Pr, $2H^+$)<br>415.75 (Ac, $2H^+$)	$y_2(H^+)$, $y_3(H^+)$, $y_4(H^+)$, $y_5(H^+)$	35 V
H3 E73-R83	EIAQDFKTDLR	Trypsin	696.36 (Pr, $2H^+$)<br>689.35 (Ac, $2H^+$)	$y_5(H^+)$, $y_6(H^+)$, $y_7(H^+)$, $y_8(H^+)$	35 V
H3 V117-R128	VTIMPKDIQLAR	Trypsin	720.92 (Pr, $2H^+$)<br>713.91 (Ac, $2H^+$)	$b_3(H^+)$, $y_8(H^+)$, $y_9(H^+)$, $y_{10}(H^+)$	35 V

From endogenous nucleosome (H4)

Peptide	Sequence	Digestion	Target precursor ions (*m/z*)[a]	Fragment ions	Collision energy
H4 G4-R17	GKGGKGLGKGG AKR	Trypsin	747.94 (4Pr, $2H^+$)<br>740.93 (1Ac, $2H^+$)<br>733.93 (2Ac, $2H^+$)<br>726.92 (3Ac, $2H^+$)<br>719.91 (4Ac, $2H^+$)	$b_2(H^+)$, $b_3(H^+)$, $b_4(H^+)$, $y_3(H^+)$, $y_4(H^+)$, $y_5(H^+)$, $y_6(H^+)$, $y_7(H^+)$, $y_8(H^+)$, $y_9(H^+)$	45 V
H4 K20-R23	KVLR	Trypsin	286.20 (Pr, $2H^+$)<br>279.19 (Ac, $2H^+$)	$y_2(H^+)$, $y_3(H^+)$	35 V
H4 D24-R35	DNIQGITKPAIR	Trypsin	691.39 (Pr, $2H^+$)<br>684.39 (Ac, $2H^+$)	$y_6(H^+)$, $y_7(H^+)$, $y_8(H^+)$, $y_9(H^+)$	40 V
H4 G41-R45	GGVKR	Trypsin	286.68 (Pr, $2H^+$)<br>279.67 (Ac, $2H^+$)	$y_2(H^+)$, $y_3(H^+)$	35 V
H4 G56-R67	GVLKVFLENVIR	Trypsin	721.94 (Pr, $2H^+$)<br>714.93 (Ac, $2H^+$)	$y_6(H^+)$, $y_7(H^+)$, $y_8(H^+)$, $y_9(H^+)$	40 V
H4 D68-R78	DAVTYTEHAKR	Trypsin	673.84 (Pr, $2H^+$)<br>666.83 (Ac, $2H^+$)	$y_5(H^+)$, $y_6(H^+)$, $y_7(H^+)$, $y_8(H^+)$	45 V
H4 K79-R92	KTVTAMDVVYAL KR	Trypsin	853.98 (2Pr, $2H^+$)<br>846.97 (1Ac, $2H^+$)<br>839.96 (2Ac, $2H^+$)	$y_8(H^+)$, $y_9(H^+)$, $y_{10}(H^+)$, $y_{11}(H^+)$	40 V

[a] *n*Acyl and *n*Ac in parenthesis indicates *n* lysines acylated and the other lysines propionylated on the corresponding peptide.

[b] Acyl group = Alkyne-modified acyl group (Pentynoyl group)

*Acetyl group containing two isotopic labeled carbons.

Supplementary Table 2. LC–MS/MS conditions for the androgen receptor-derived peptide

Peptide	Sequence	Digestion	Target precursor ions (*m/z*)[a]	Fragment ions	Collision energy
AR Q714-R729	QLVHVVKWAKAL PGFR	Trypsin Glu-C	654.39 (2Pr, $3H^+$)<br>662.39 (1Acyl[b], $3H^+$)<br>670.39 (2Acyl[b], $3H^+$)	$b_9(H^+)$, $y_8(H^+)$, $y_9(H^+)$	40 V

[a] *n*Acyl in parenthesis indicates *n* lysines acylated and the other lysines propionylated on the corresponding peptide.

[b] Acyl group = Alkyne-modified acyl group (Pentynoyl group)

Supplementary Table 3. Primers for ChIP assay[10]

Primers	Sequence (5'–3')	Chromatin structure
Rhob-fw	CCTGGTGGCCAACAAAAAAG	Euchromatin
Rhob-rv	TCTGTGCGGACATGCTCGT	
ADH5-fw	GCATAATTGAGCCTACGCC	Euchromatin
ADH5-rv	GCAGAGGTGTTTGTTACGTG	
GAPDH-fw	CCGGGAGAAGCTGAGTCATG	Euchromatin
GAPDH-rv	TTTGCGGTGGAAATGTCCTT	
Chr1-fw	CATCGATGGAAATGAAAGGAGTC	Centromeric, constitutive heterochromatin
Chr1-rv	ACCATTGGATGATTGCAGTCAA	
Chr4-fw	CTGCACTACCTGAAGAGGAC	Centromeric, constitutive heterochromatin
Chr4-rv	GATGGTTCAACACTCTTACA	
Oct4-fw	GTGGAGGAAGCTGACAACAA	Facultative heterochromatin
Oct4-rv	ATTCTCCAGGTTGCCTCTCA	
Nanog-fw	CAAAGGCAAACAACCCACTT	Facultative heterochromatin
Nanog-rv	TCTGCTGGAGGCTGAGGTAT	

<u>**Supplementary References**</u>

1. Mizumoto, S. *et al.* Hydroxamic Acid‐Piperidine Conjugate is an Activated Catalyst for Lysine Acetylation under Physiological Conditions. *Chem. – An Asian J.* **15**, 833–839 (2020).
2. Fujimura, A. *et al.* Chemical catalyst/protein hybrid as artificial histone-modifying enzyme for epigenome manipulation. *ChemRxiv* 1–15 (2022) doi:10.26434/chemrxiv-2022-xt610.
3. Amamoto, Y. *et al.* Synthetic Posttranslational Modifications: Chemical Catalyst-Driven Regioselective Histone Acylation of Native Chromatin. *J. Am. Chem. Soc.* **139**, 7568–7576 (2017).
4. Zhang, Y., Mantravadi, P. K., Jobbagy, S., Bao, W. & Koh, J. T. Antagonizing the Androgen Receptor with a Biomimetic Acyltransferase. *ACS Chem. Biol.* **11**, 2797–2802 (2016).
5. Jin, Q. *et al.* Distinct roles of GCN5/PCAF-mediated H3K9ac and CBP/p300-mediated H3K18/27ac in nuclear receptor transactivation. *EMBO J.* **30**, 249–262 (2011).
6. Sabari, B. R. *et al.* Intracellular Crotonyl-CoA Stimulates Transcription through p300-Catalyzed Histone Crotonylation. *Mol. Cell* **58**, 203–215 (2015).
7. Hacker, S. M. *et al.* Global profiling of lysine reactivity and ligandability in the human proteome. *Nat. Chem.* **9**, 1181–1190 (2017).
8. Hamajima, W. *et al.* Site-Selective Synthetic Acylation of a Target Protein in Living Cells Promoted by a Chemical Catalyst/Donor System. *ACS Chem. Biol.* **14**, 1102–1109 (2019).
9. Molla, M. R., Prasad, P. & Thayumanavan, S. Protein-Induced Supramolecular Disassembly of Amphiphilic Polypeptide Nanoassemblies. *J. Am. Chem. Soc.* **137**, 7286–7289 (2015).
10. David, Y., Vila-Perelló, M., Verma, S. & Muir, T. W. Chemical tagging and customizing of cellular chromatin states using ultrafast trans-splicing inteins. *Nat. Chem.* **7**, 394–402 (2015).

Supplementary Spectral Data

Supplementary Spectral Data 1. ^{1}H and ^{13}C NMR spectra of compound S7.

Supplementary Spectral Data

Supplementary Spectral Data 1. ^{1}H and ^{13}C NMR spectra of compound S7

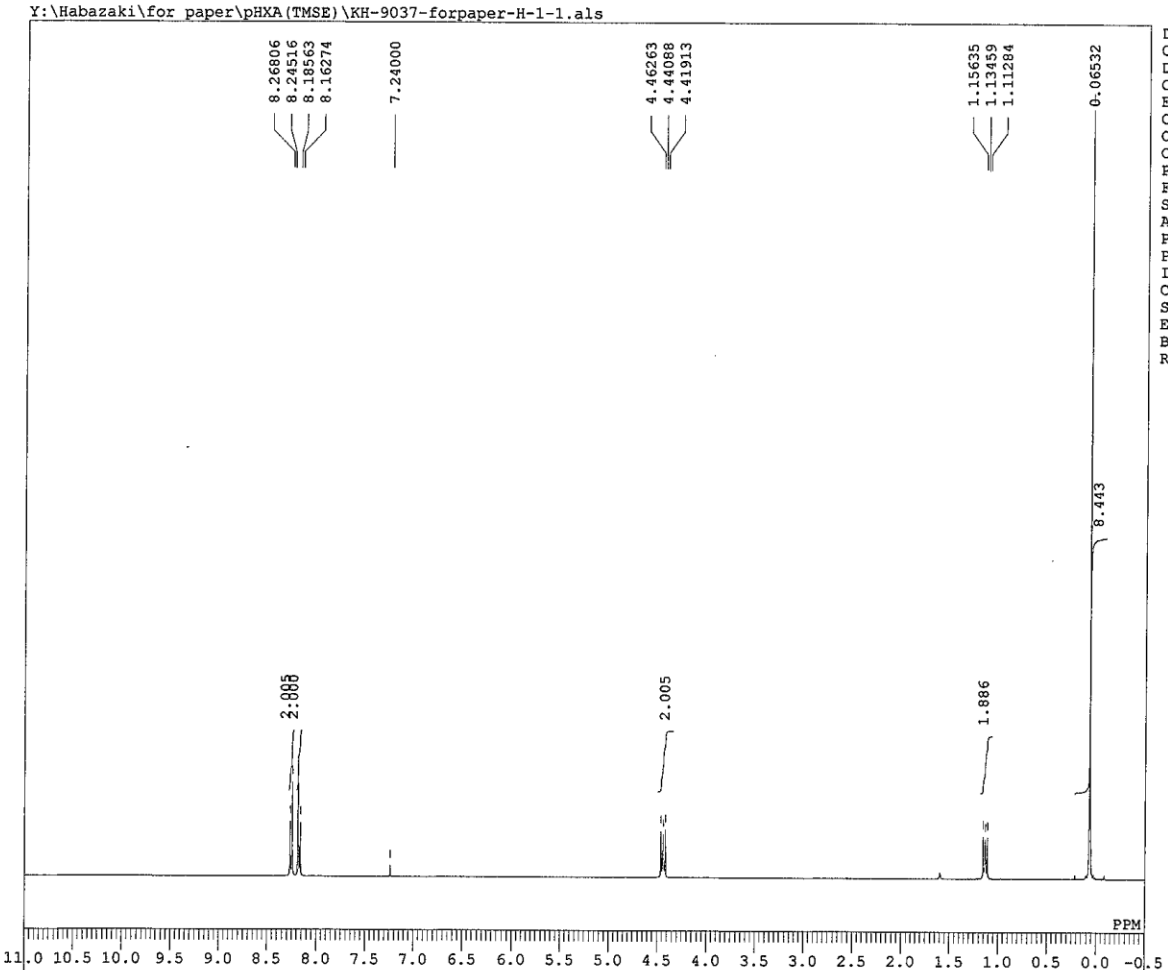

DFILE KH-9037-forpaper-H-1-1.als
COMNT
DATIM 11-07-2020 12:17:58
OBNUC 1H
EXMOD proton.jxp
OBFRQ 391.78 MHz
OBSET 8.51 KHz
OBFIN 3.34 Hz
POINT 13107
FREQU 5878.90 Hz
SCANS 8
ACQTM 2.2295 sec
PD 6.0000 sec
PW1 5.17 usec
IRNUC 1H
CTEMP 20.9 c
SLVNT CDCL3
EXREF 7.24 ppm
BF 0.62 Hz
RGAIN 30

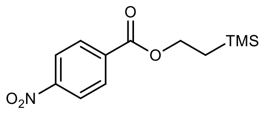

S7

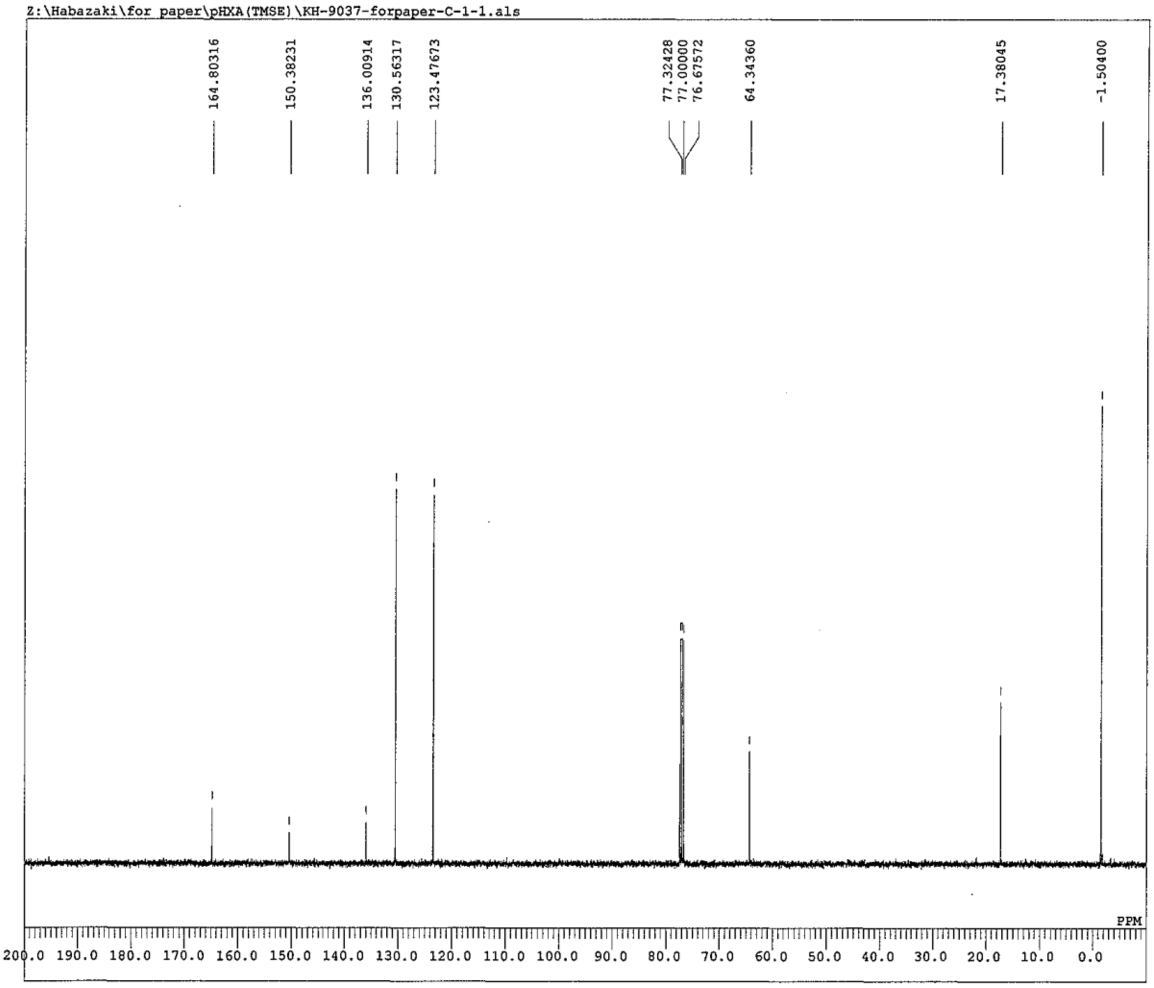

DFILE KH-9037-forpaper-C-1-1.als
COMNT
DATIM 11-07-2020 12:20:45
OBNUC 13C
EXMOD carbon.jxp
OBFRQ 98.52 MHz
OBSET 4.64 KHz
OBFIN 8.74 Hz
POINT 26214
FREQU 24630.54 Hz
SCANS 102
ACQTM 1.0643 sec
PD 2.0000 sec
PW1 3.12 usec
IRNUC 1H
CTEMP 20.7 c
SLVNT CDCL3
EXREF 77.00 ppm
BF 0.62 Hz
RGAIN 60

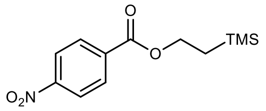

S7

Supplementary Spectral Data 2. ^{1}H and ^{13}C NMR spectra of compound S9

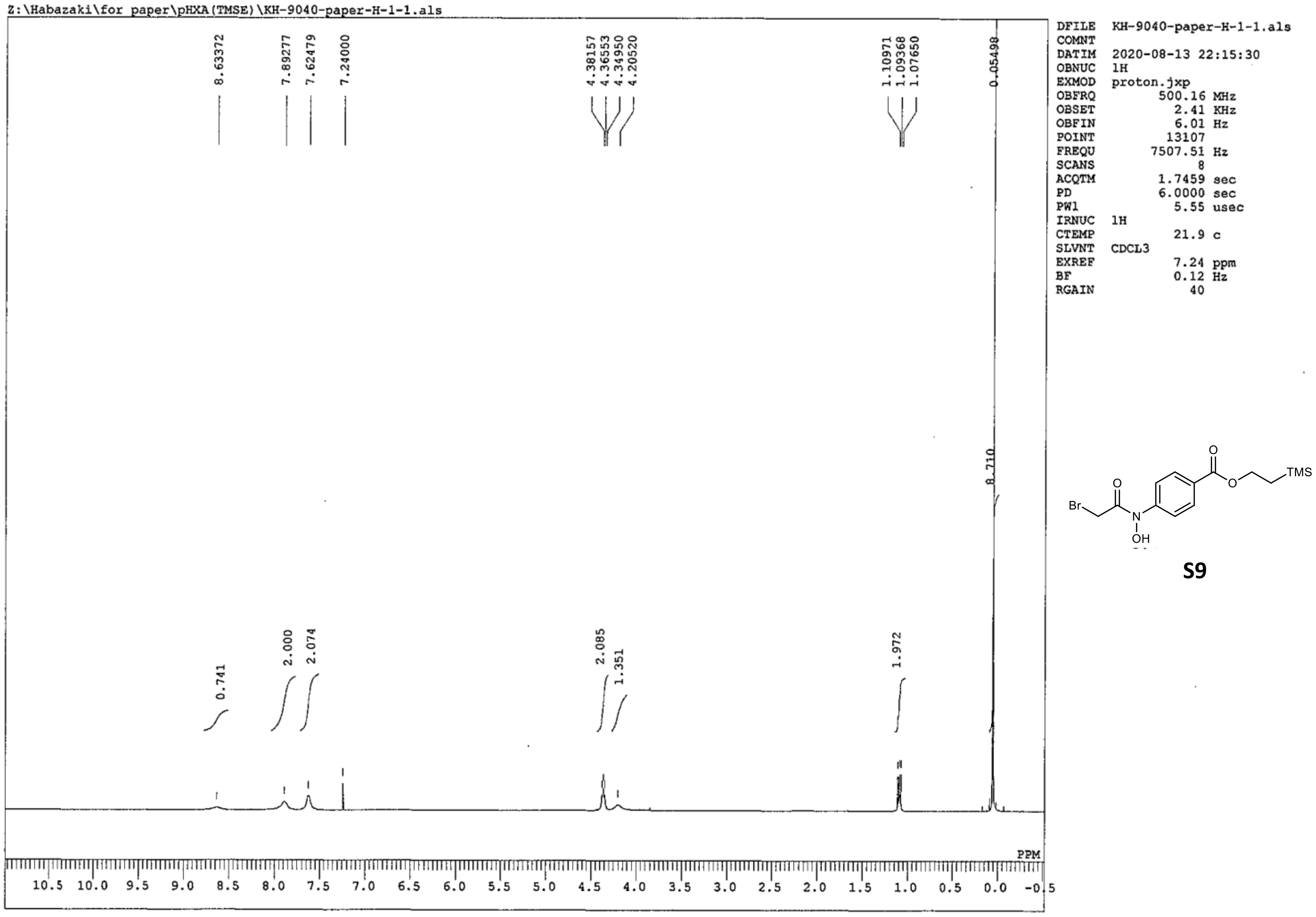

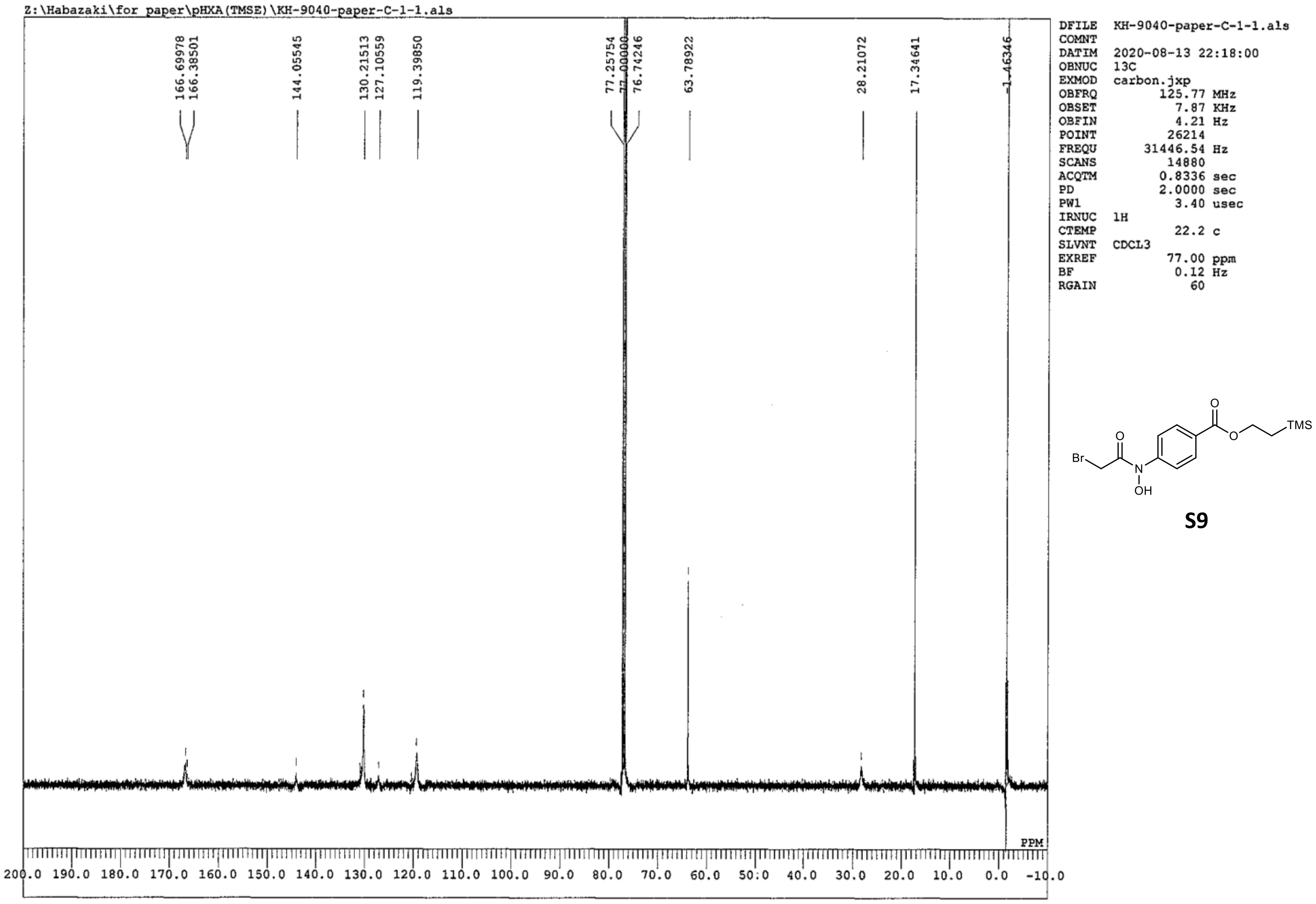

Supplementary Spectral Data 3. ^{1}H and ^{13}C NMR spectra of compound S10

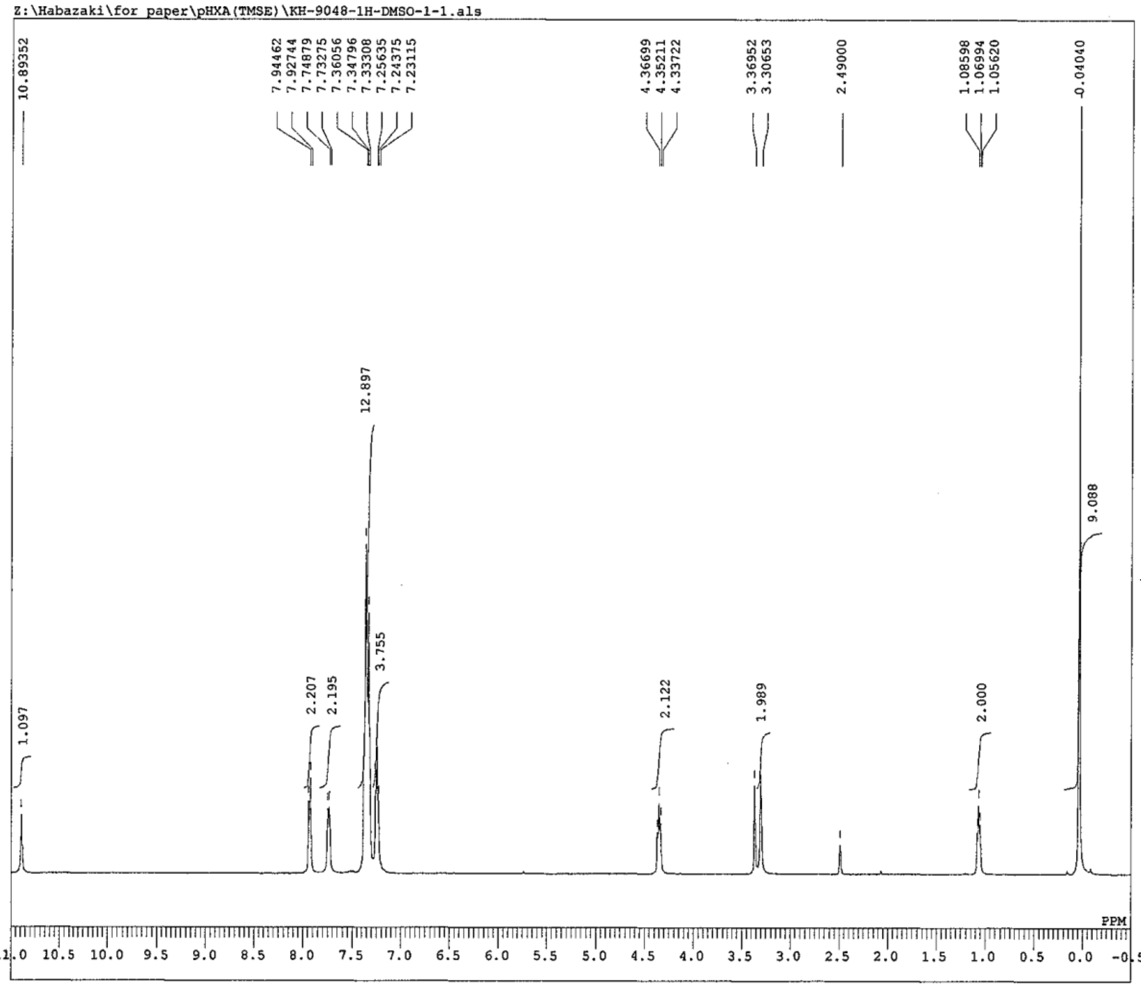

DFILE KH-9048-1H-DMSO-1-1.als
COMNT
DATIM 2022-03-06 15:12:32
OBNUC 1H
EXMOD proton.jxp
OBFRQ 500.16 MHz
OBSET 2.41 KHz
OBFIN 6.01 Hz
POINT 13107
FREQU 7507.51 Hz
SCANS 8
ACQTM 1.7459 sec
PD 6.0000 sec
PW1 5.55 usec
IRNUC 1H
CTEMP 21.8 c
SLVNT DMSO
EXREF 2.49 ppm
BF 0.12 Hz
RGAIN 38

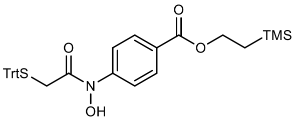

S10

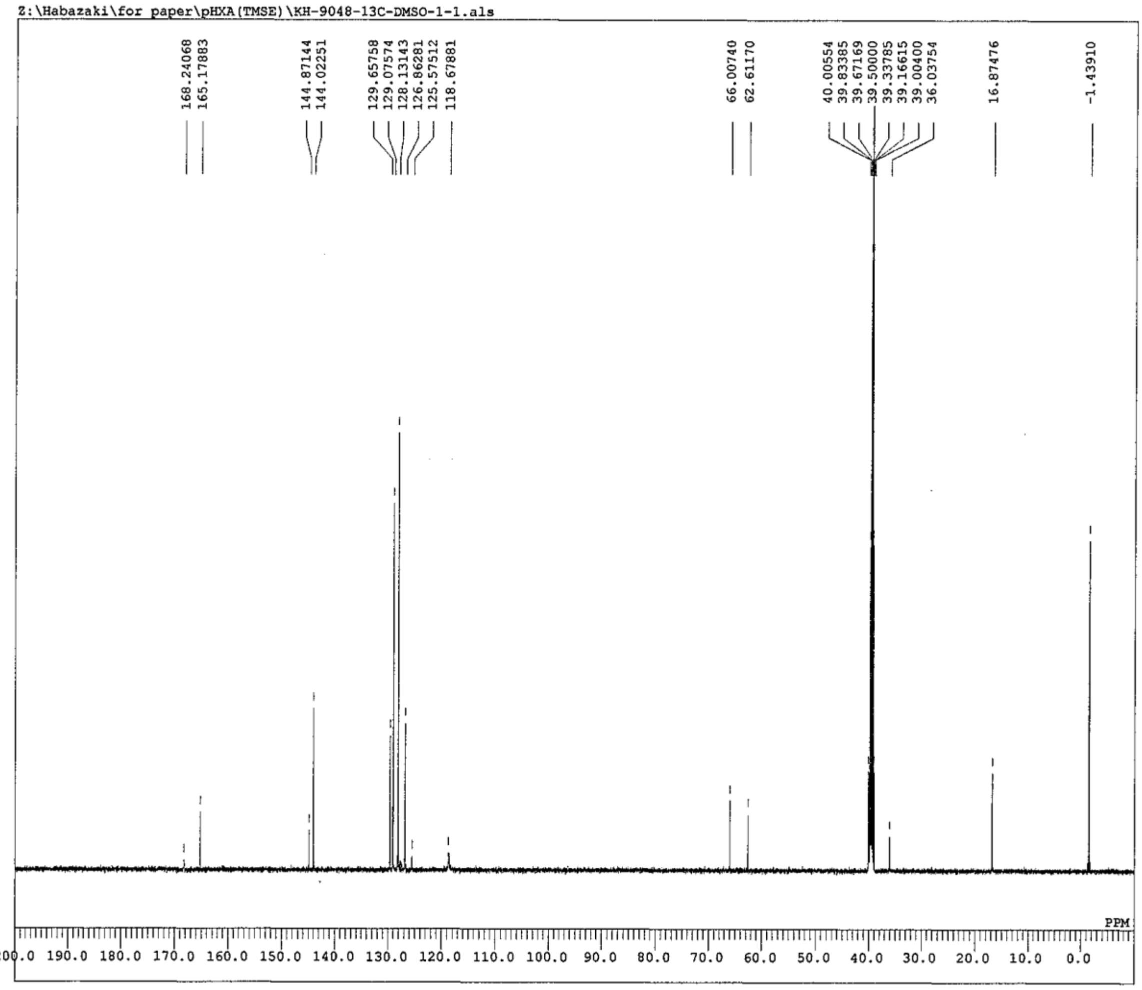

DFILE KH-9048-13C-DMSO-1-1.als
COMNT
DATIM 2022-03-06 15:14:40
OBNUC 13C
EXMOD carbon.jxp
OBFRQ 125.77 MHz
OBSET 7.87 KHz
OBFIN 4.21 Hz
POINT 26214
FREQU 31446.54 Hz
SCANS 22943
ACQTM 0.8336 sec
PD 2.0000 sec
PW1 3.40 usec
IRNUC 1H
CTEMP 22.0 c
SLVNT DMSO
EXREF 39.50 ppm
BF 0.12 Hz
RGAIN 60

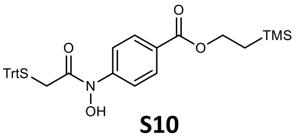

S10

Supplementary Spectral Data 4. ^{1}H and ^{13}C NMR spectra of compound S11

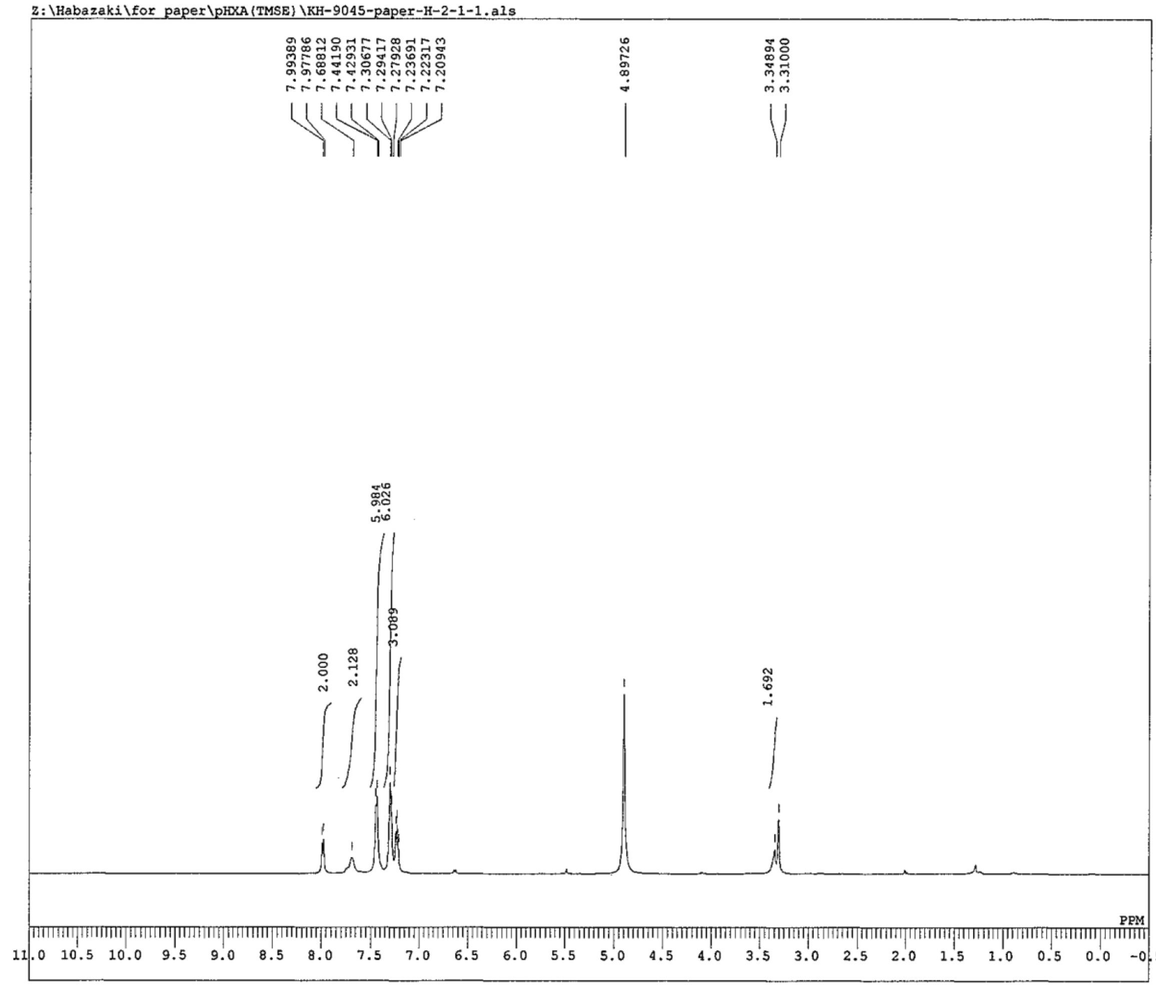


```
DFILE   KH-9045-paper-H-2-1-1.als
COMNT
DATIM   2020-08-05 23:06:27
OBNUC   1H
EXMOD   proton.jxp
OBFRQ        500.16 MHz
OBSET          2.41 KHz
OBFIN          6.01 Hz
POINT         13107
FREQU       7507.51 Hz
SCANS             8
ACQTM        1.7459 sec
PD           6.0000 sec
PW1            5.55 usec
IRNUC   1H
CTEMP          21.5 c
SLVNT   CD3OD
EXREF          3.31 ppm
BF             4.20 Hz
RGAIN            40
```

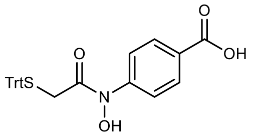

S11

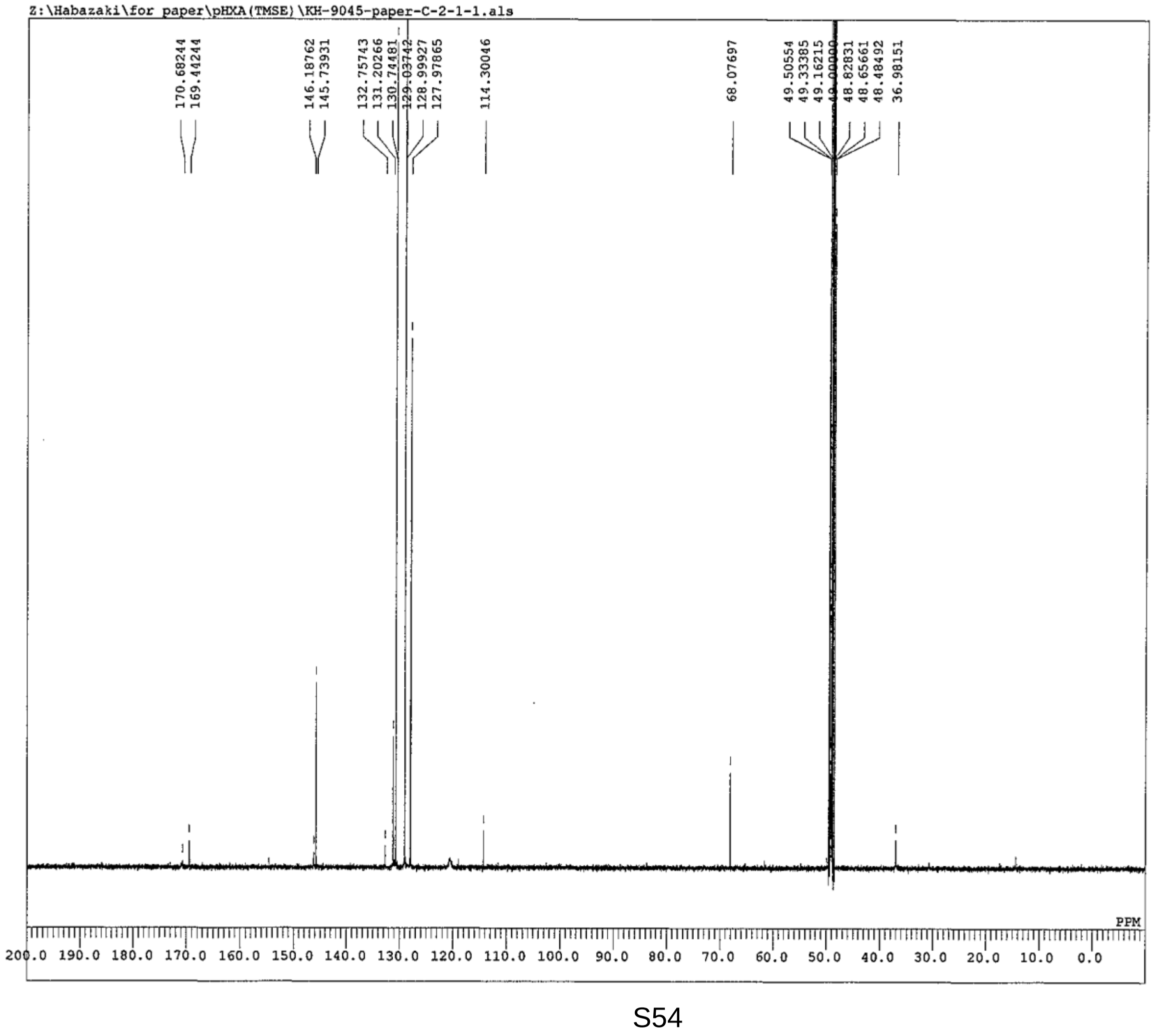


```
DFILE   KH-9045-paper-C-2-1-1.als
COMNT
DATIM   2020-08-05 23:12:34
OBNUC   13C
EXMOD   carbon.jxp
OBFRQ        125.77 MHz
OBSET          7.87 KHz
OBFIN          4.21 Hz
POINT         26214
FREQU      31446.54 Hz
SCANS         12883
ACQTM        0.8336 sec
PD           2.0000 sec
PW1            3.40 usec
IRNUC   1H
CTEMP          22.4 c
SLVNT   CD3OD
EXREF         49.00 ppm
BF             0.12 Hz
RGAIN            60
```

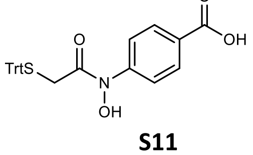

S11

Supplementary Spectral Data 5. ^{1}H and ^{13}C NMR spectra of compound 1

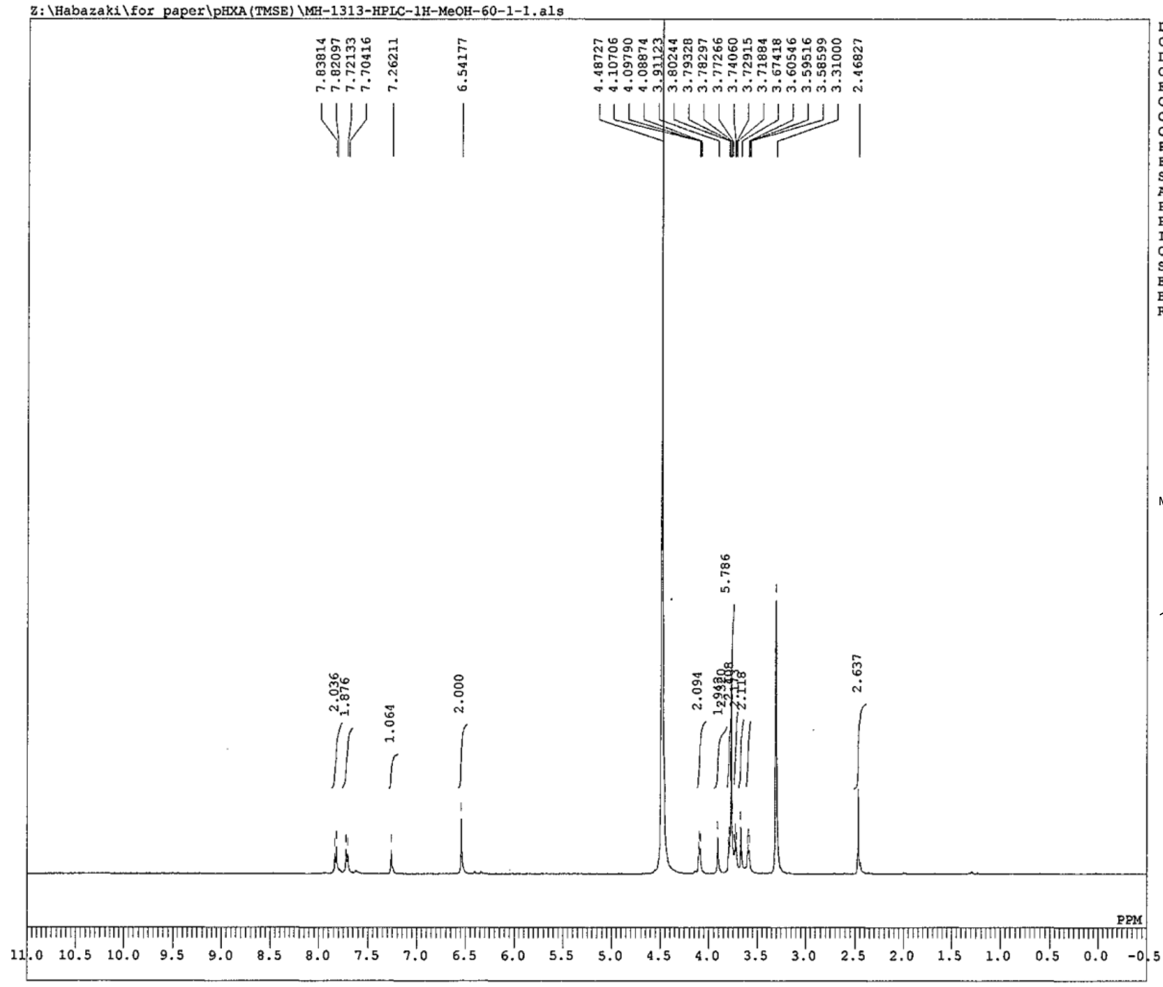

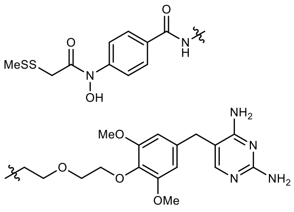

1

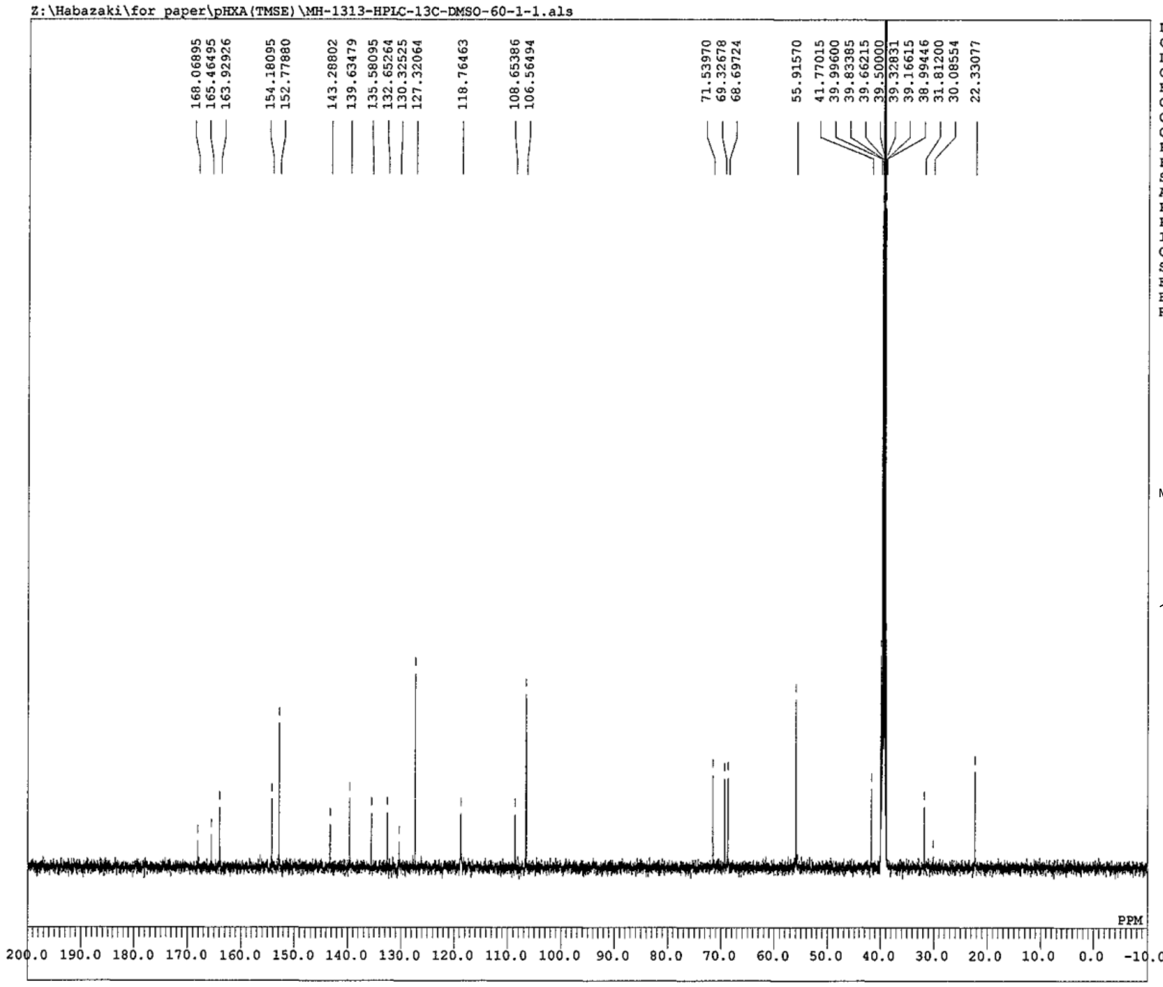

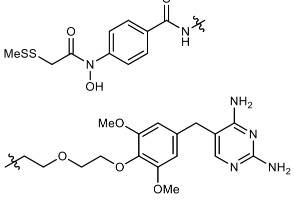

1

Supplementary Spectral Data 6. ^{1}H and ^{13}C NMR spectra of compound S16

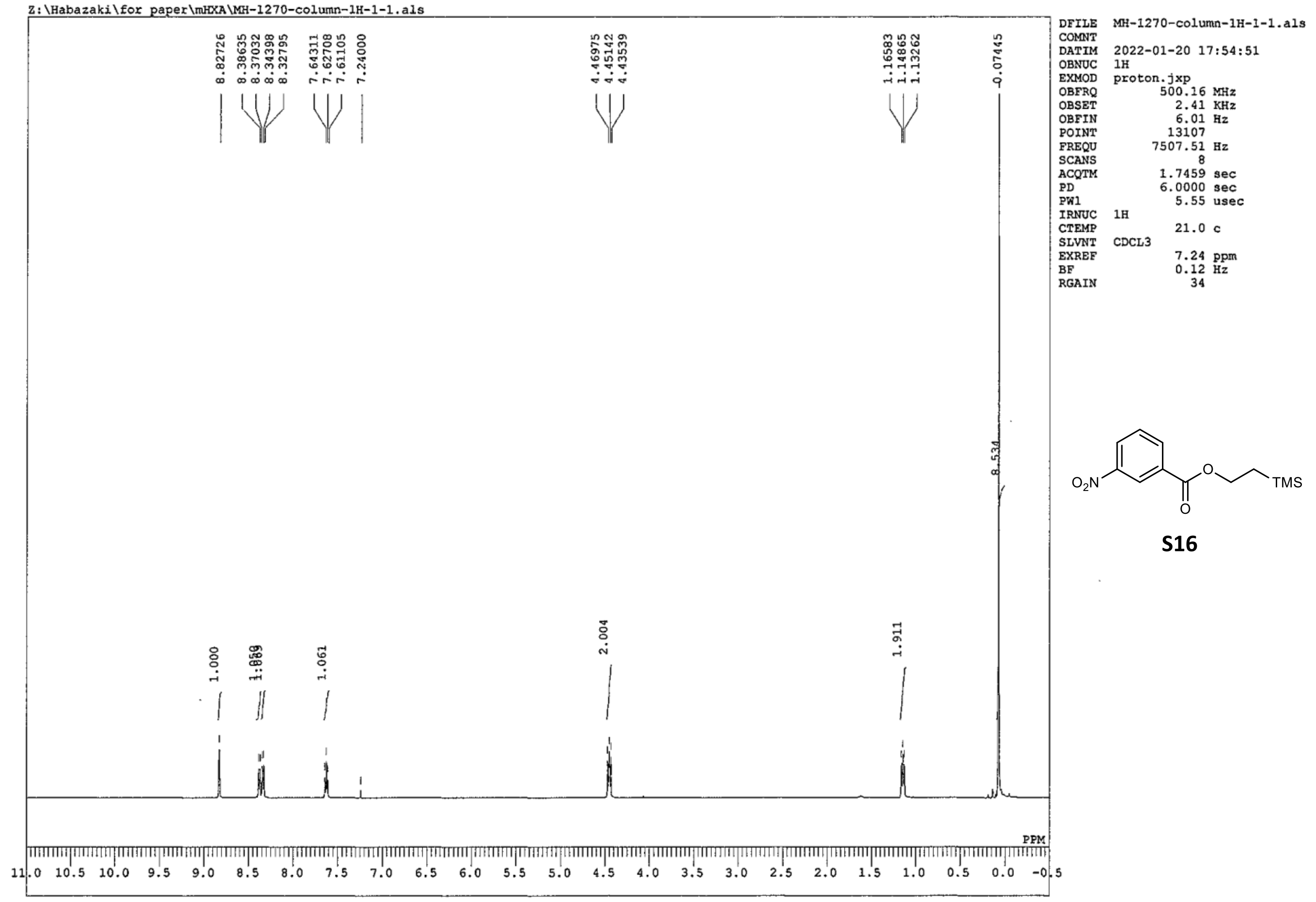

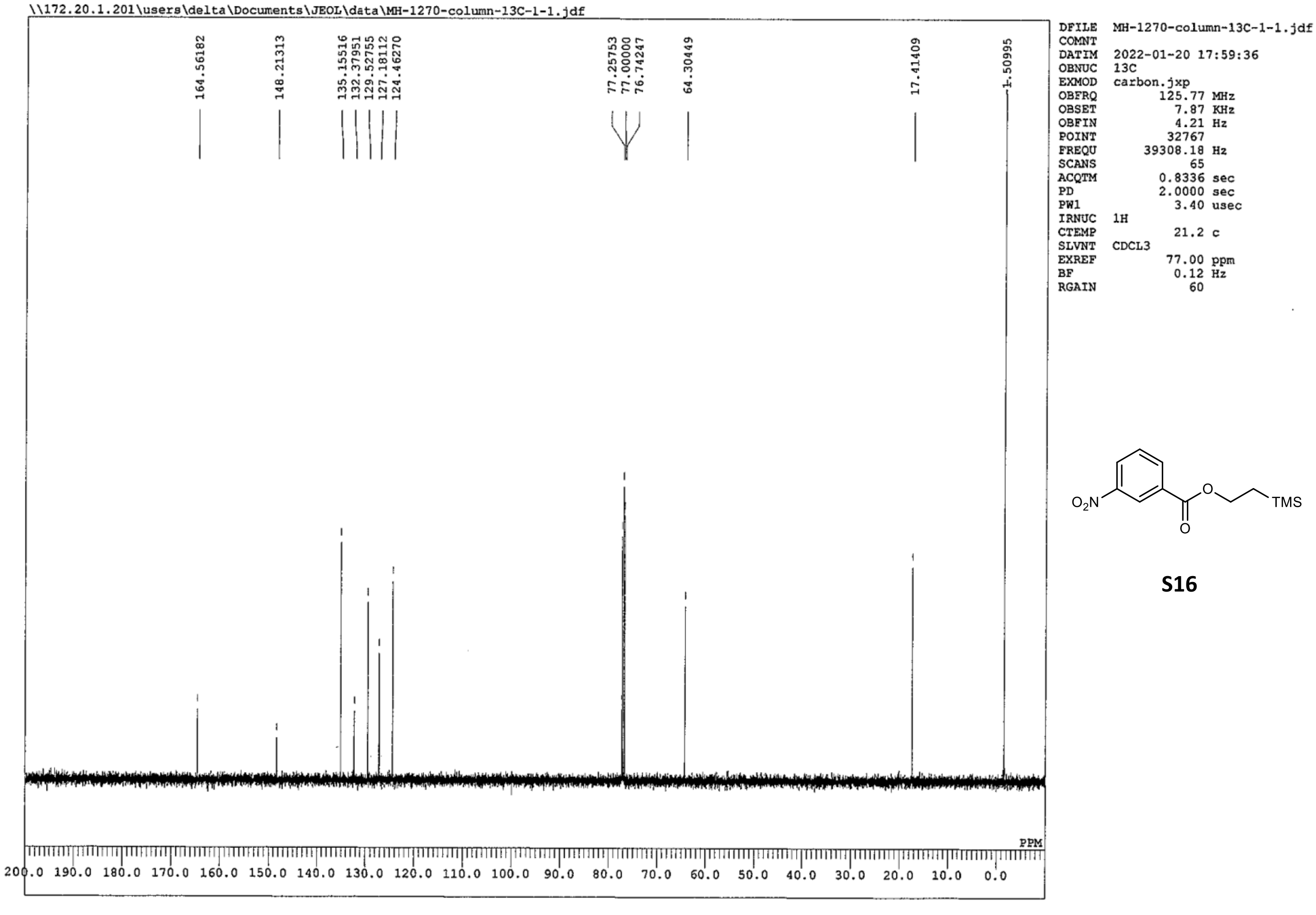

Supplementary Spectral Data 7. ^{1}H and ^{13}C NMR spectra of compound S18

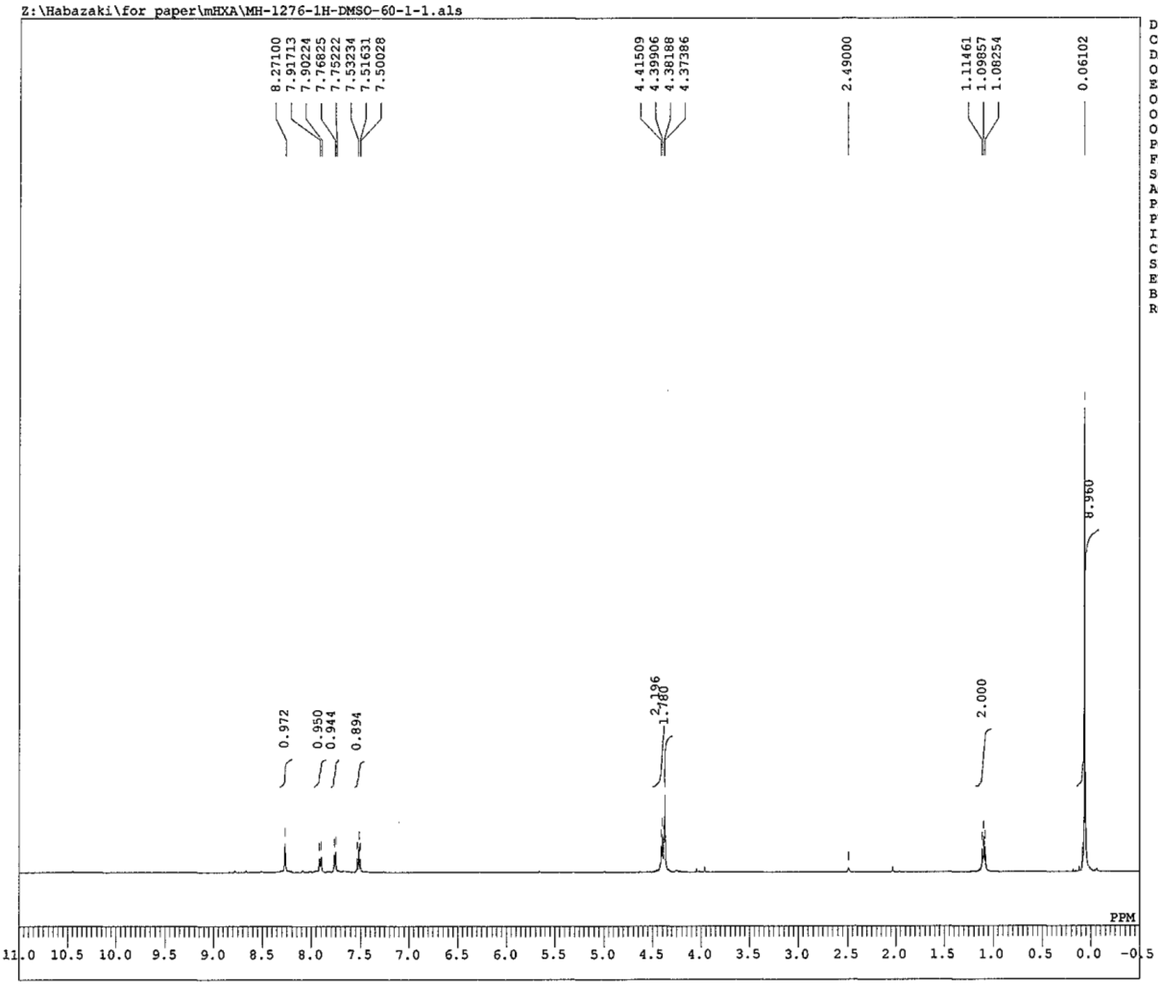

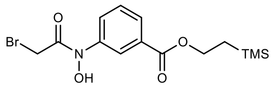

S18

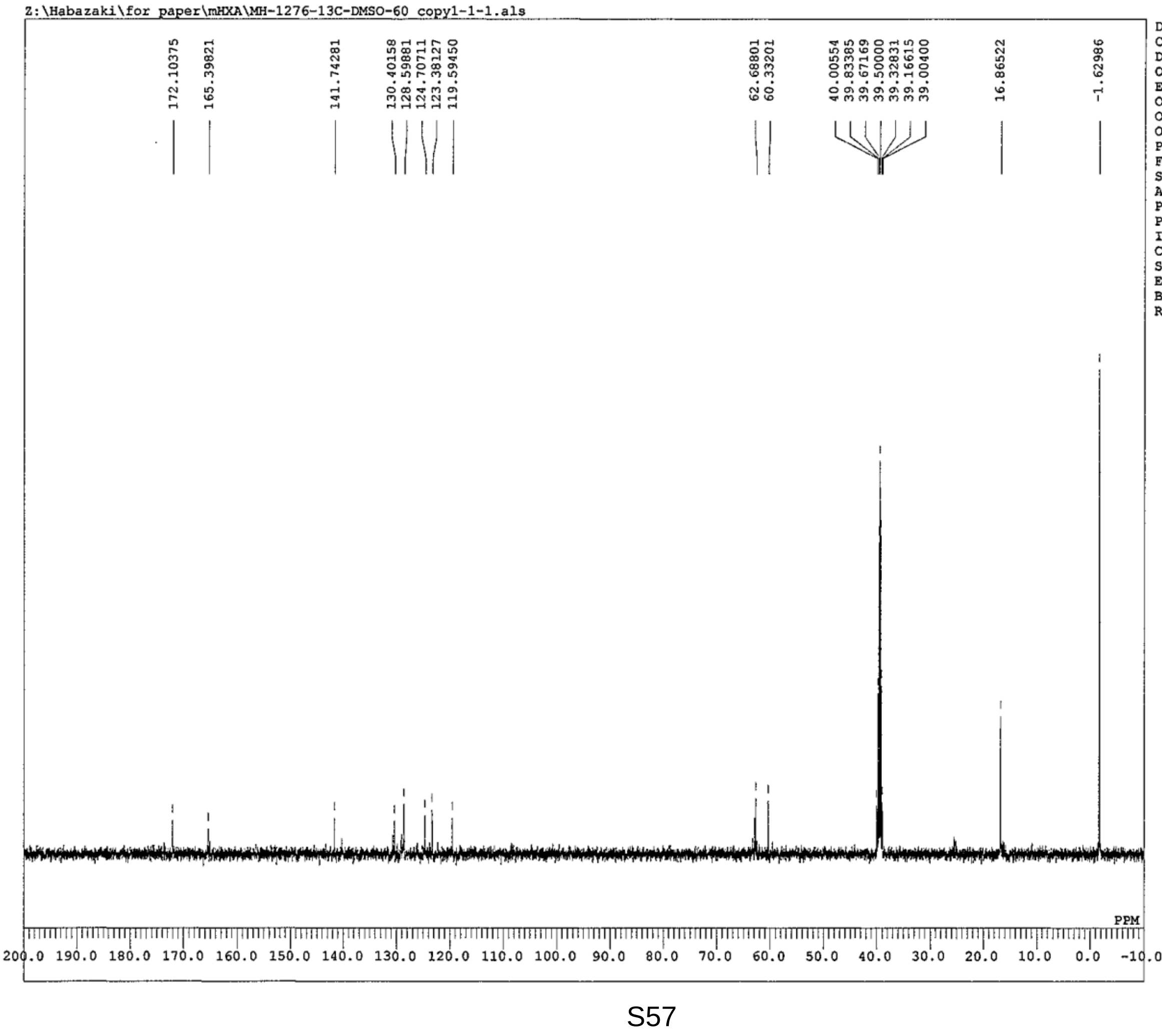

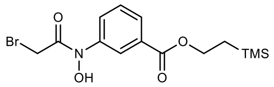

S18

Supplementary Spectral Data 8. ^{1}H and ^{13}C NMR spectra of compound S19

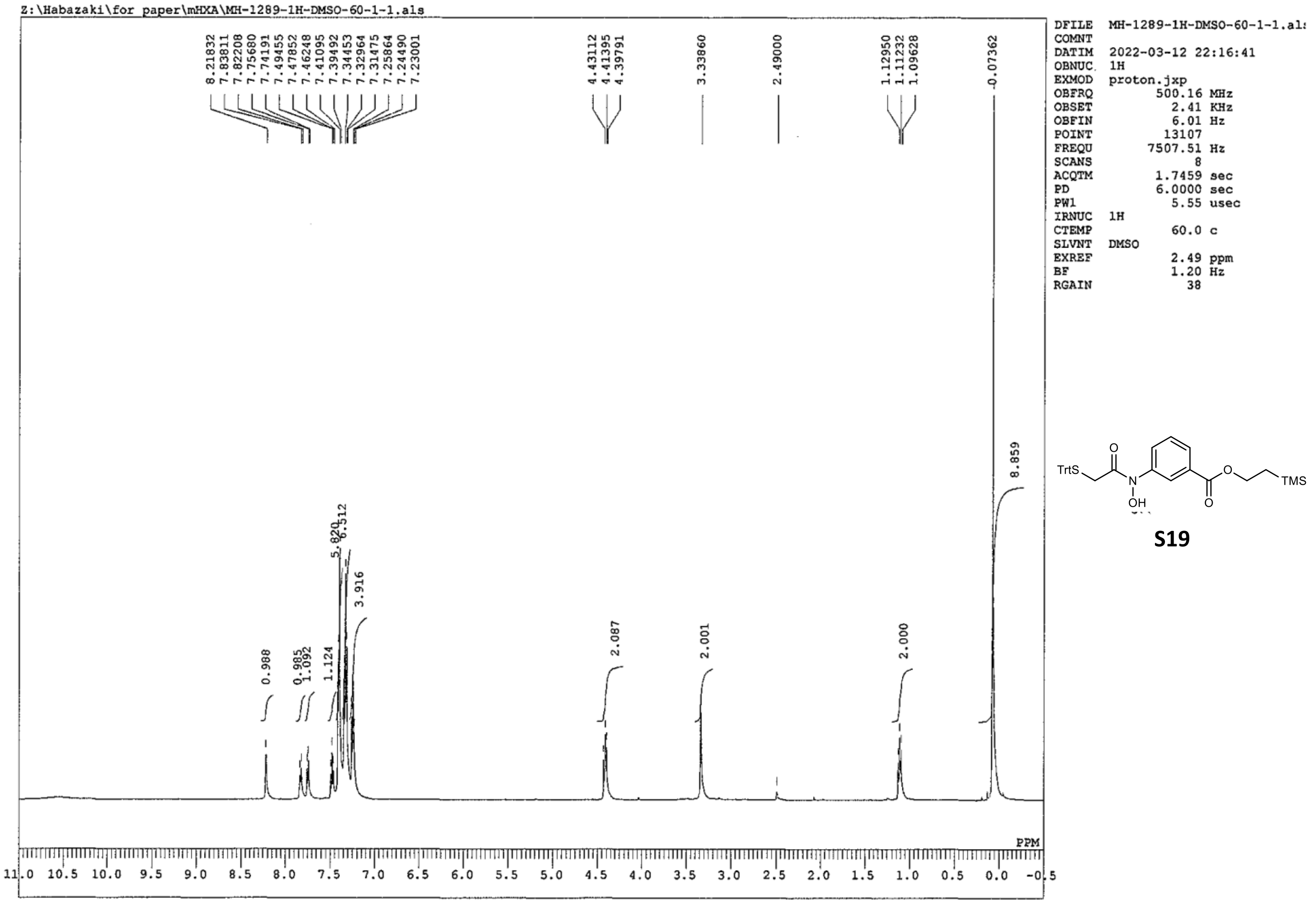

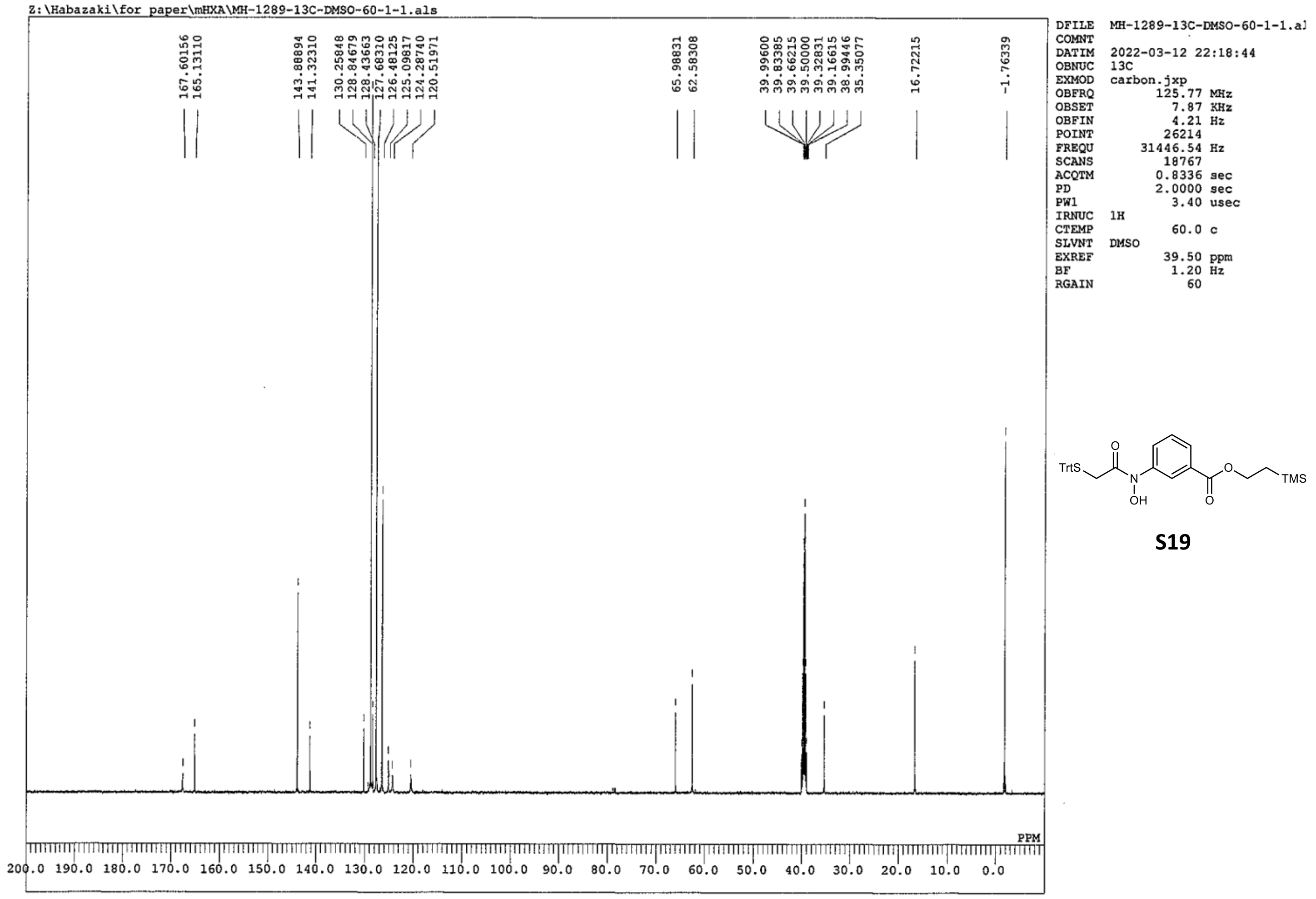

Supplementary Spectral Data 9. ^{1}H and ^{13}C NMR spectra of compound S21

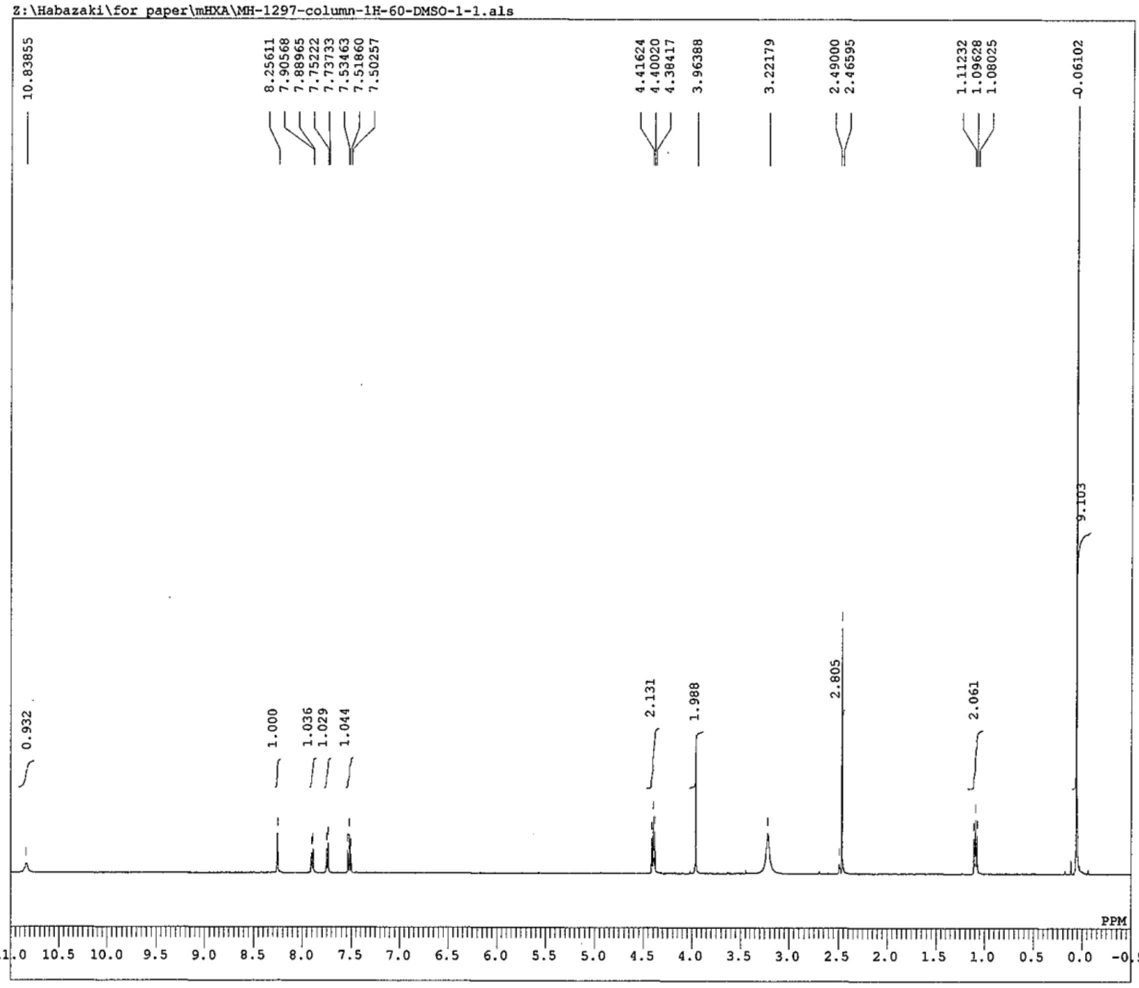

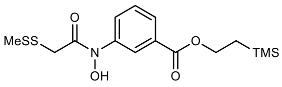

S21

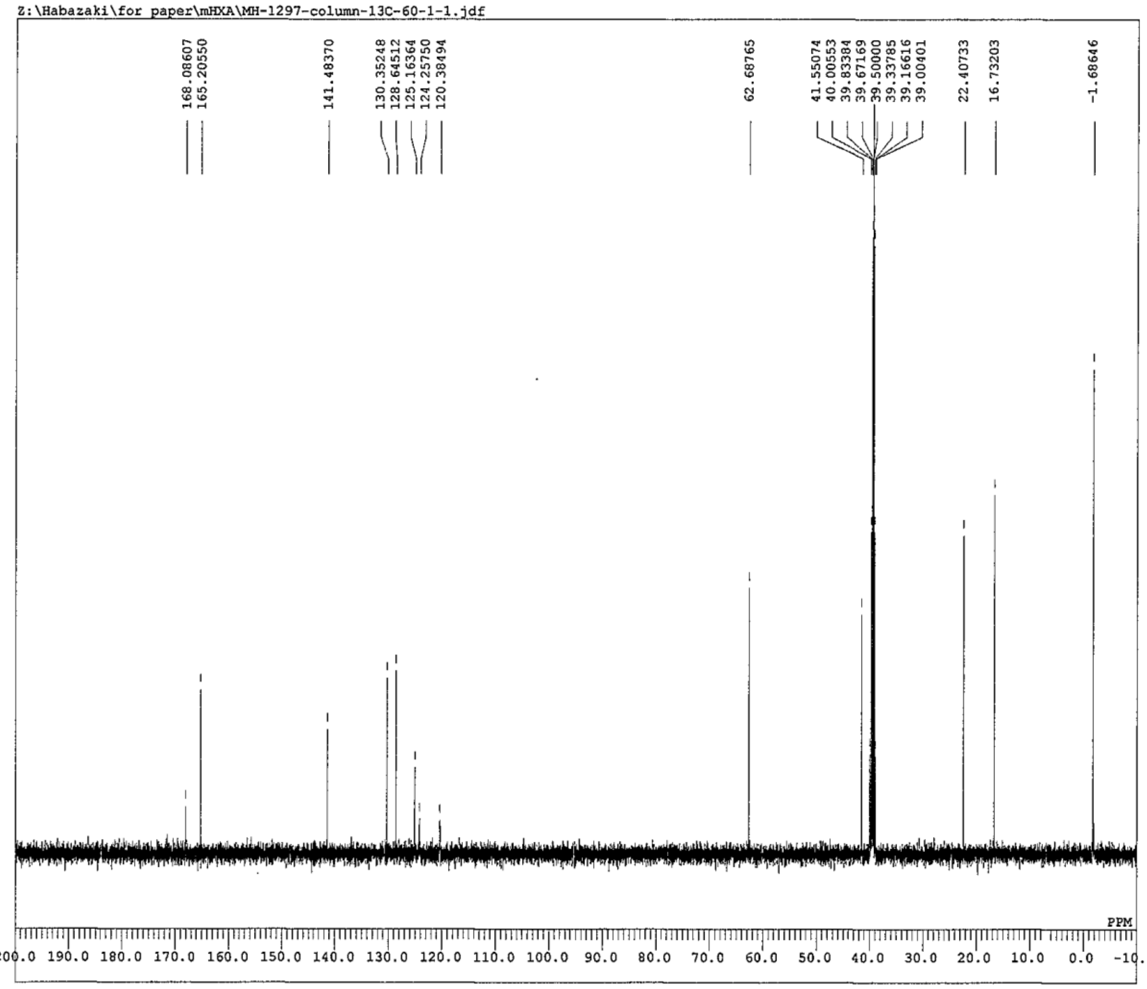

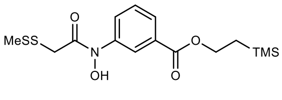

S21

Supplementary Spectral Data 10. ^{1}H and ^{13}C NMR spectra of compound S22

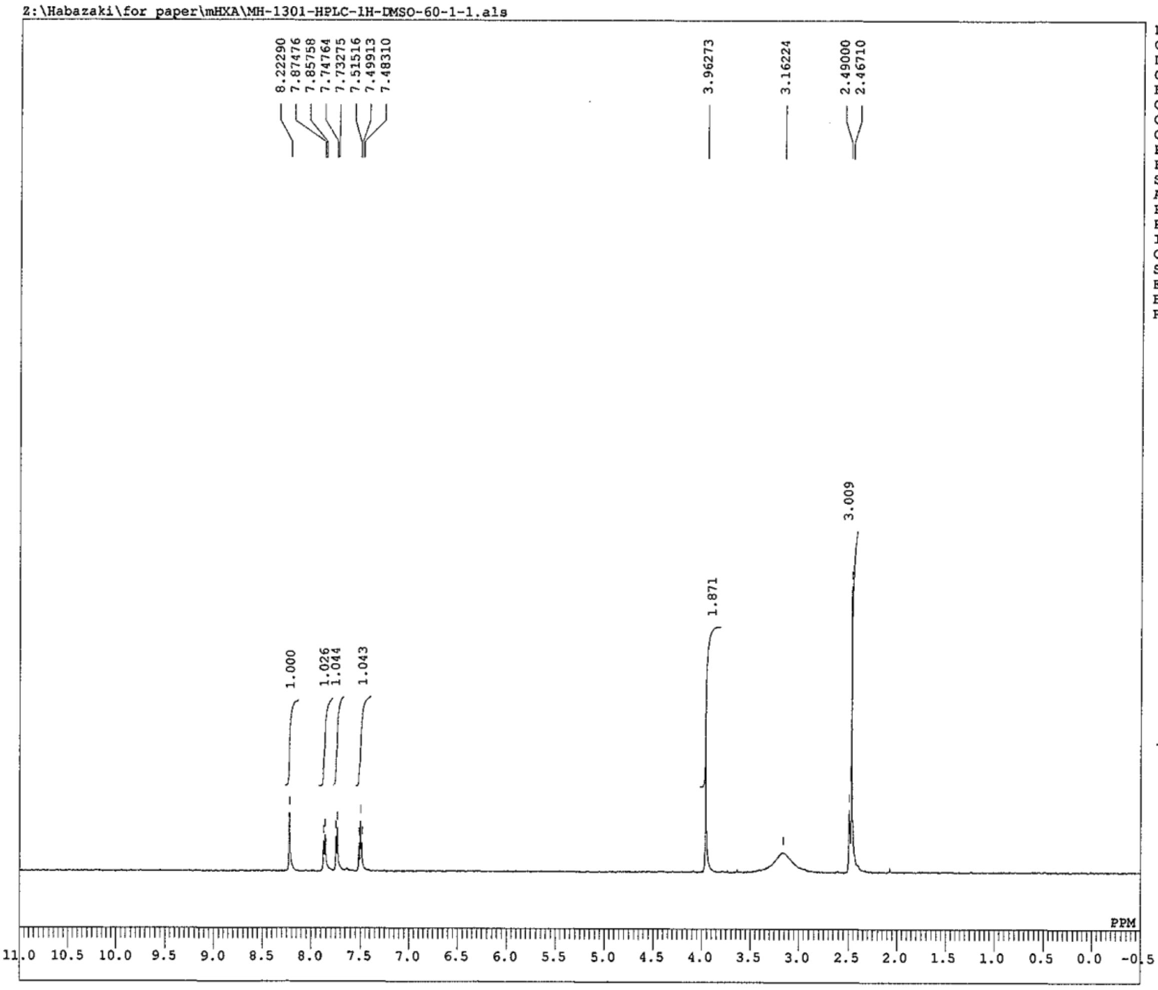

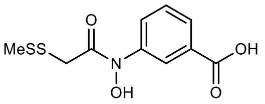

S22

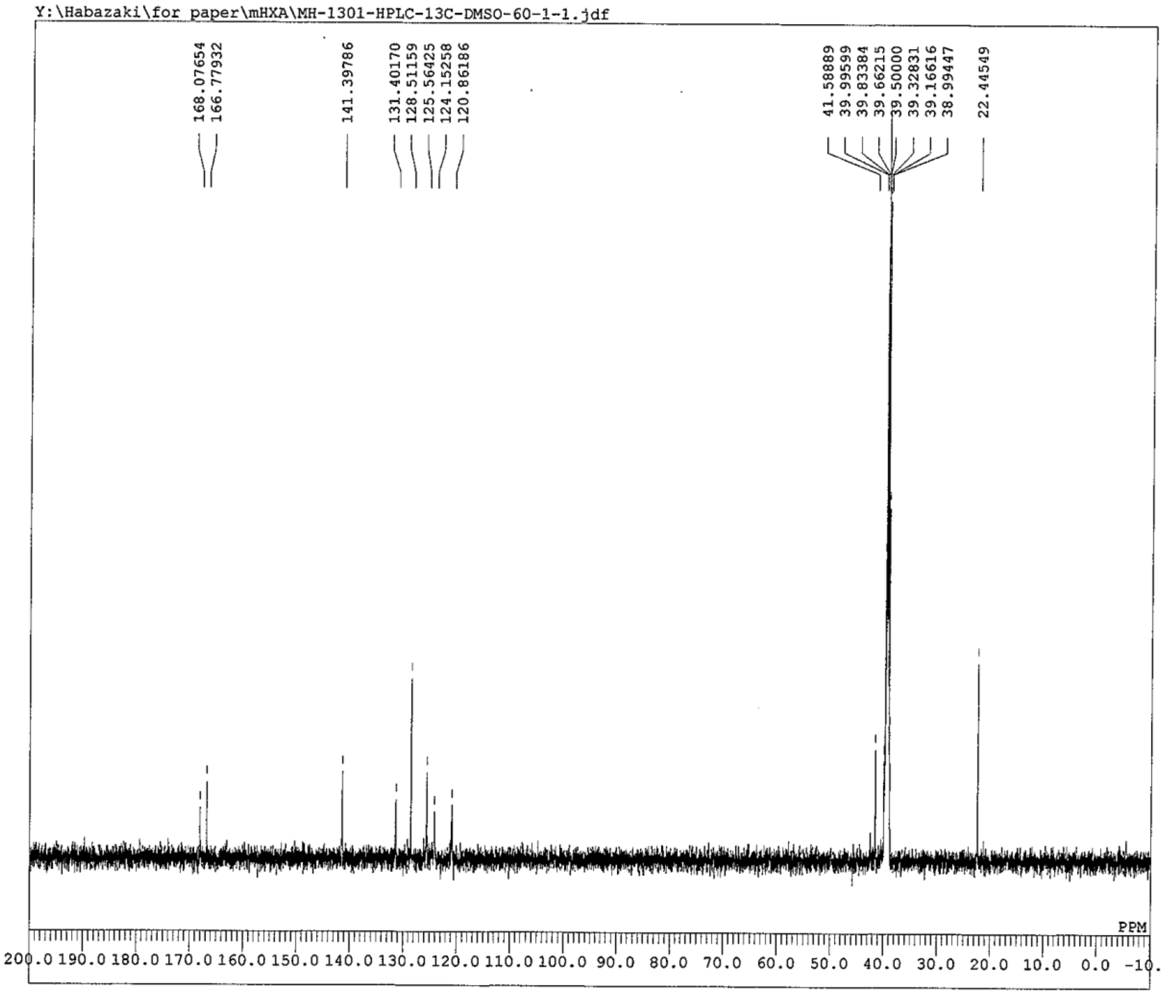

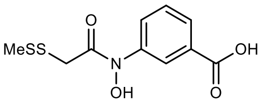

S22

Supplementary Spectral Data 11. ^{1}H and ^{13}C NMR spectra of compound 2

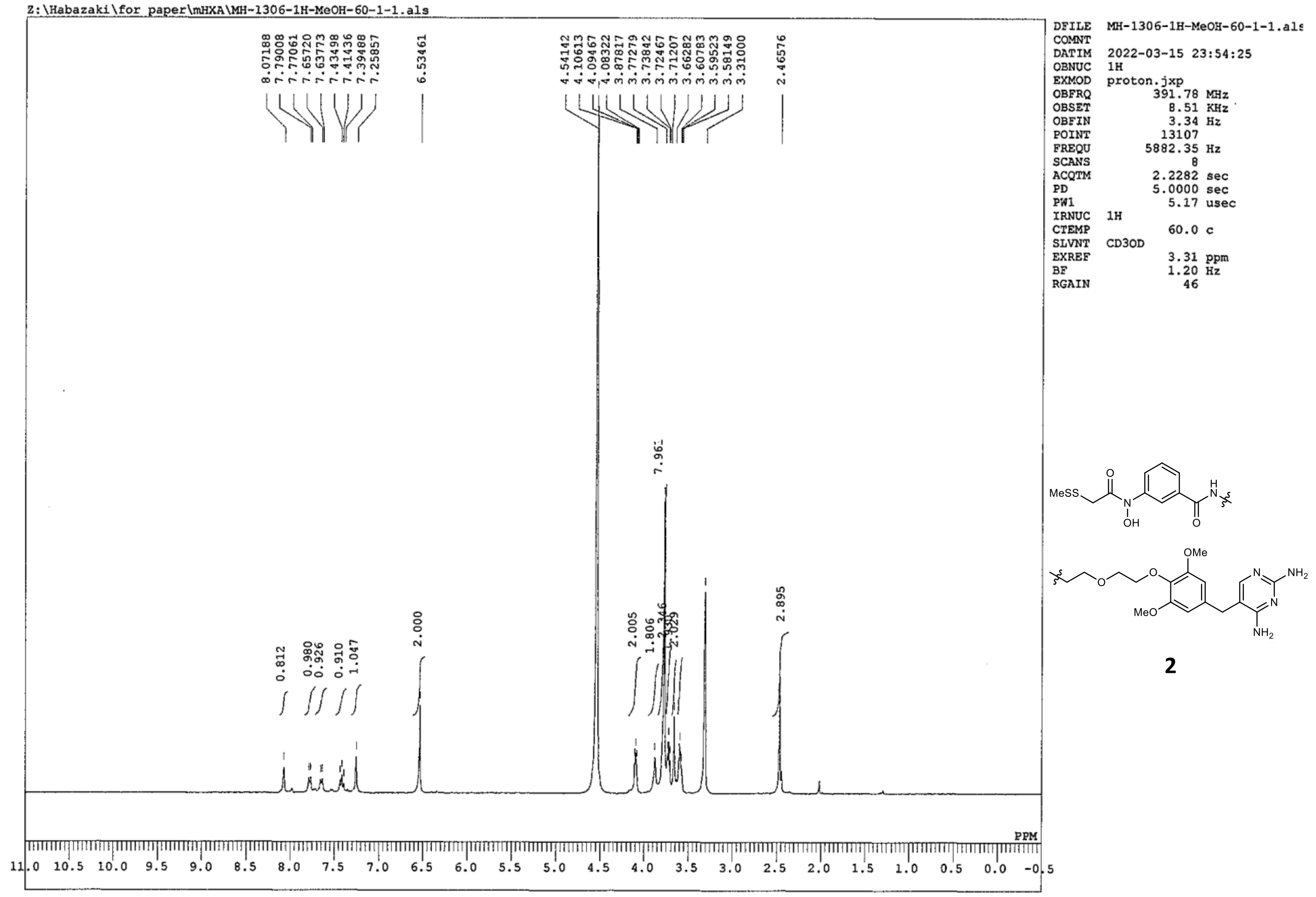

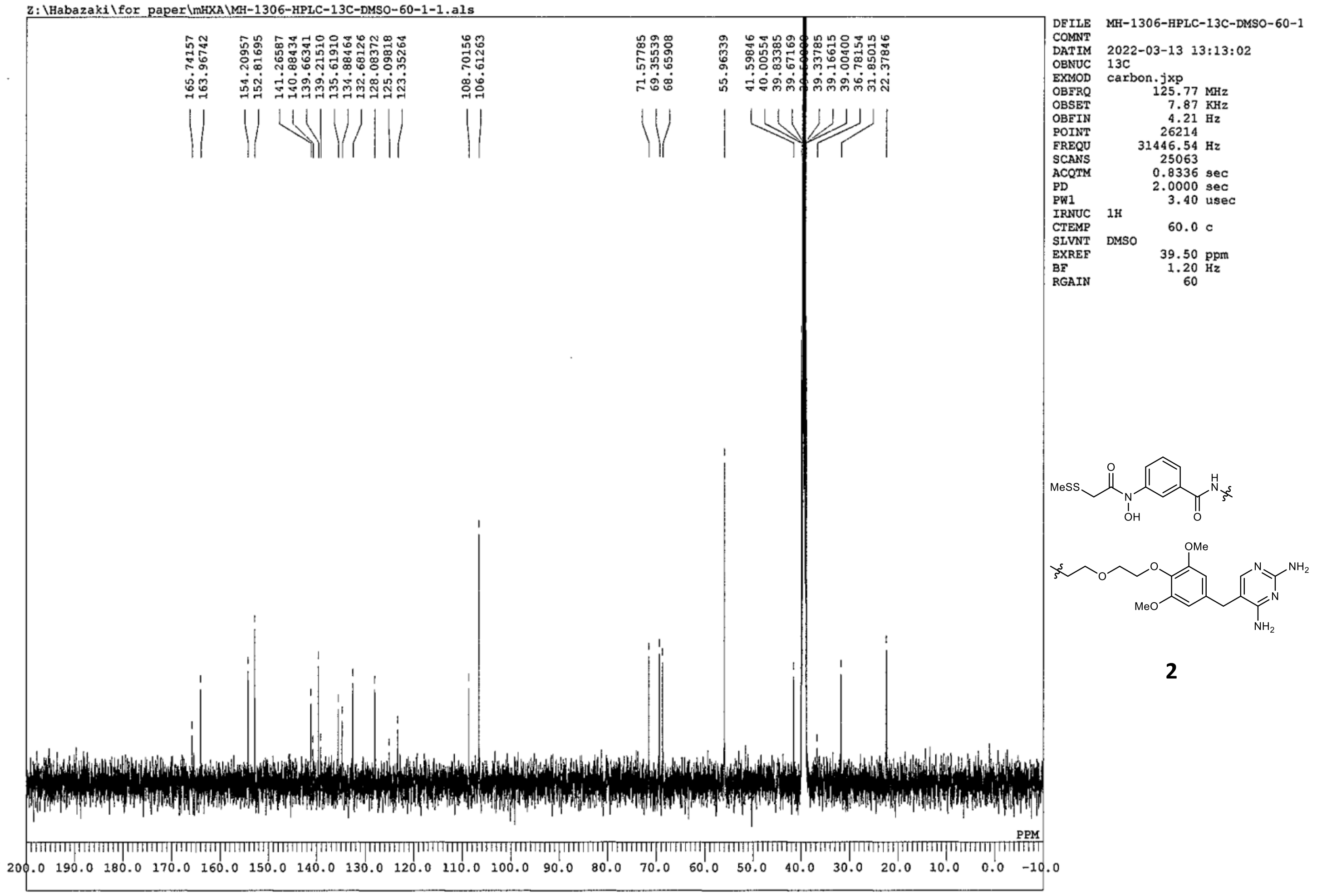

Supplementary Spectral Data 12. ^{1}H and ^{13}C NMR spectra of compound S23

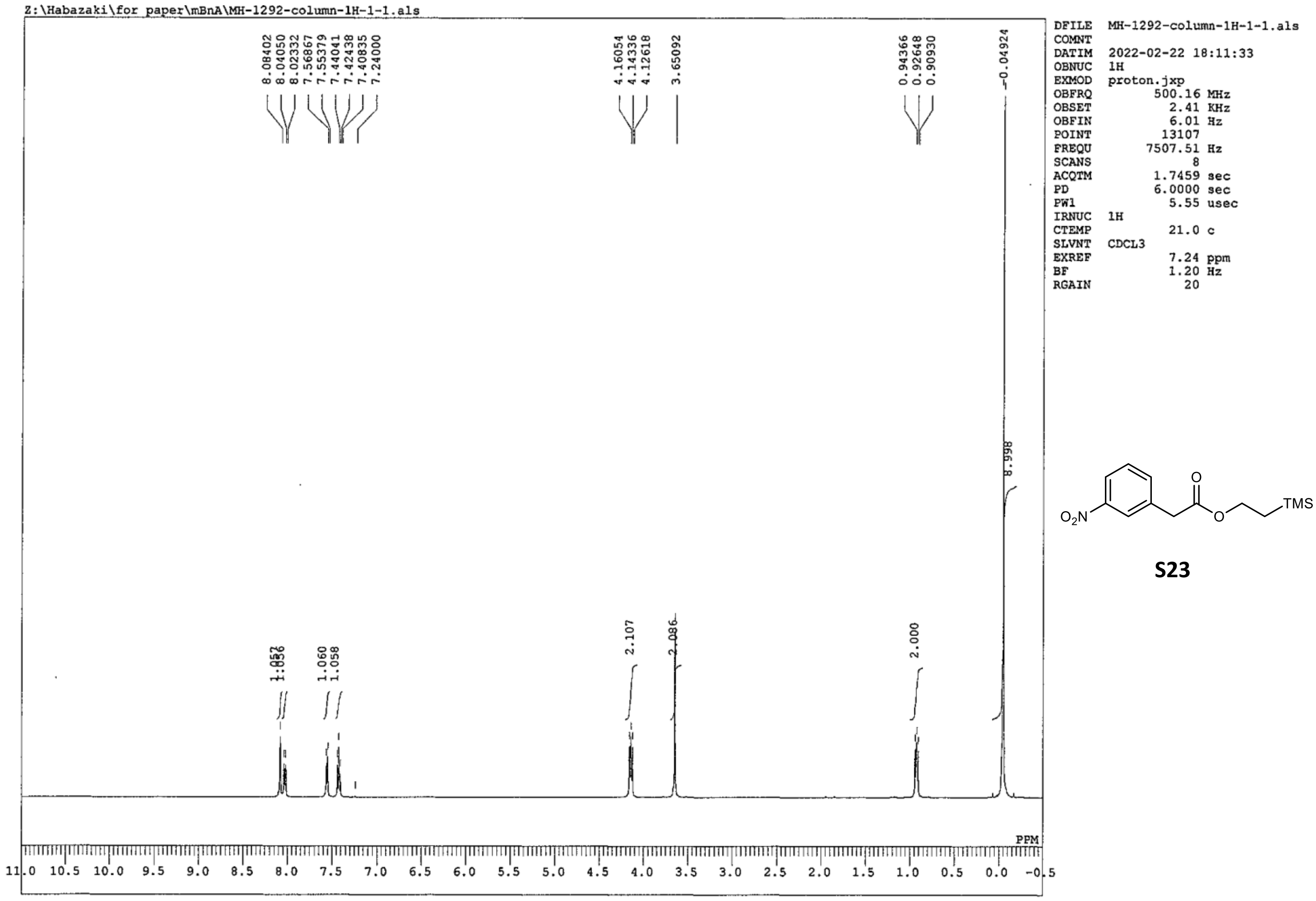

S23

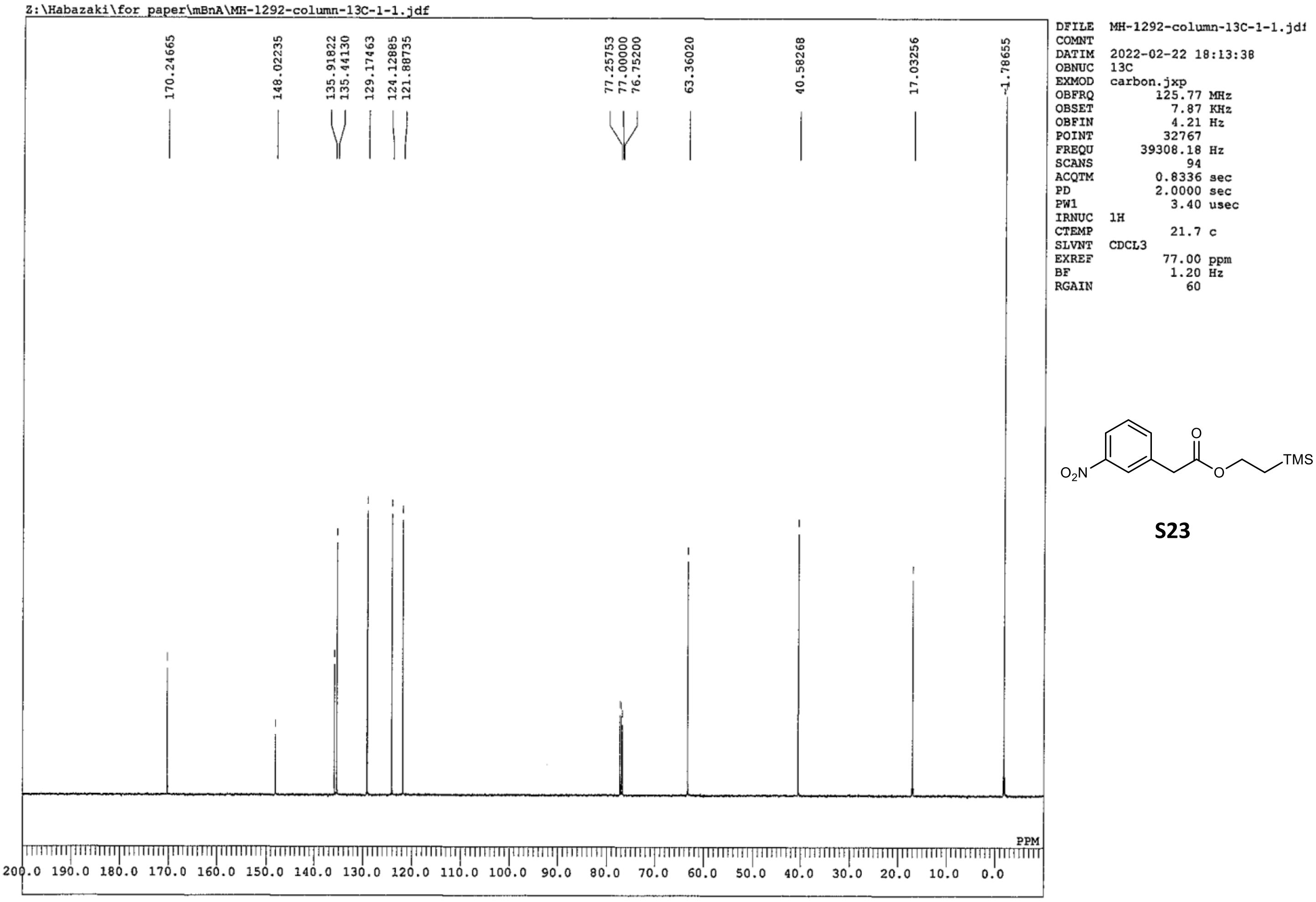

S23

Supplementary Spectral Data 13. ^{1}H and ^{13}C NMR spectra of compound S26

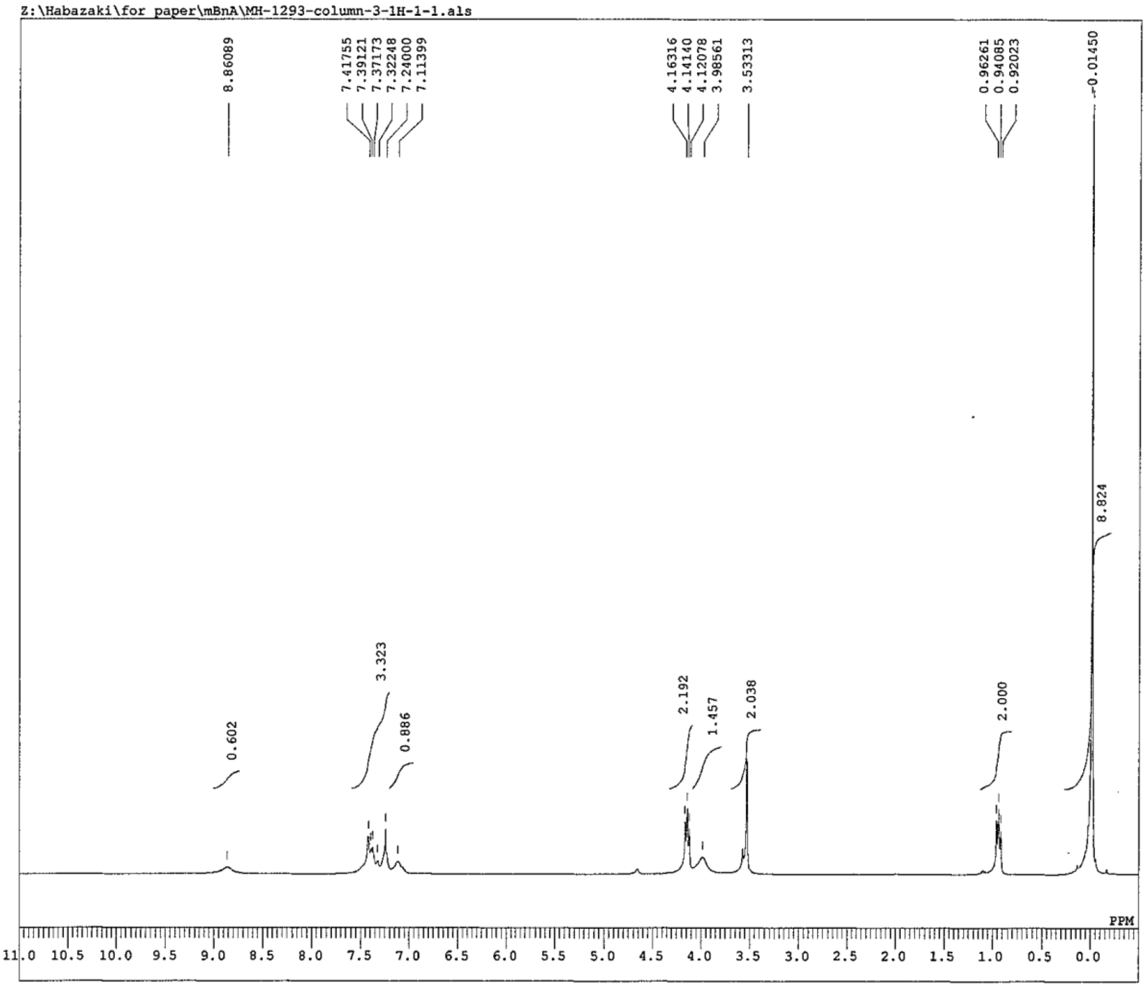

DFILE	MH-1293-column-3-1H-1-1.al
COMNT	
DATIM	2022-03-03 23:46:23
OBNUC	1H
EXMOD	proton.jxp
OBFRQ	391.78 MHz
OBSET	8.51 KHz
OBFIN	3.34 Hz
POINT	13107
FREQU	5882.35 Hz
SCANS	8
ACQTM	2.2282 sec
PD	5.0000 sec
PW1	5.17 usec
IRNUC	1H
CTEMP	60.0 c
SLVNT	CDCL3
EXREF	7.24 ppm
BF	1.20 Hz
RGAIN	22

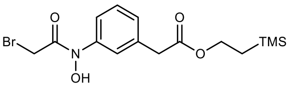

S26

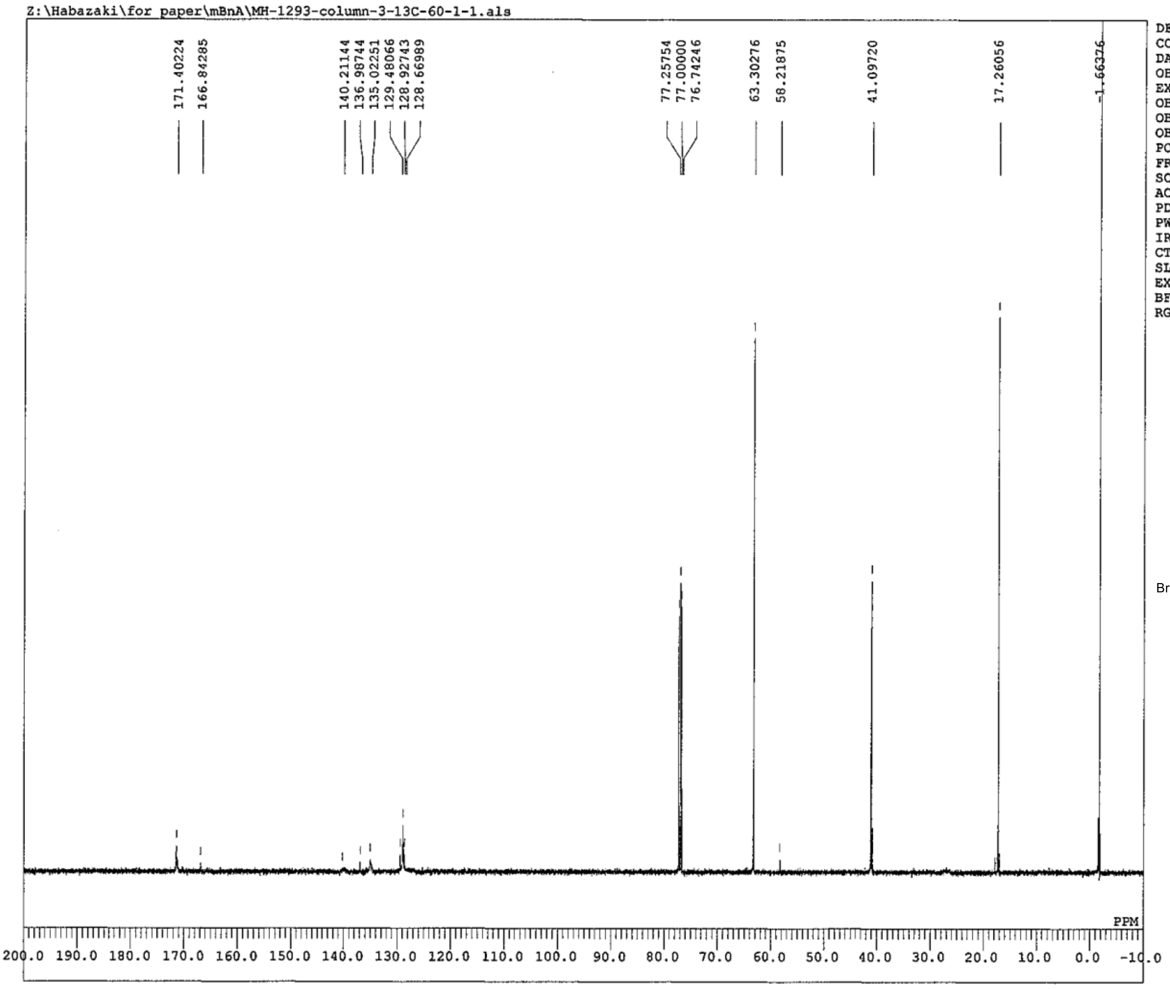

DFILE	MH-1293-column-3-13C-60-1-
COMNT	
DATIM	2022-03-04 19:24:51
OBNUC	13C
EXMOD	carbon.jxp
OBFRQ	125.77 MHz
OBSET	7.87 KHz
OBFIN	4.21 Hz
POINT	26214
FREQU	31446.54 Hz
SCANS	630
ACQTM	0.8336 sec
PD	2.0000 sec
PW1	3.40 usec
IRNUC	1H
CTEMP	60.0 c
SLVNT	CDCL3
EXREF	77.00 ppm
BF	0.12 Hz
RGAIN	60

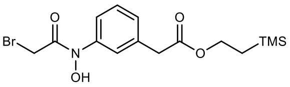

S26

Supplementary Spectral Data 14. ^{1}H and ^{13}C NMR spectra of compound S27

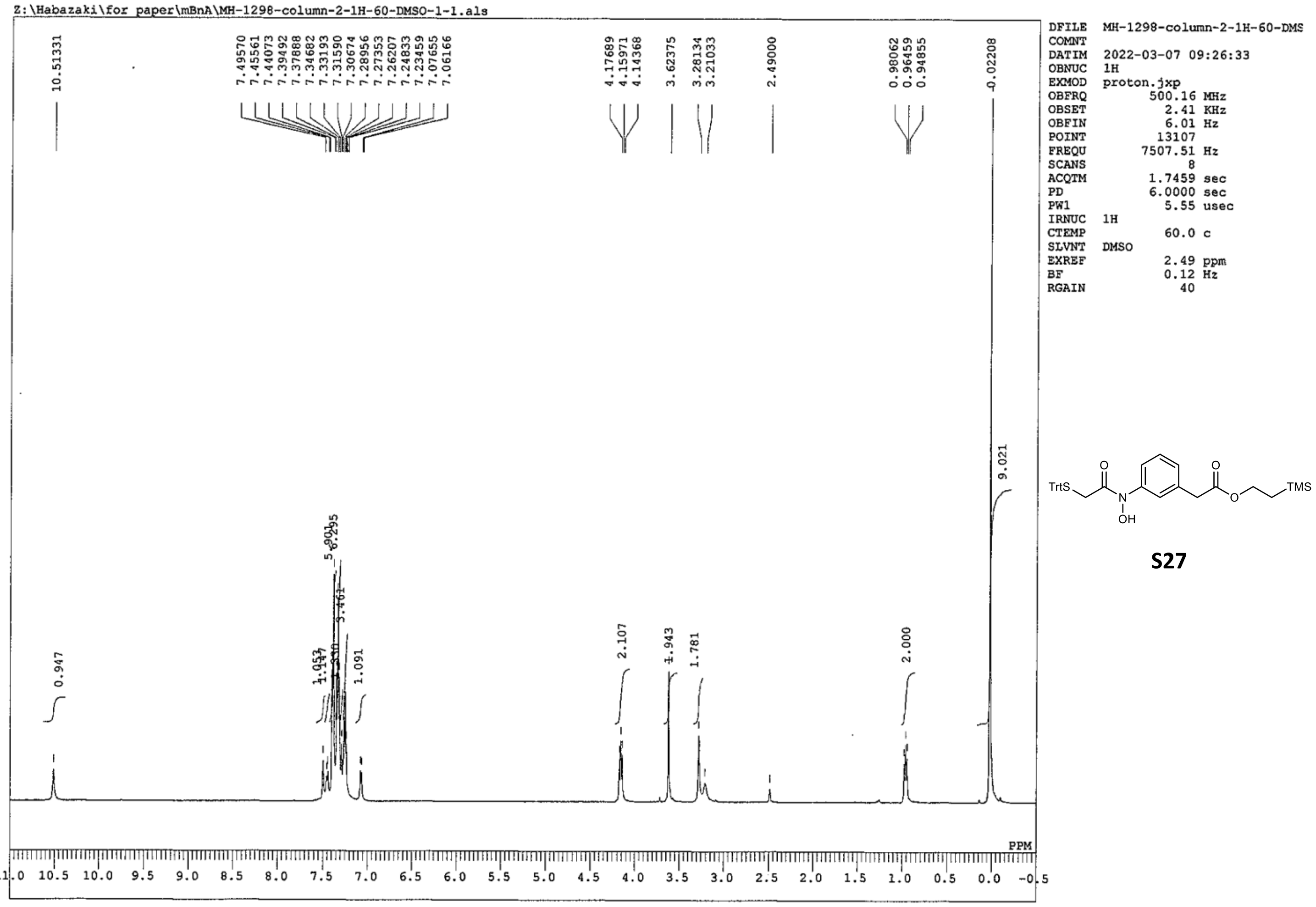

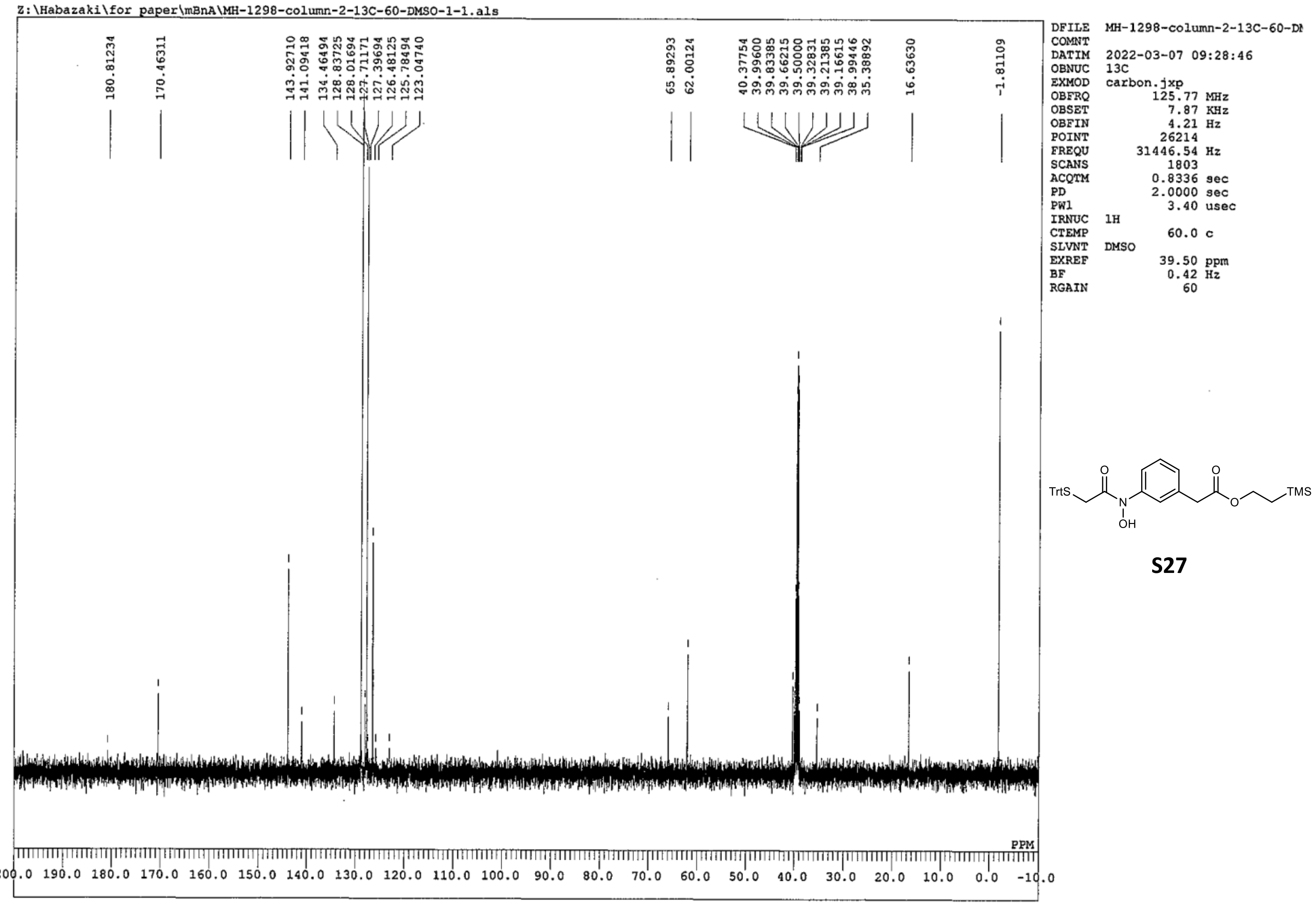

Supplementary Spectral Data 15. ^{1}H and ^{13}C NMR spectra of compound S29

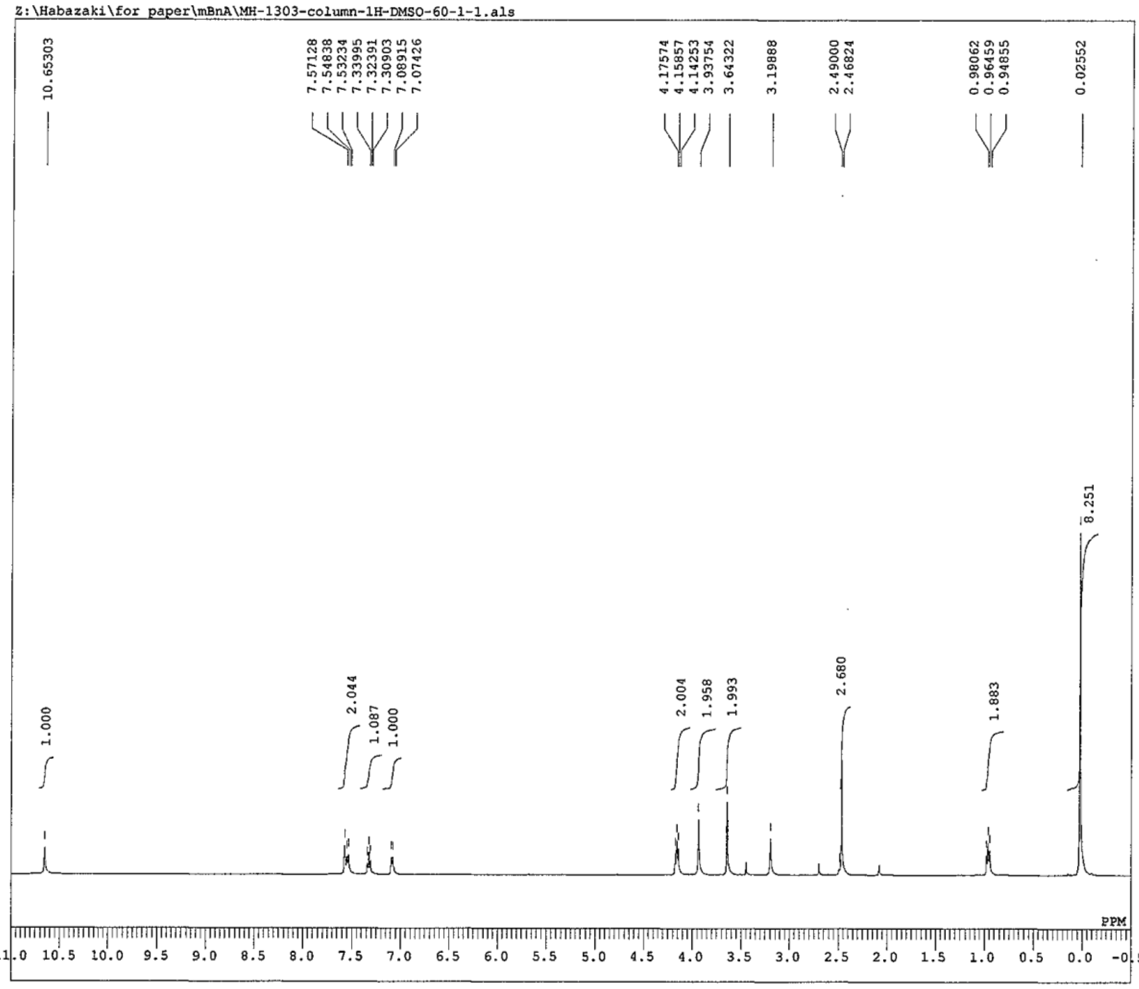

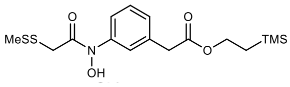

S29

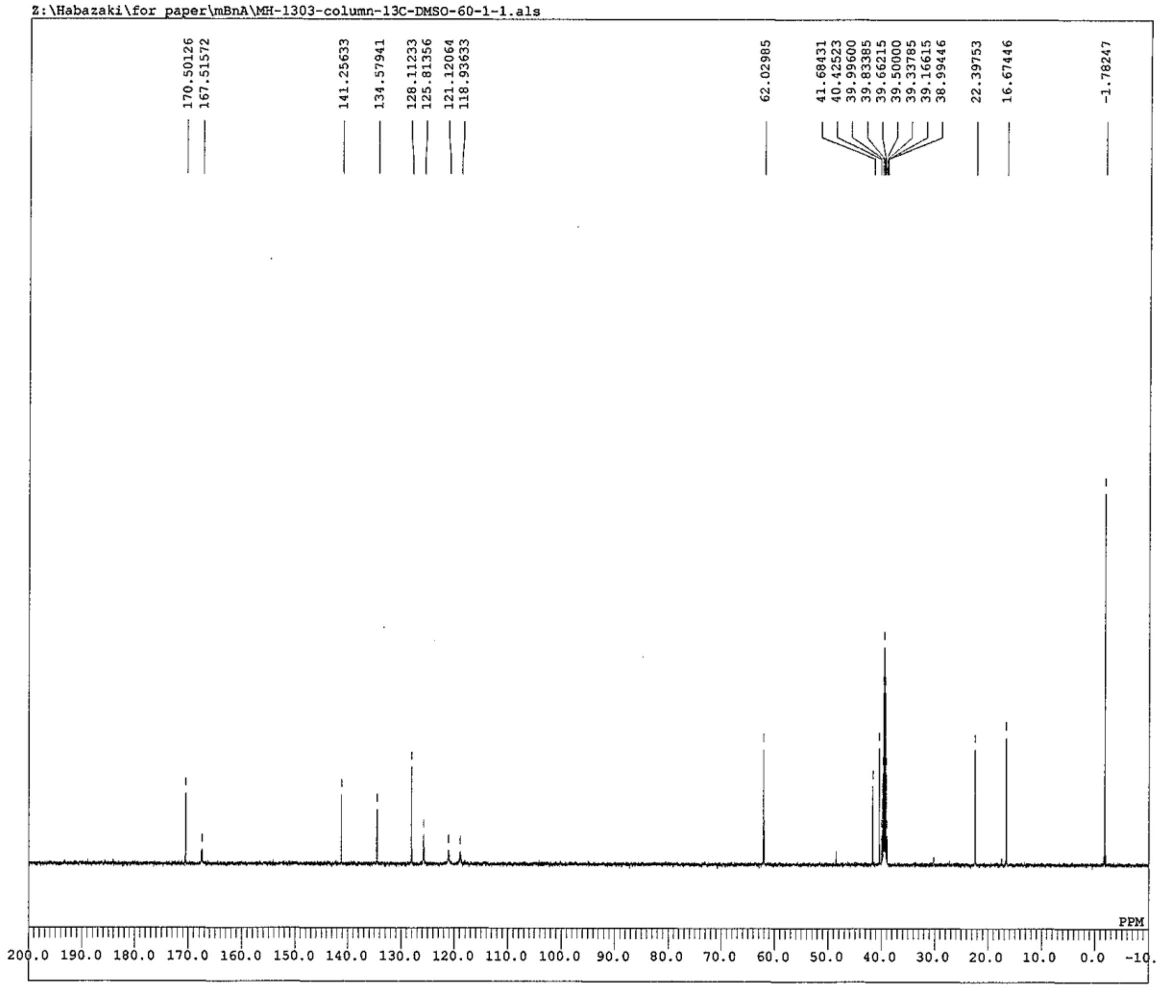

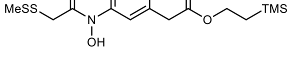

S29

Supplementary Spectral Data 16. ^{1}H and ^{13}C NMR spectra of compound S30

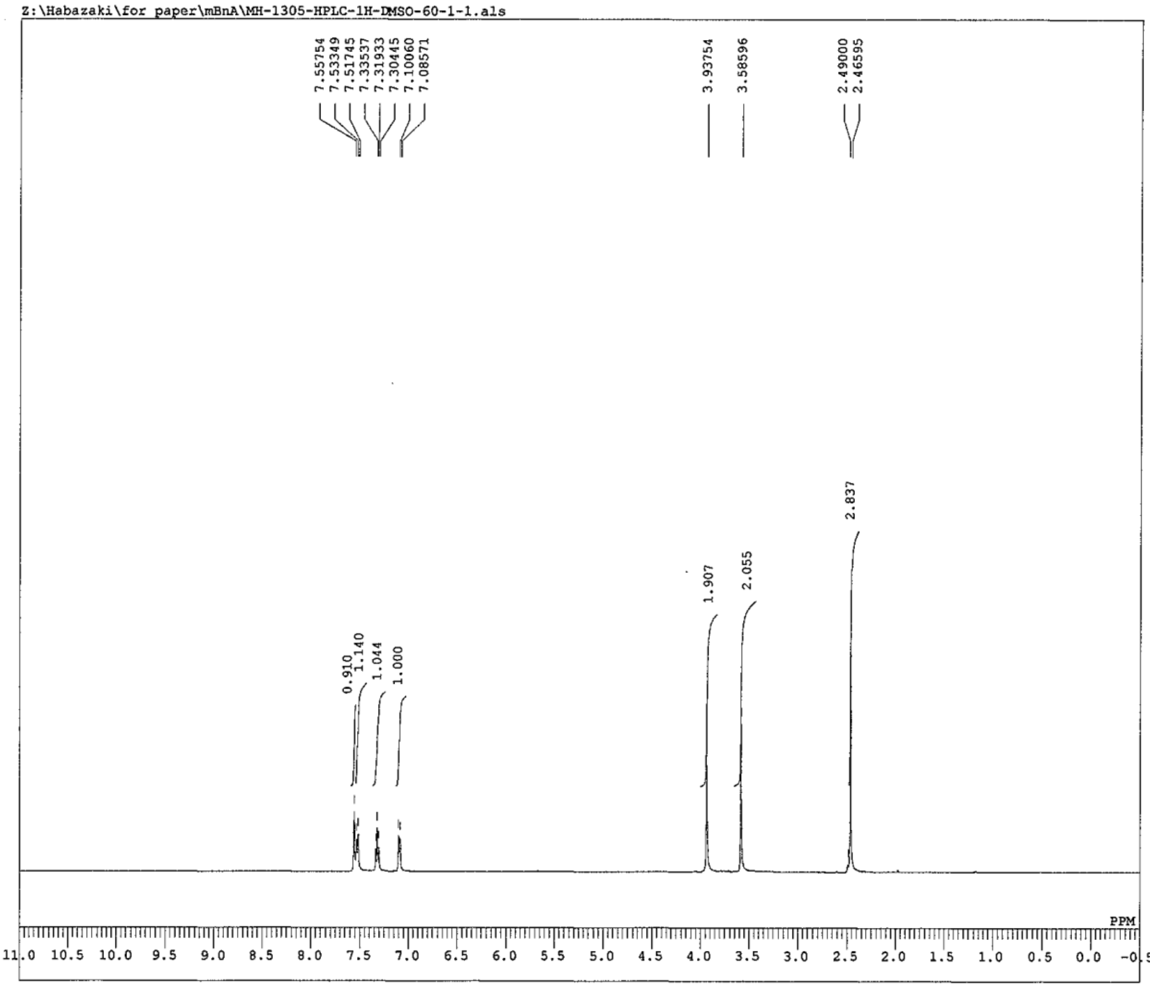

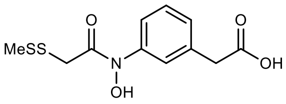

S30

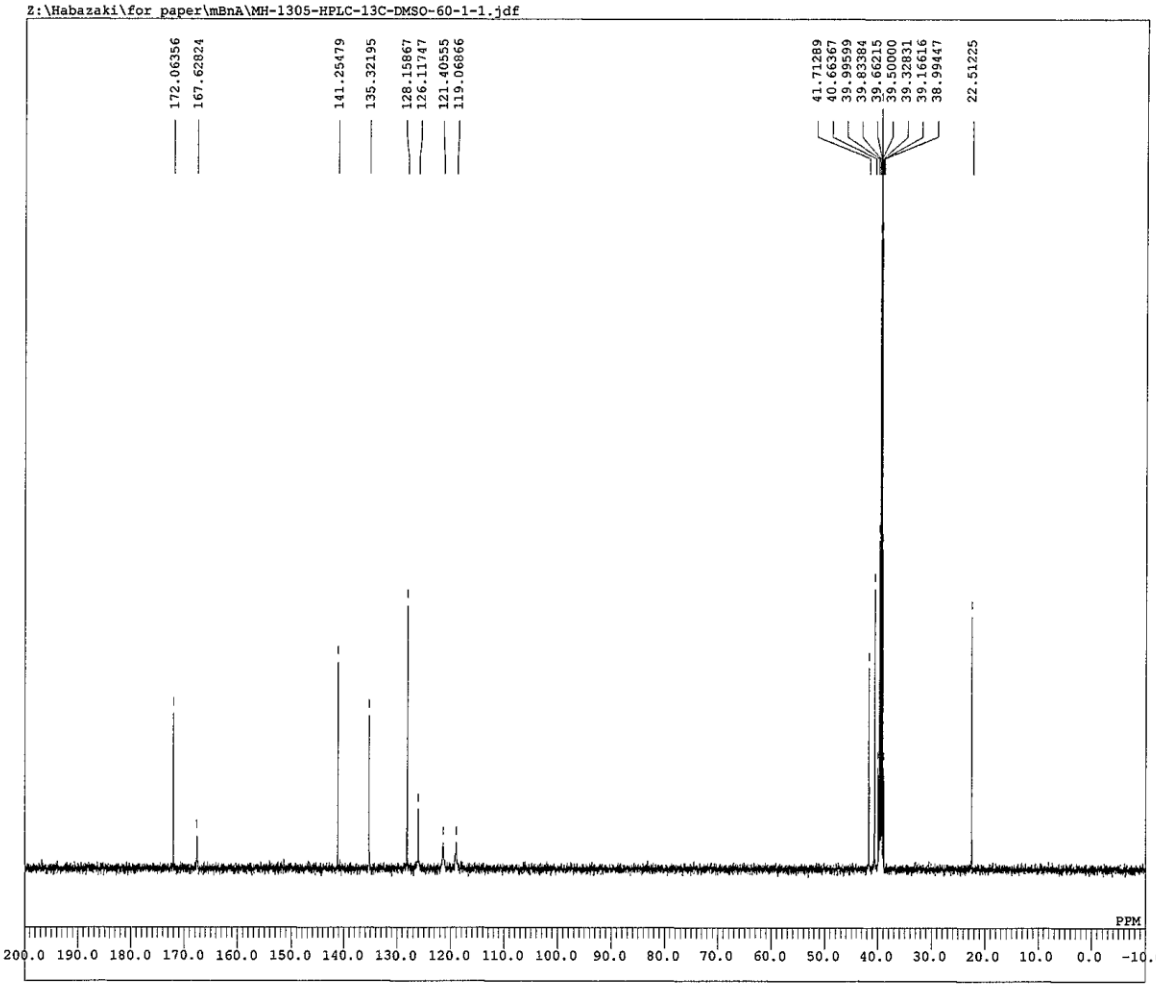

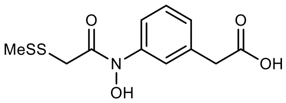

S30

Supplementary Spectral Data 17. ^{1}H and ^{13}C NMR spectra of compound 3

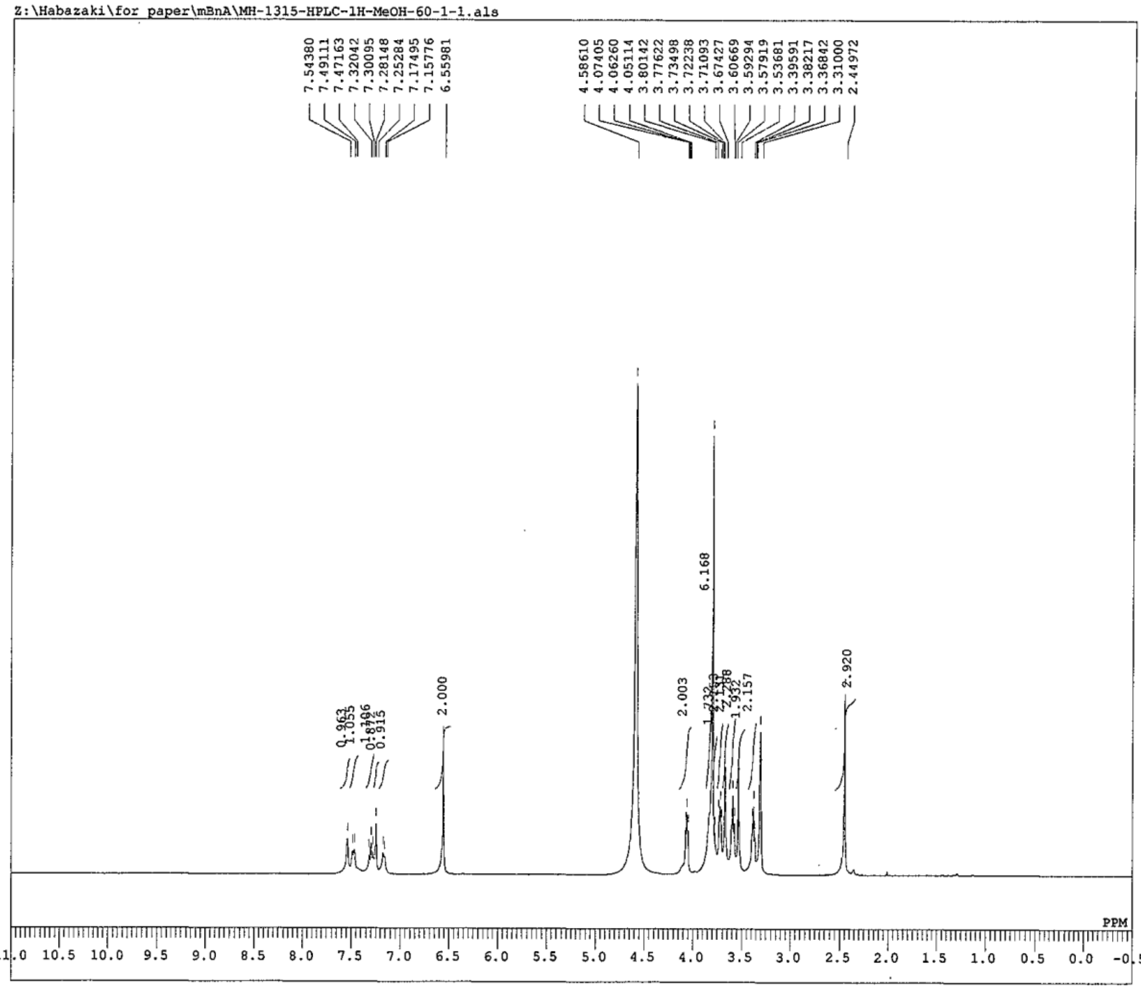

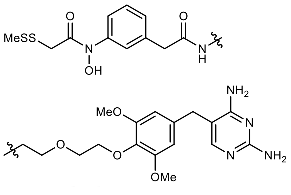

3

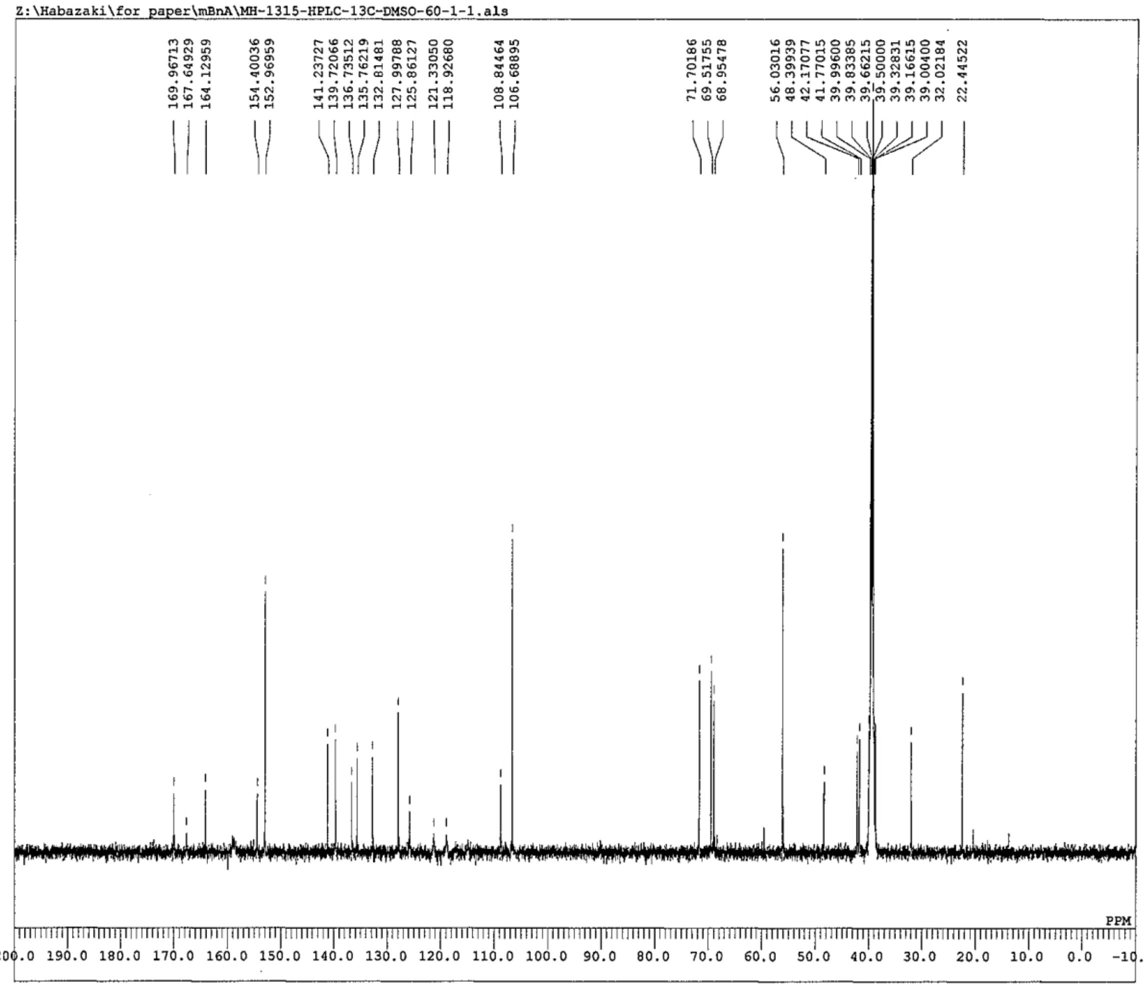

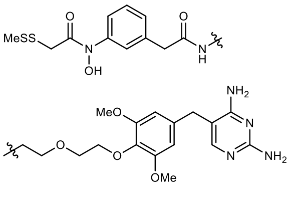

3

Supplementary Spectral Data 18. ^{1}H and ^{13}C NMR spectra of compound 4

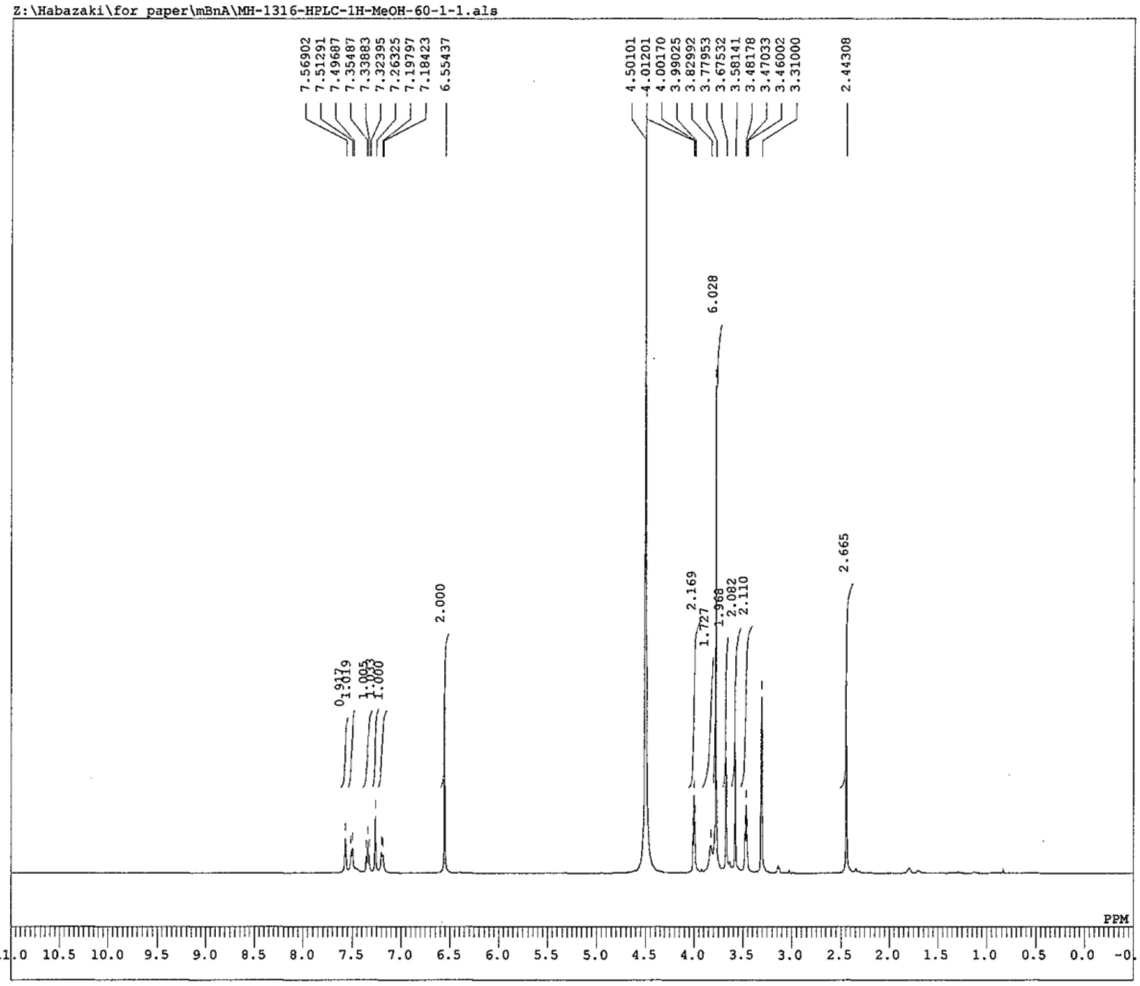

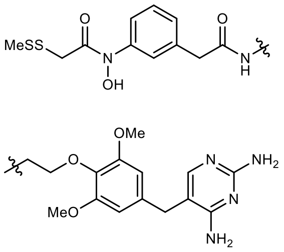

4

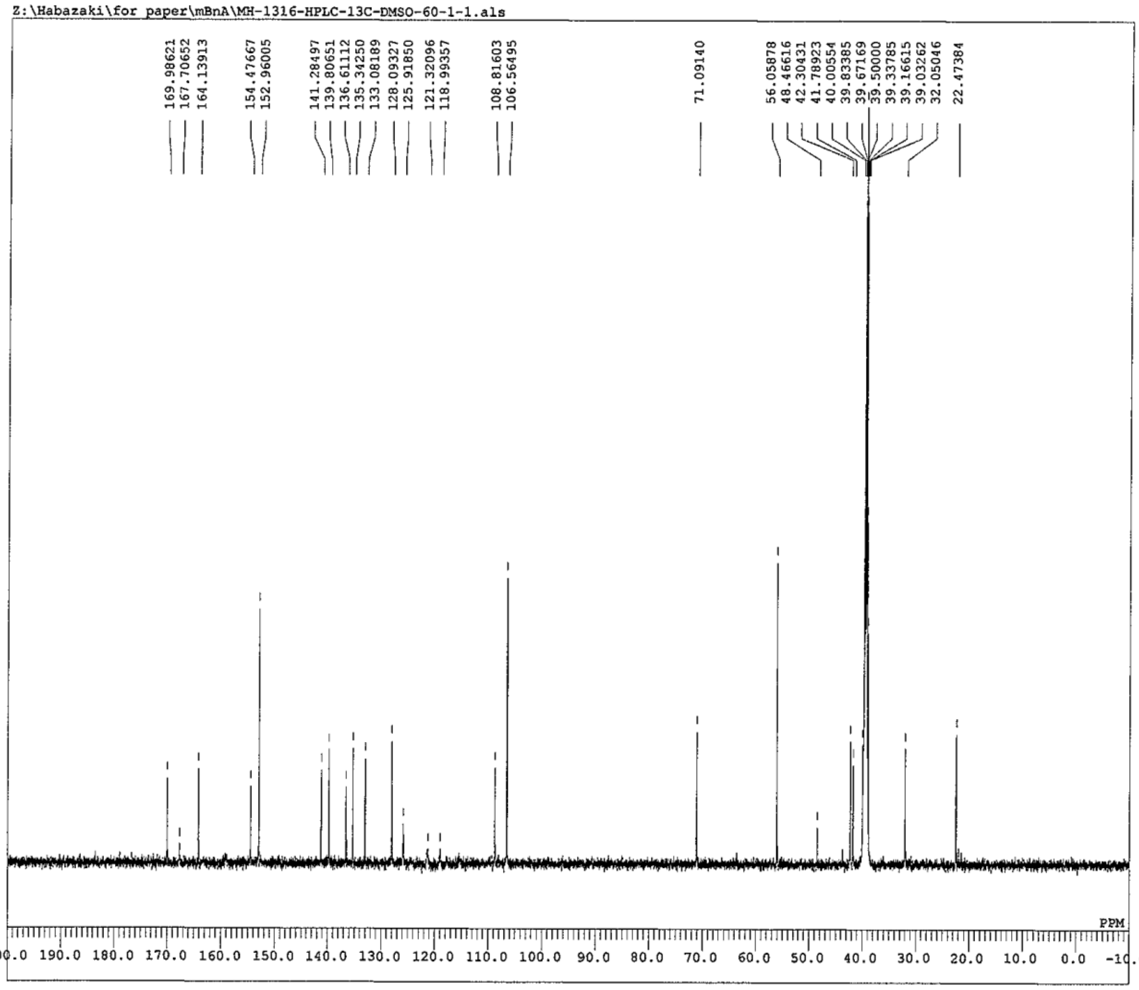

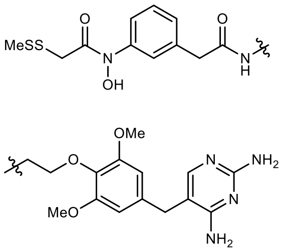

4

Supplementary Spectral Data 19. ^{1}H and ^{13}C NMR spectra of compound S32

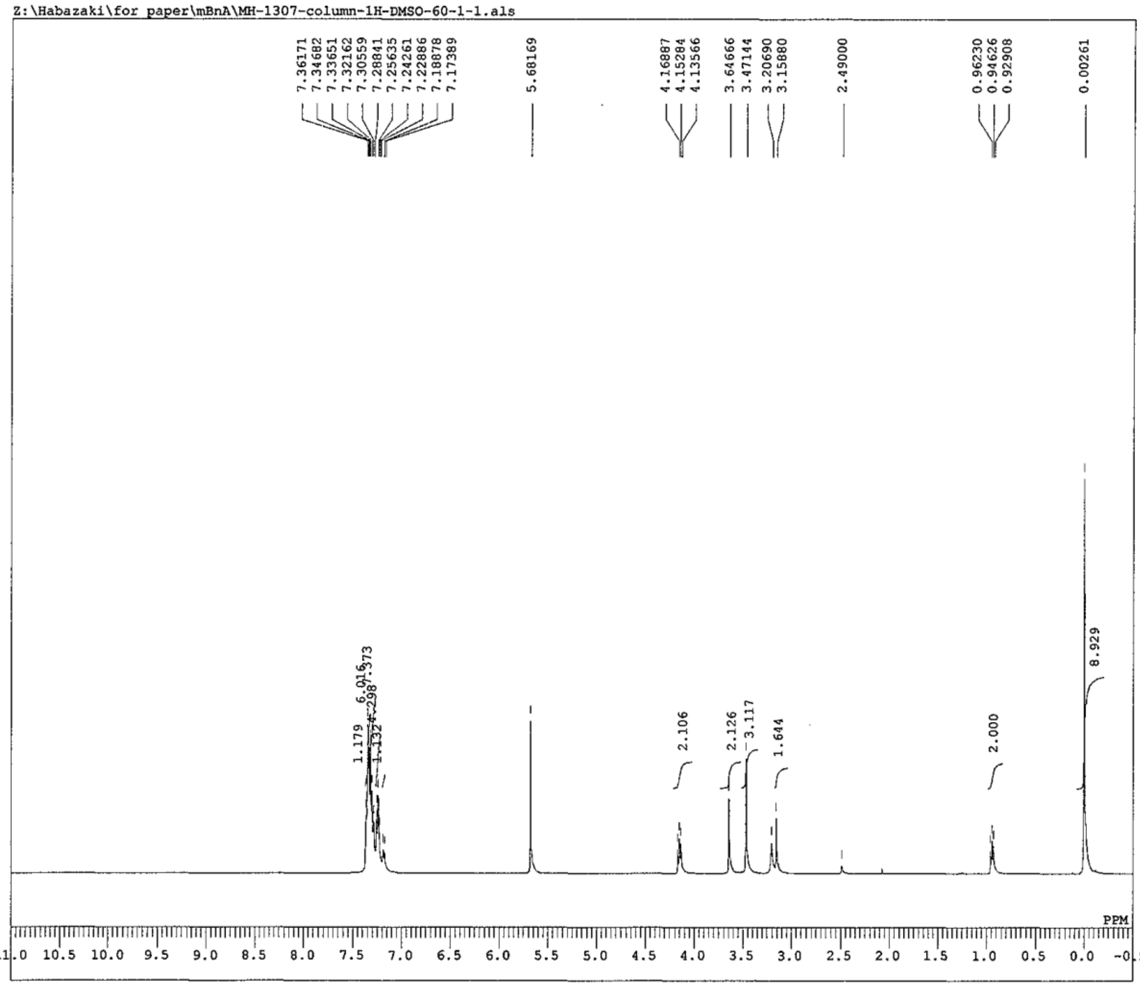

DFILE MH-1307-column-1H-DMSO-60-
COMNT
DATIM 2022-03-14 23:21:57
OBNUC 1H
EXMOD proton.jxp
OBFRQ 500.16 MHz
OBSET 2.41 KHz
OBFIN 6.01 Hz
POINT 13107
FREQU 7507.51 Hz
SCANS 8
ACQTM 1.7459 sec
PD 6.0000 sec
PW1 5.55 usec
IRNUC 1H
CTEMP 60.0 c
SLVNT DMSO
EXREF 2.49 ppm
BF 1.20 Hz
RGAIN 38

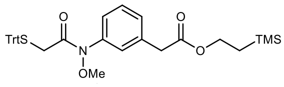

S32

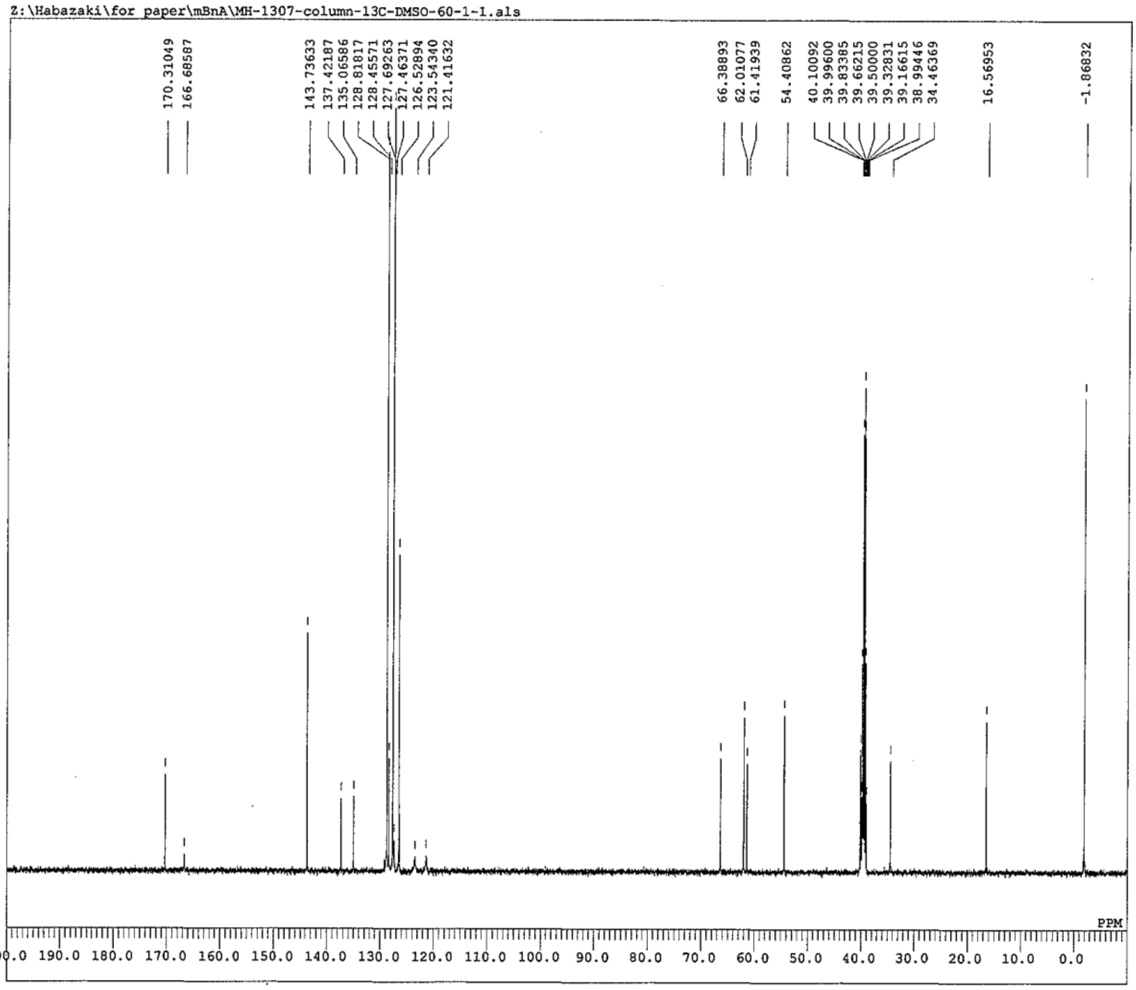

DFILE MH-1307-column-13C-DMSO-6(
COMNT
DATIM 2022-03-14 23:24:00
OBNUC 13C
EXMOD carbon.jxp
OBFRQ 125.77 MHz
OBSET 7.87 KHz
OBFIN 4.21 Hz
POINT 26214
FREQU 31446.54 Hz
SCANS 11873
ACQTM 0.8336 sec
PD 2.0000 sec
PW1 3.40 usec
IRNUC 1H
CTEMP 60.0 c
SLVNT DMSO
EXREF 39.50 ppm
BF 1.20 Hz
RGAIN 60

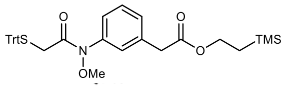

S32

Supplementary Spectral Data 20. ^{1}H and ^{13}C NMR spectra of compound S34

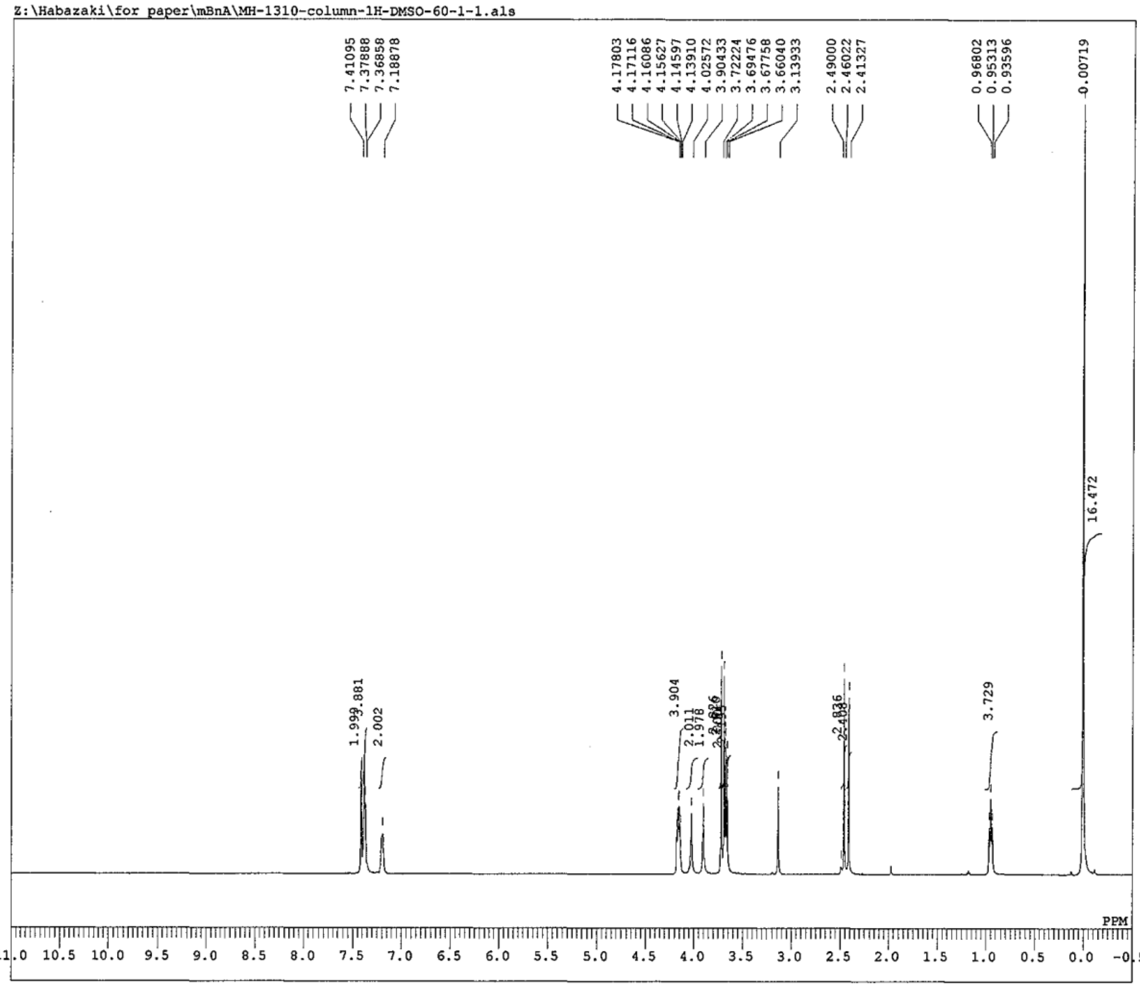

DFILE MH-1310-column-1H-DMSO-60-
COMNT
DATIM 2022-03-22 22:13:21
OBNUC 1H
EXMOD proton.jxp
OBFRQ 500.16 MHz
OBSET 2.41 KHz
OBFIN 6.01 Hz
POINT 13107
FREQU 7507.51 Hz
SCANS 8
ACQTM 1.7459 sec
PD 6.0000 sec
PW1 5.55 usec
IRNUC 1H
CTEMP 60.0 c
SLVNT DMSO
EXREF 2.49 ppm
BF 1.20 Hz
RGAIN 40

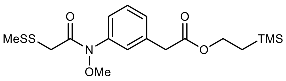

S34

* A ca. 1:1 mixture of *cis-trans* isomers at hydroxamic acid

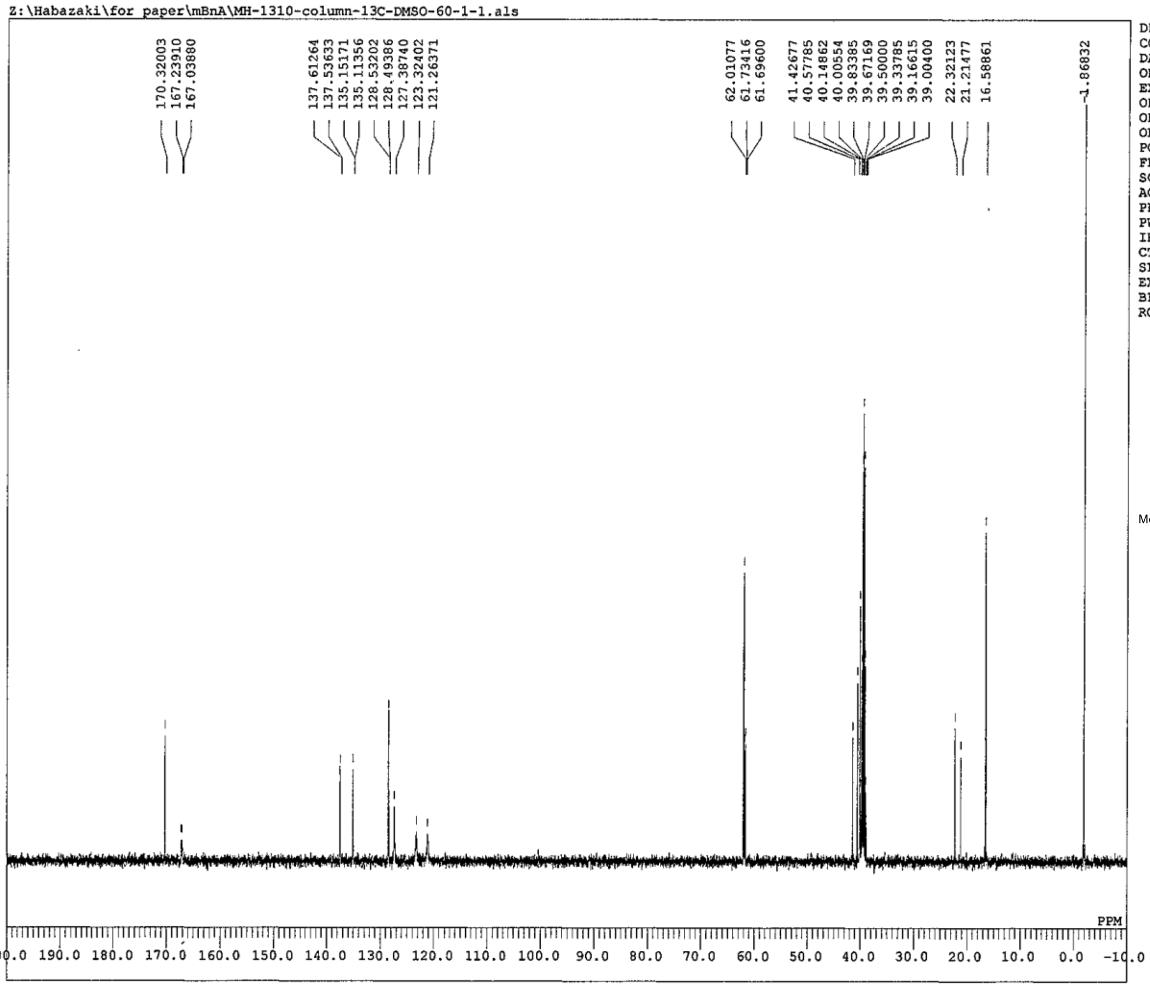

DFILE MH-1310-column-13C-DMSO-60
COMNT
DATIM 2022-03-22 22:15:55
OBNUC 13C
EXMOD carbon.jxp
OBFRQ 125.77 MHz
OBSET 7.87 KHz
OBFIN 4.21 Hz
POINT 26214
FREQU 31446.54 Hz
SCANS 13514
ACQTM 0.8336 sec
PD 2.0000 sec
PW1 3.40 usec
IRNUC 1H
CTEMP 60.0 c
SLVNT DMSO
EXREF 39.50 ppm
BF 1.20 Hz
RGAIN 60

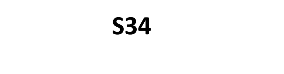

S34

* A ca. 1:1 mixture of *cis-trans* isomers at hydroxamic acid

Supplementary Spectral Data 21. ^{1}H and ^{13}C NMR spectra of compound S35

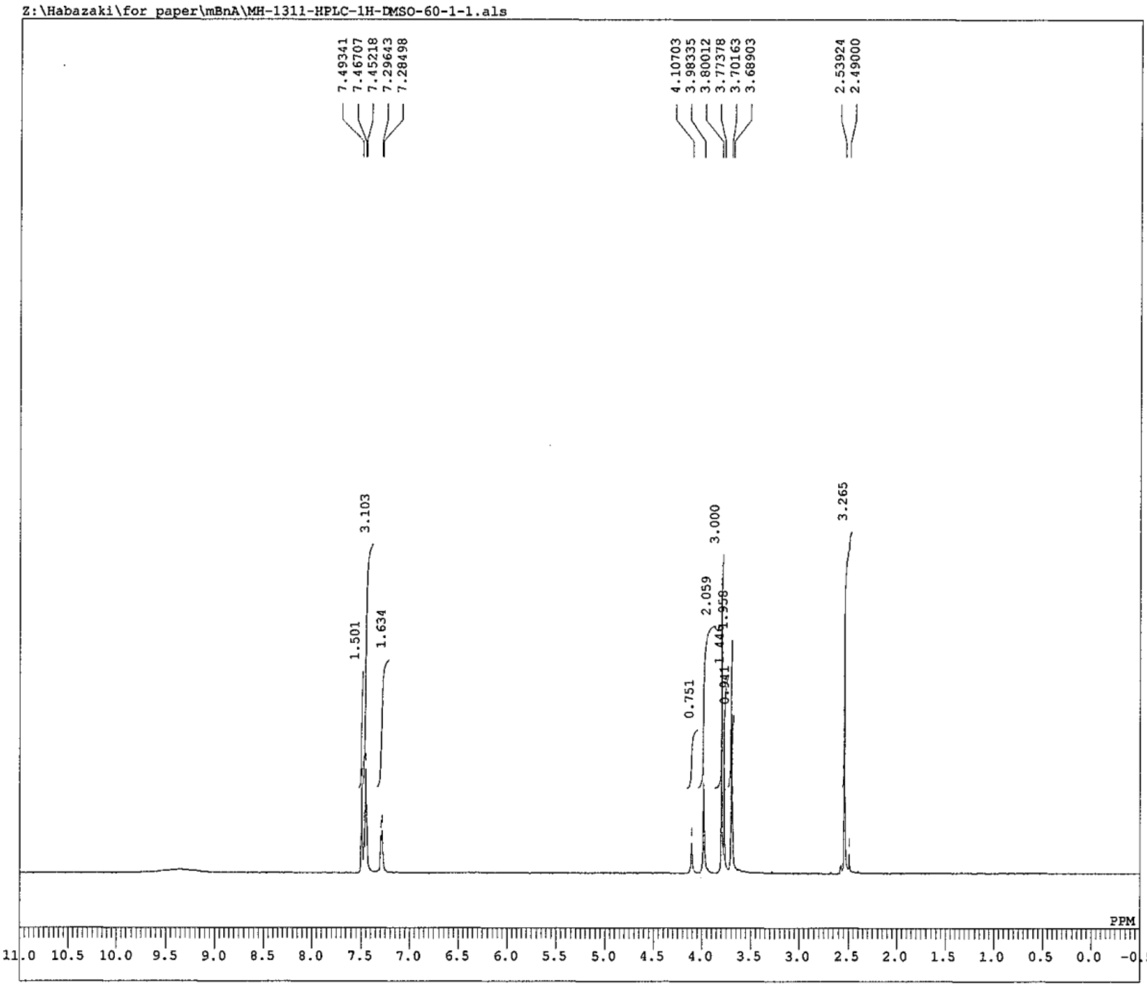

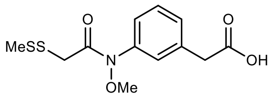

S35

* A ca. 2:1 mixture of *cis-trans* isomers at hydroxamic acid

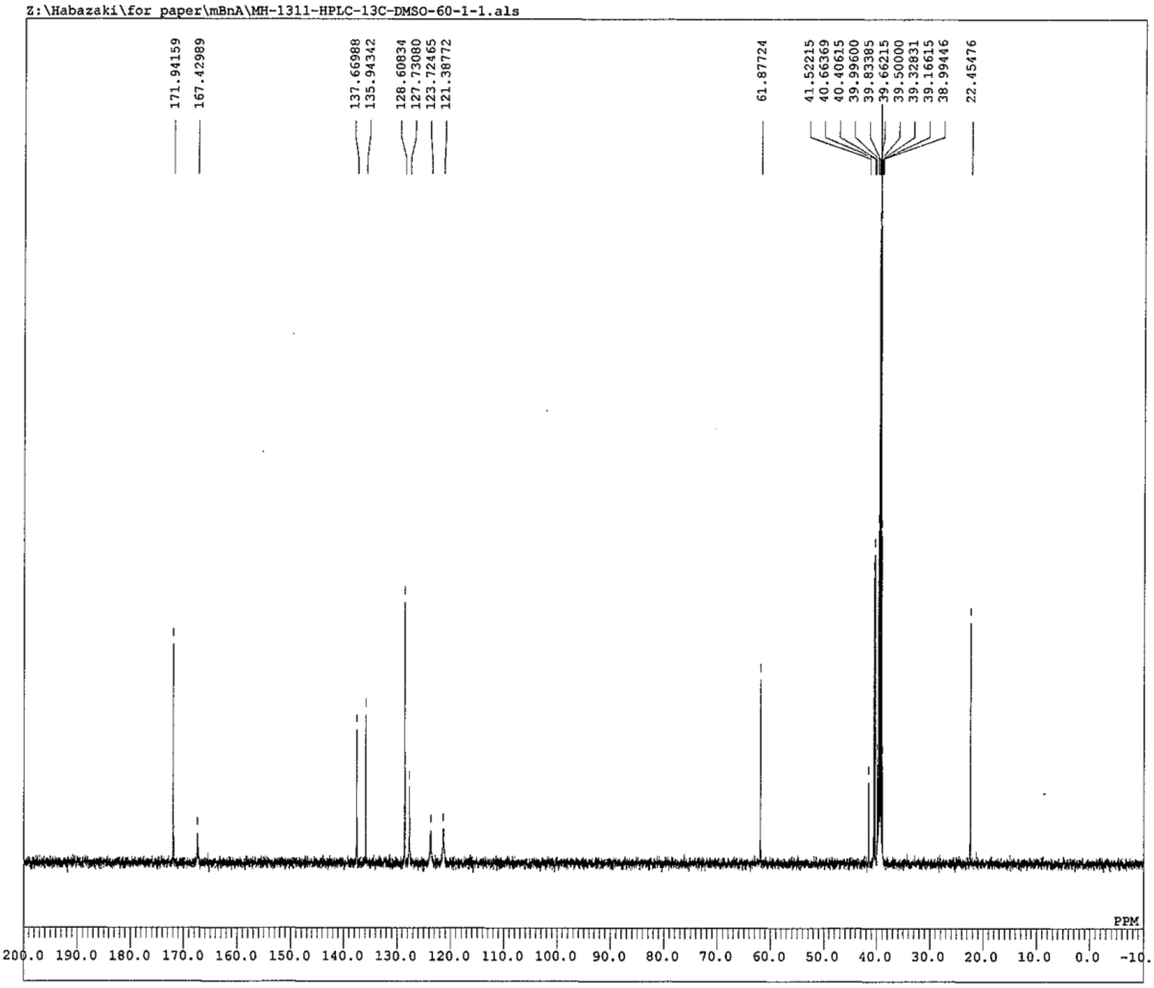

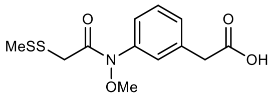

S35

* A ca. 2:1 mixture of *cis-trans* isomers at hydroxamic acid

Supplementary Spectral Data 22. ^{1}H and ^{13}C NMR spectra of compound 5

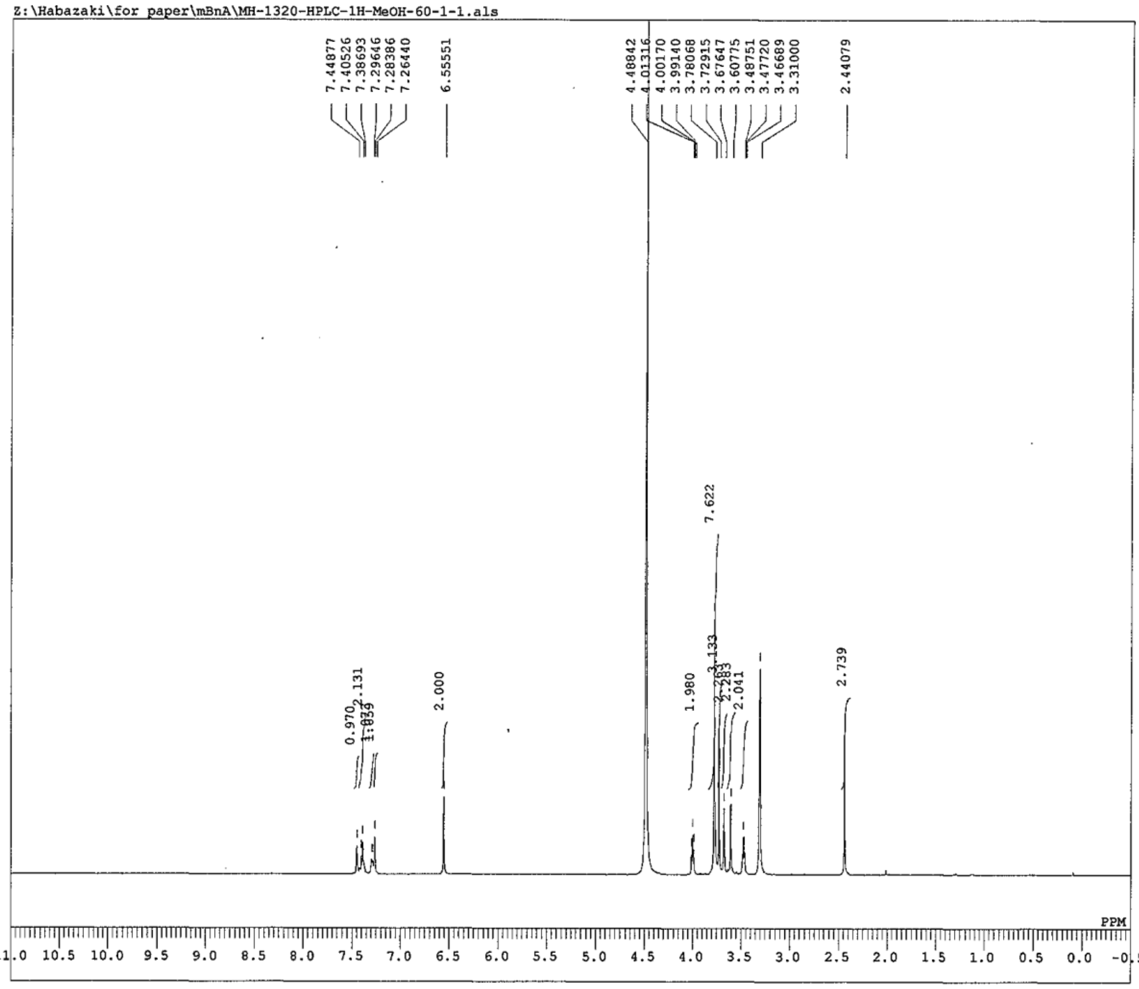

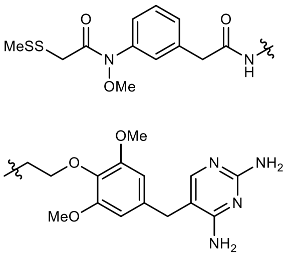

5

* A ca. 3:1 mixture of *cis-trans* isomers at hydroxamic acid

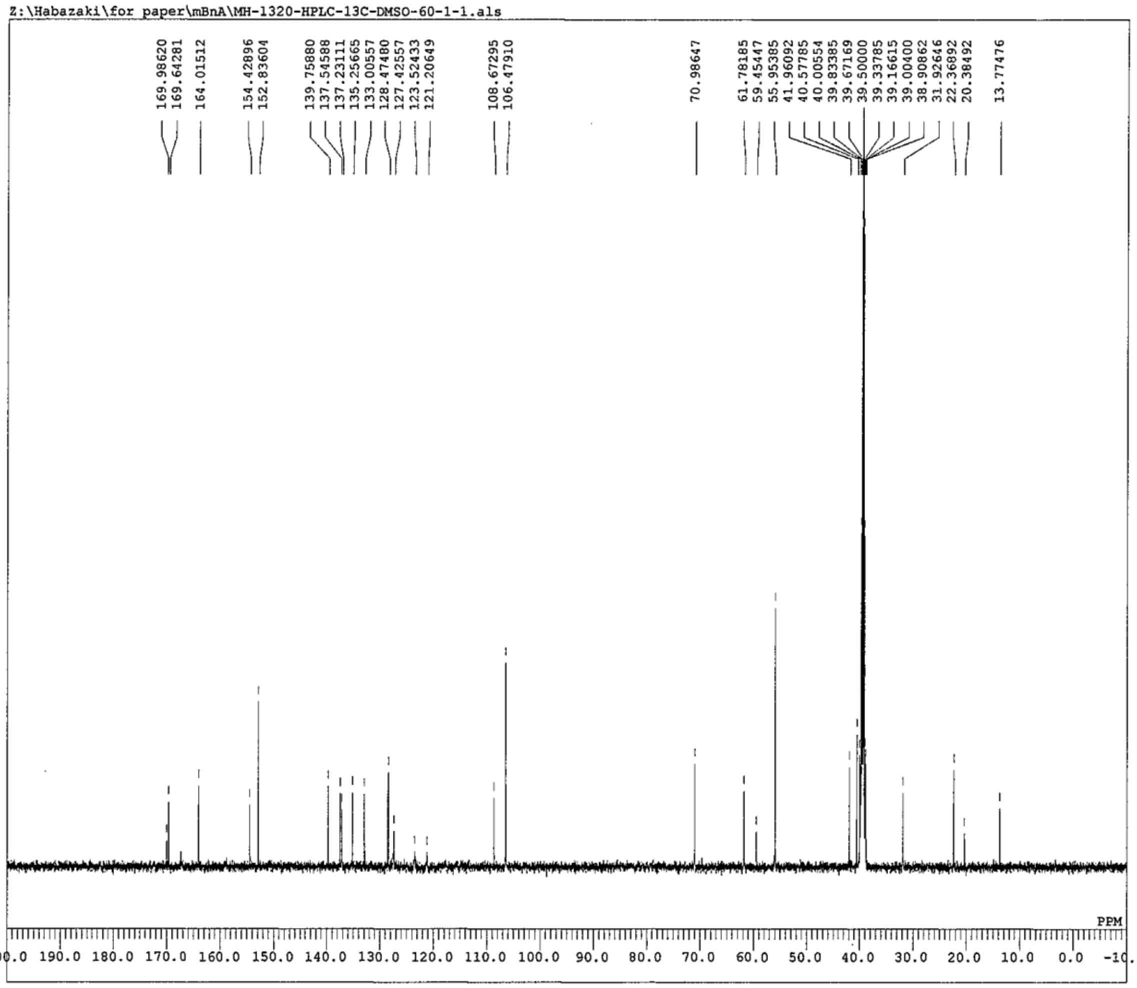

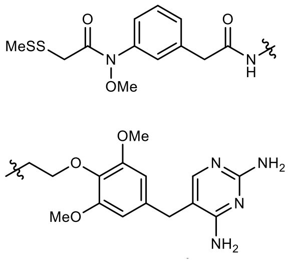

5

* A ca. 3:1 mixture of *cis-trans* isomers at hydroxamic acid

Supplementary Spectral Data 23. ^{1}H and ^{13}C NMR spectra of compound S36

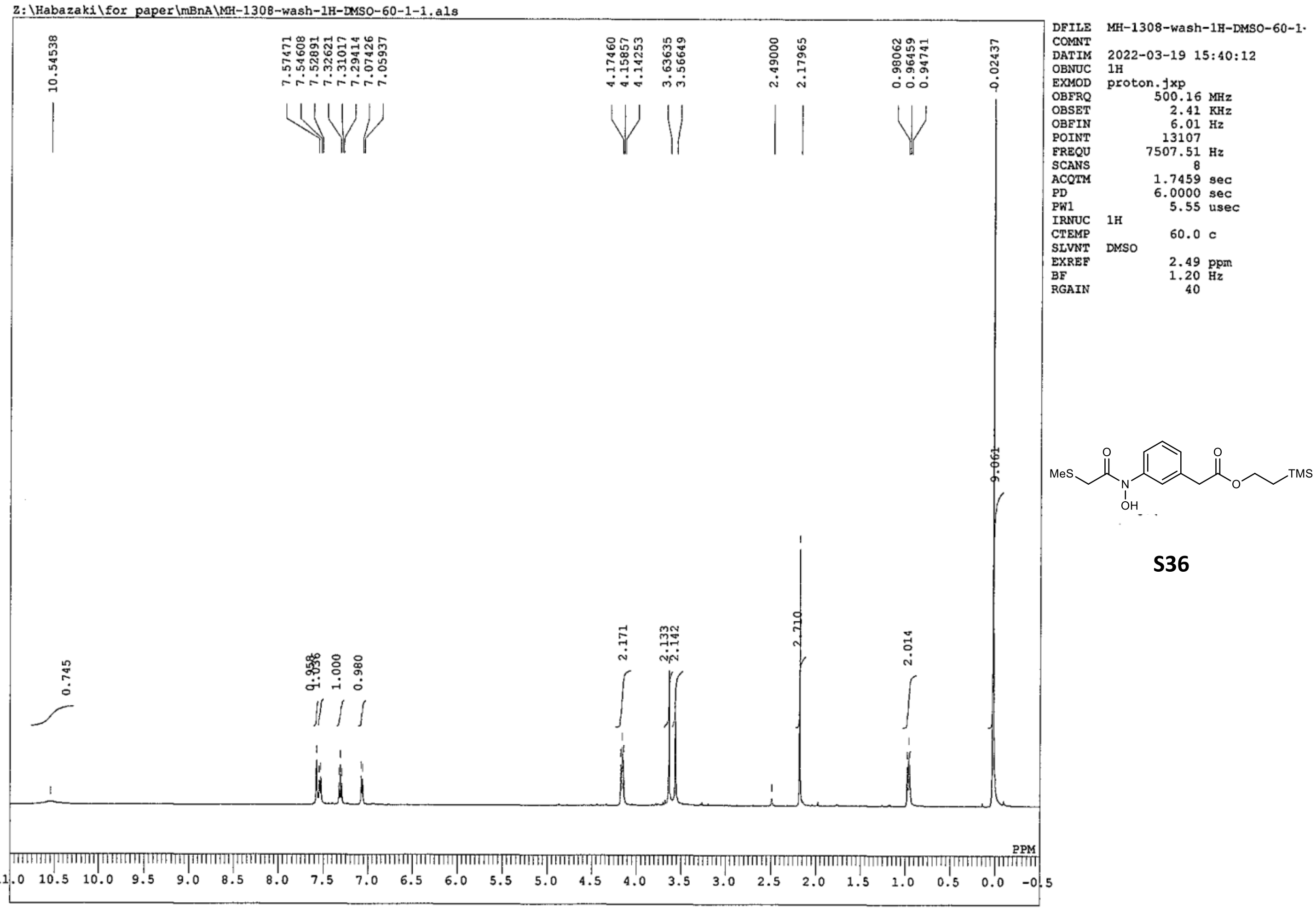

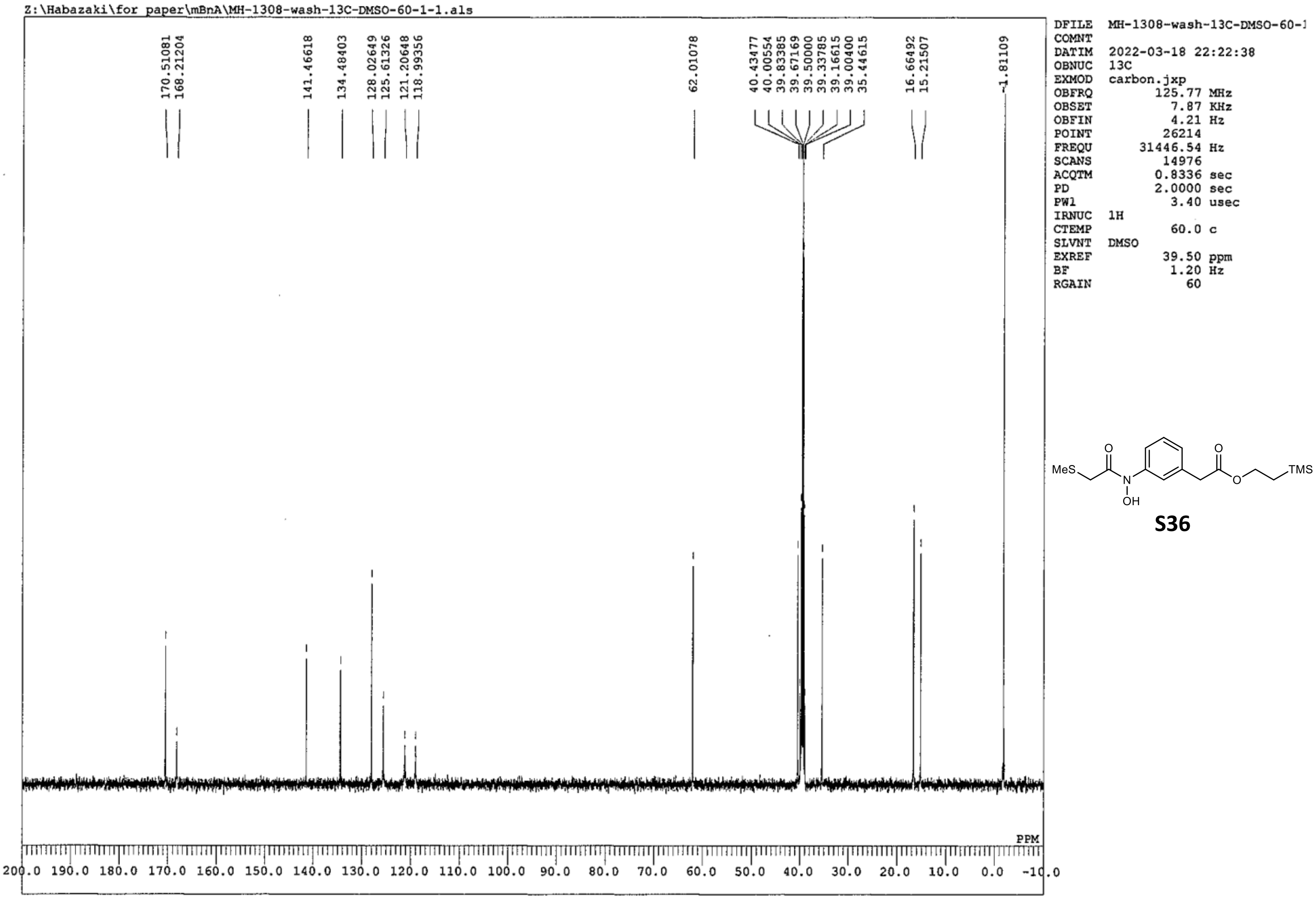

Supplementary Spectral Data 24. ^{1}H and ^{13}C NMR spectra of compound S37

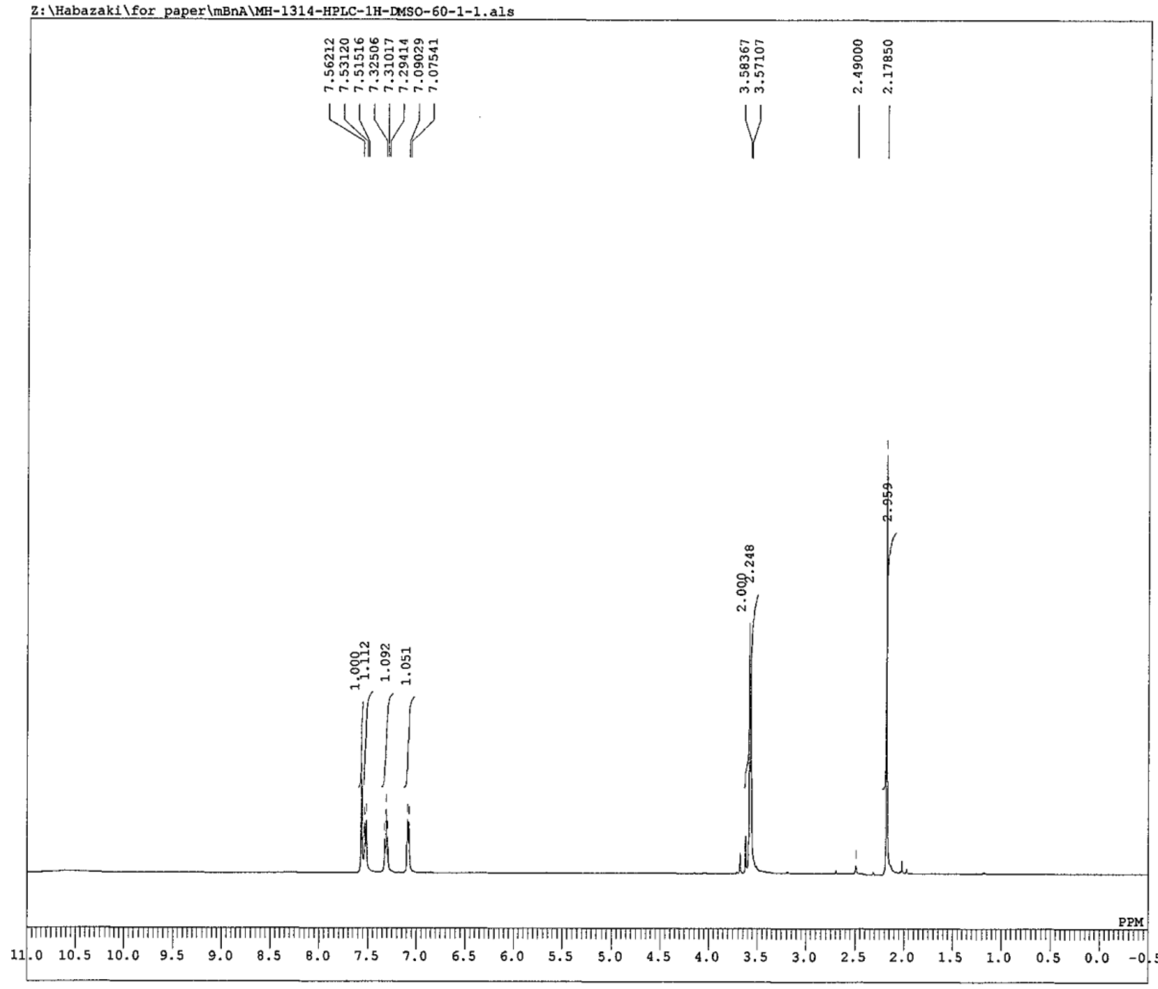

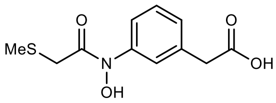

S37

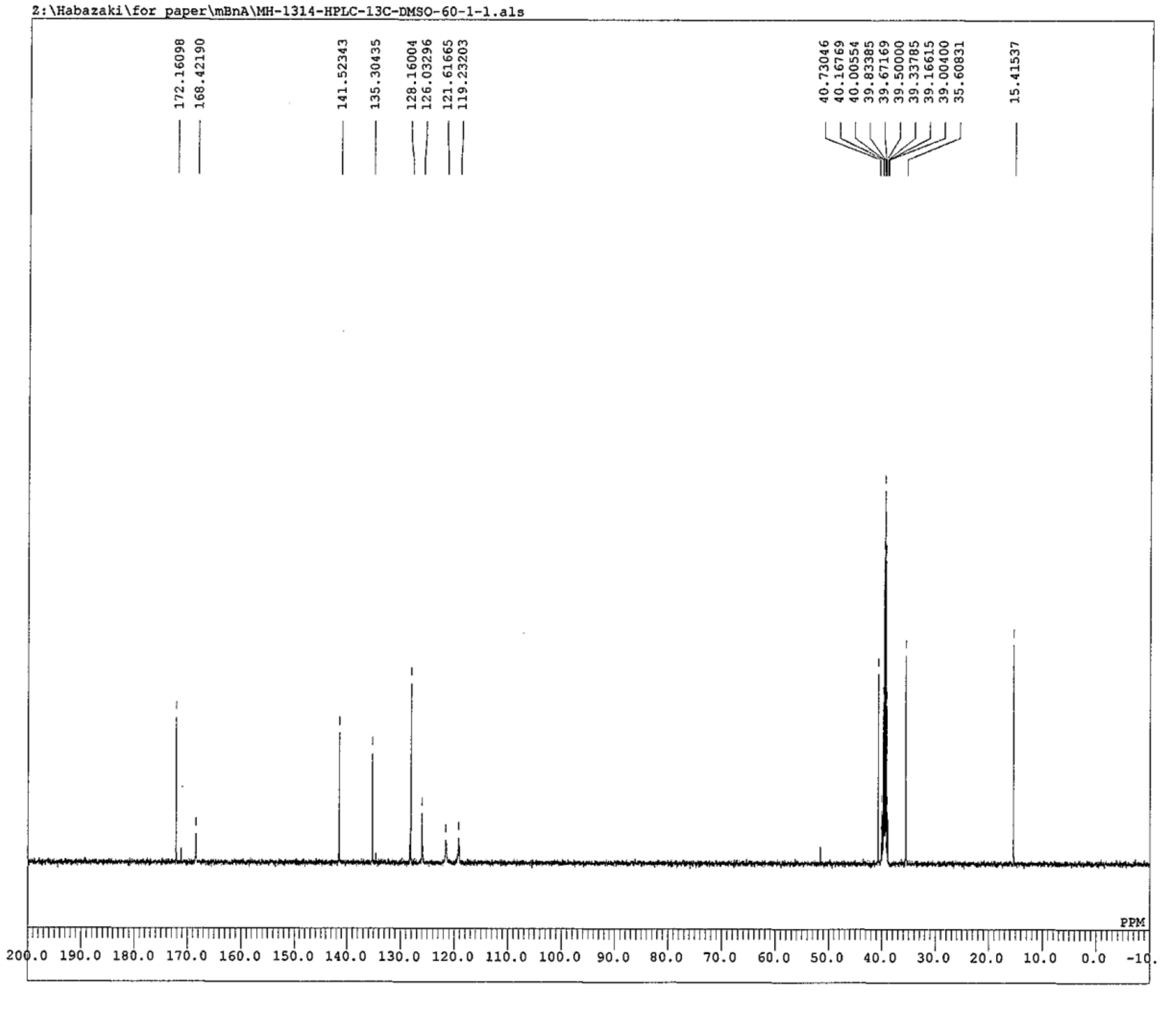

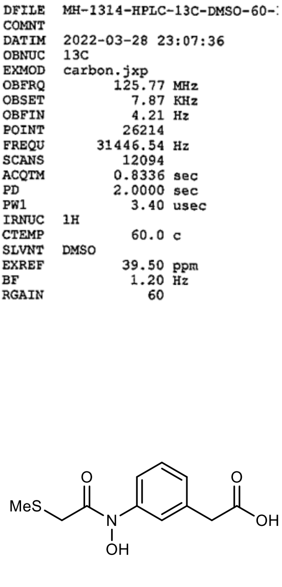

S37

Supplementary Spectral Data 25. ^{1}H and ^{13}C NMR spectra of compound 6

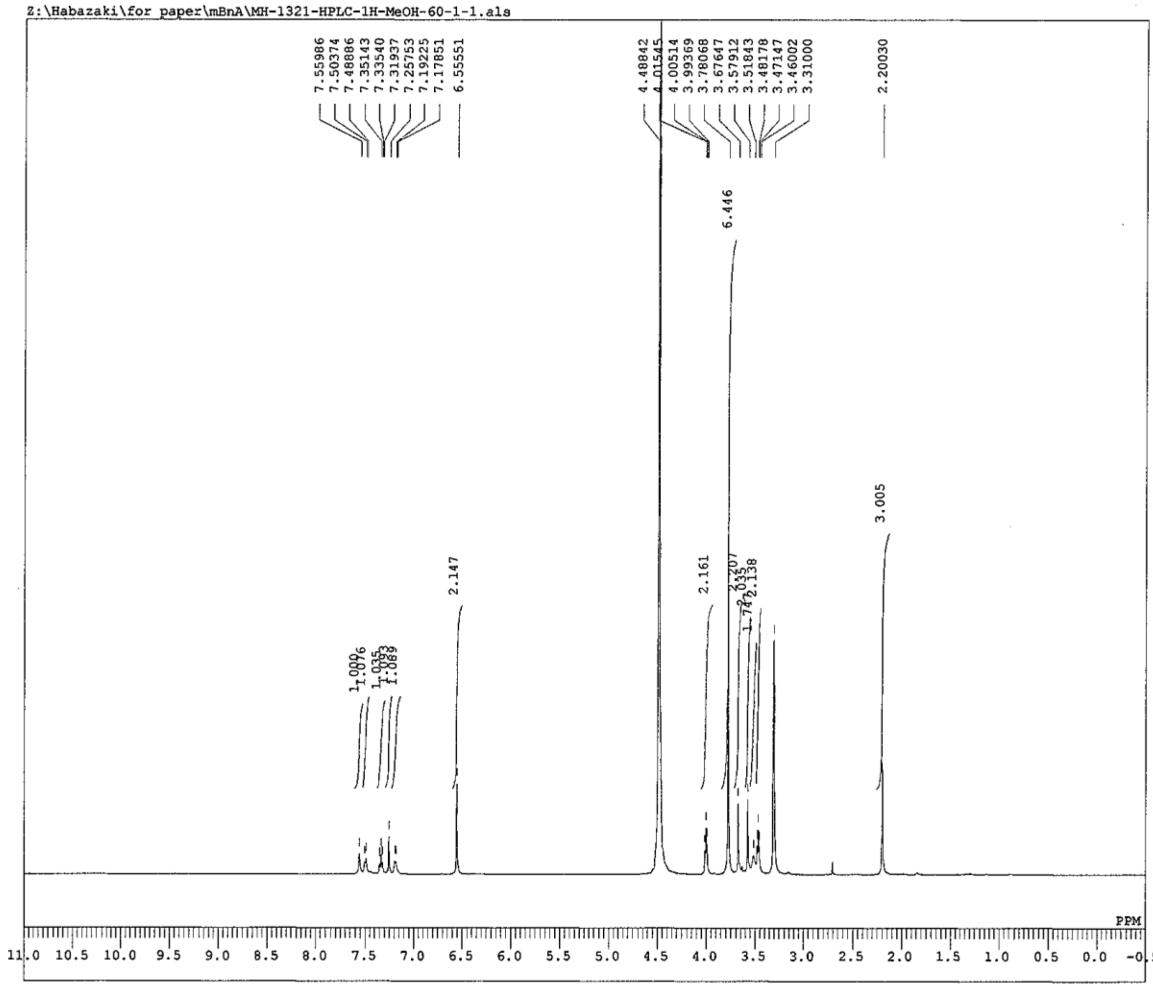

DFILE MH-1321-HPLC-1H-MeOH-60-1-
COMNT
DATIM 2022-03-30 15:36:39
OBNUC 1H
EXMOD proton.jxp
OBFRQ 500.16 MHz
OBSET 2.41 KHz
OBFIN 6.01 Hz
POINT 13107
FREQU 7507.51 Hz
SCANS 8
ACQTM 1.7459 sec
PD 6.0000 sec
PW1 5.55 usec
IRNUC 1H
CTEMP 60.0 c
SLVNT CD3OD
EXREF 3.31 ppm
BF 1.20 Hz
RGAIN 40

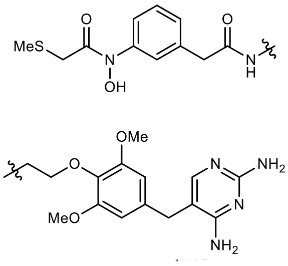

6

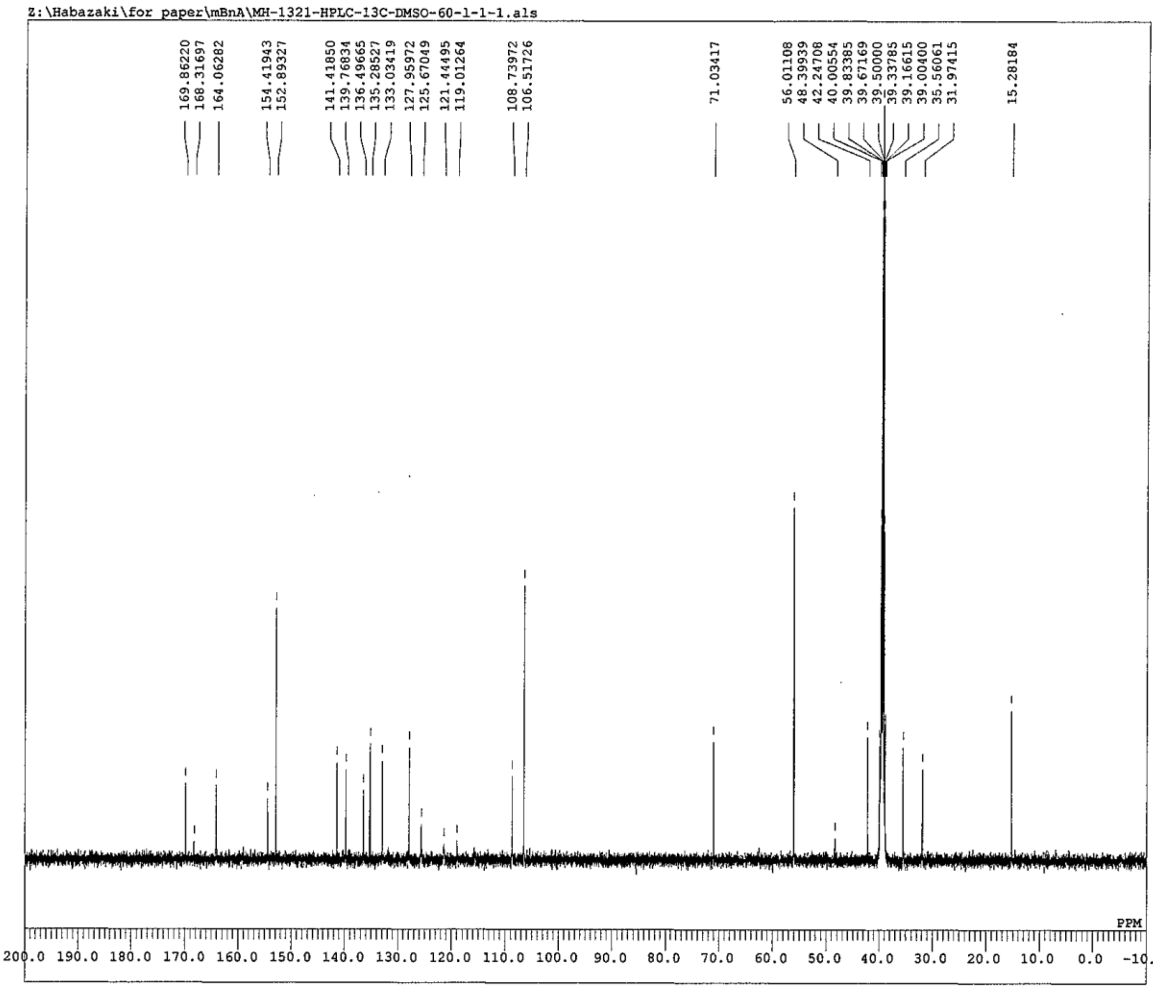

DFILE MH-1321-HPLC-13C-DMSO-60-
COMNT
DATIM 2022-04-09 22:23:38
OBNUC 13C
EXMOD carbon.jxp
OBFRQ 125.77 MHz
OBSET 7.87 KHz
OBFIN 4.21 Hz
POINT 26214
FREQU 31446.54 Hz
SCANS 24528
ACQTM 0.8336 sec
PD 2.0000 sec
PW1 3.40 usec
IRNUC 1H
CTEMP 60.0 c
SLVNT DMSO
EXREF 39.50 ppm
BF 0.62 Hz
RGAIN 60

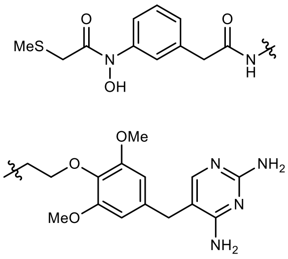

6

Supplementary Spectral Data 26. ^{1}H and ^{13}C NMR spectra of compound S40

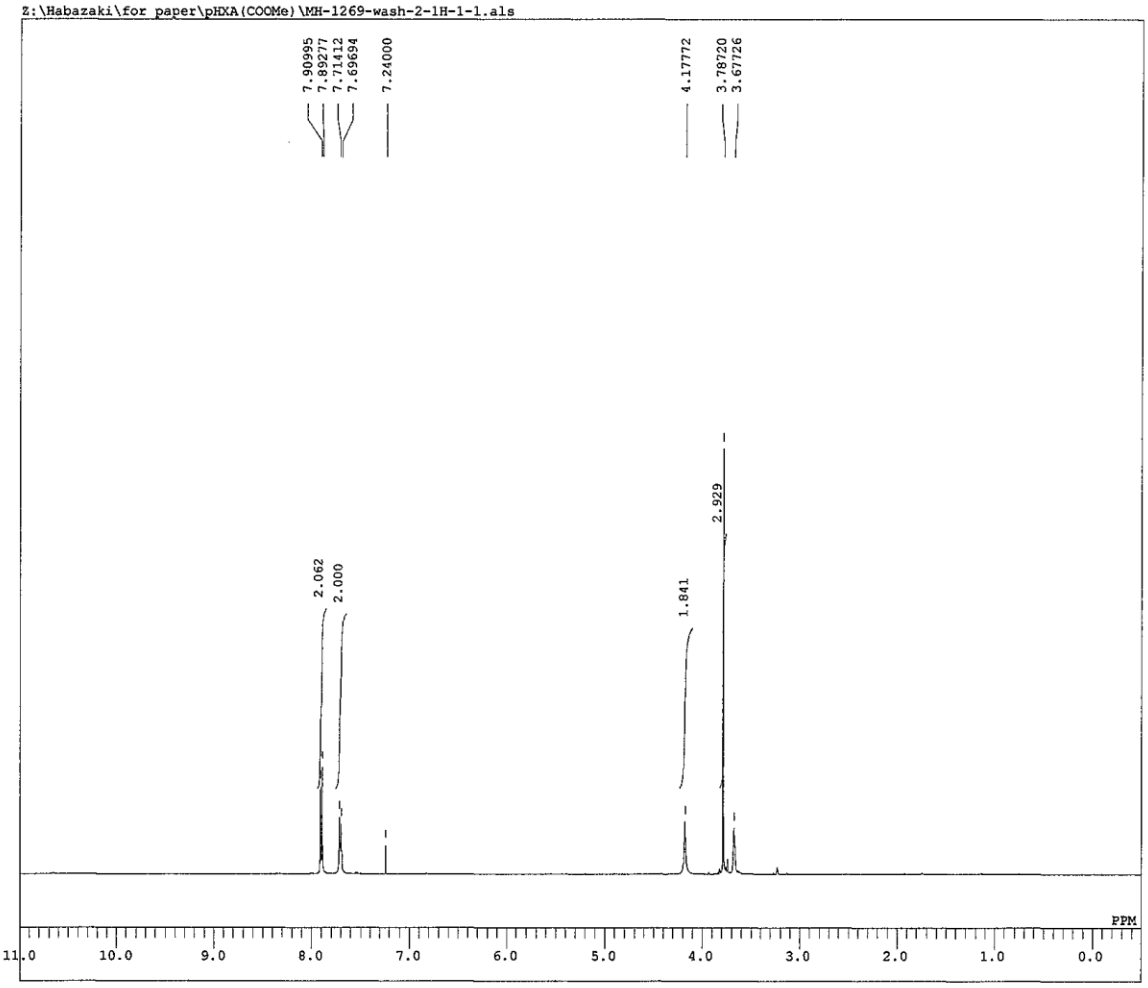

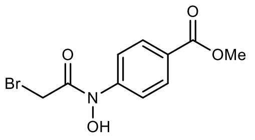

S40

* $CDCl_3/CD_3OD$ = 1/2

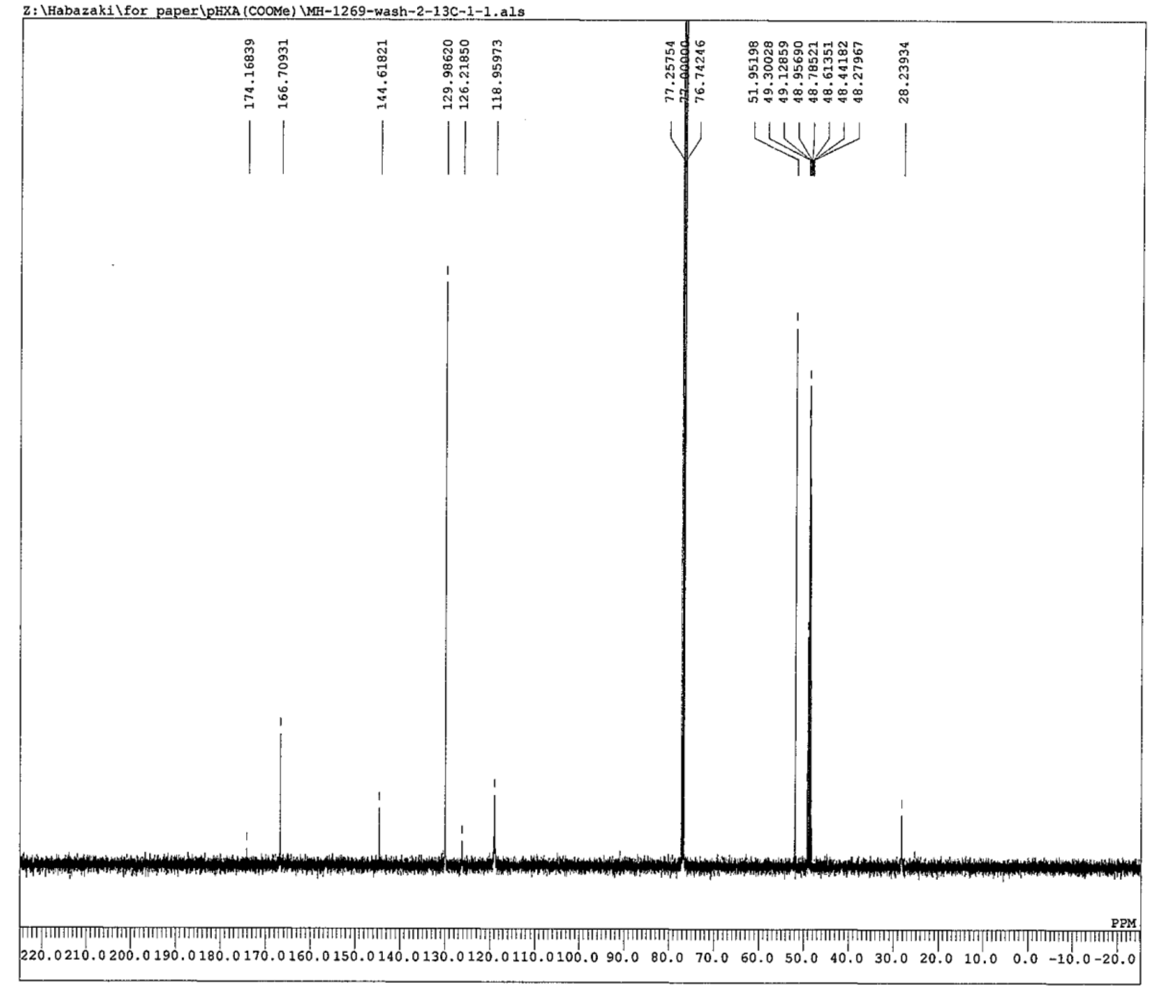

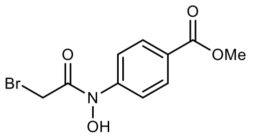

S40

* $CDCl_3/CD_3OD$ = 1/2

Supplementary Spectral Data 27. ^{1}H and ^{13}C NMR spectra of compound S41

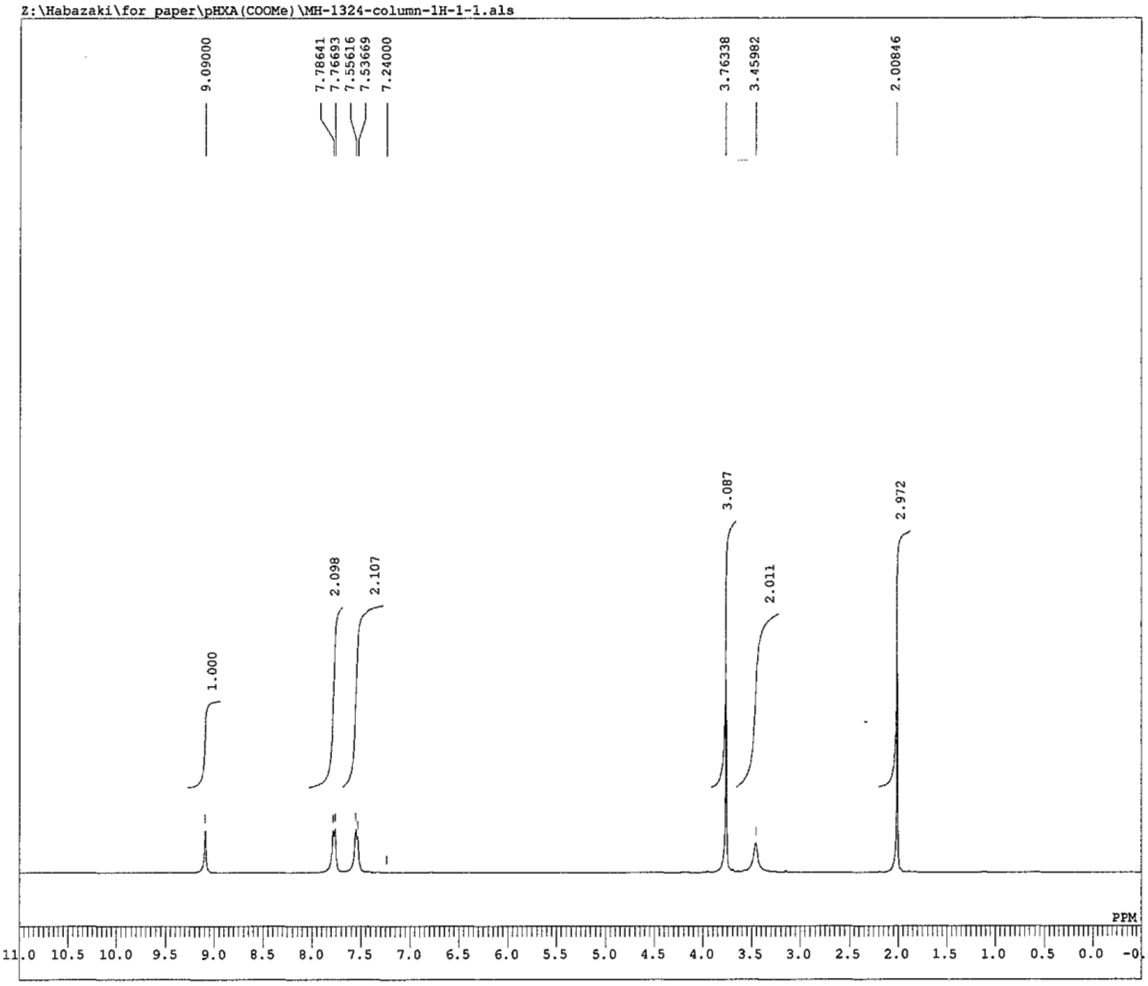

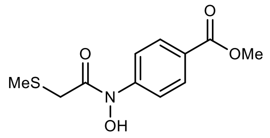

S41

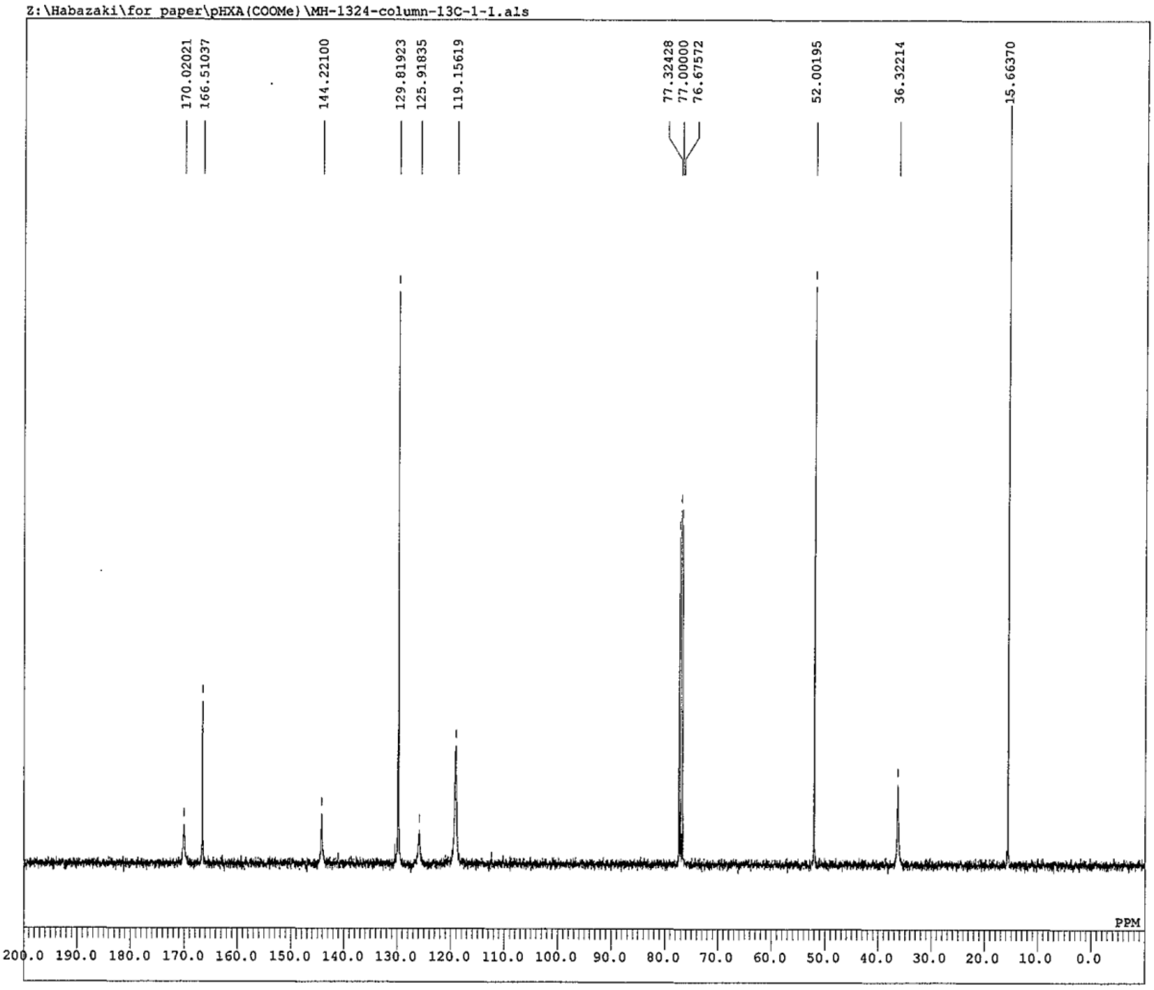

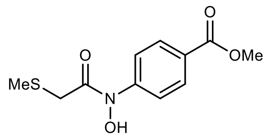

S41

Supplementary Spectral Data 28. ^{1}H and ^{13}C NMR spectra of compound S42

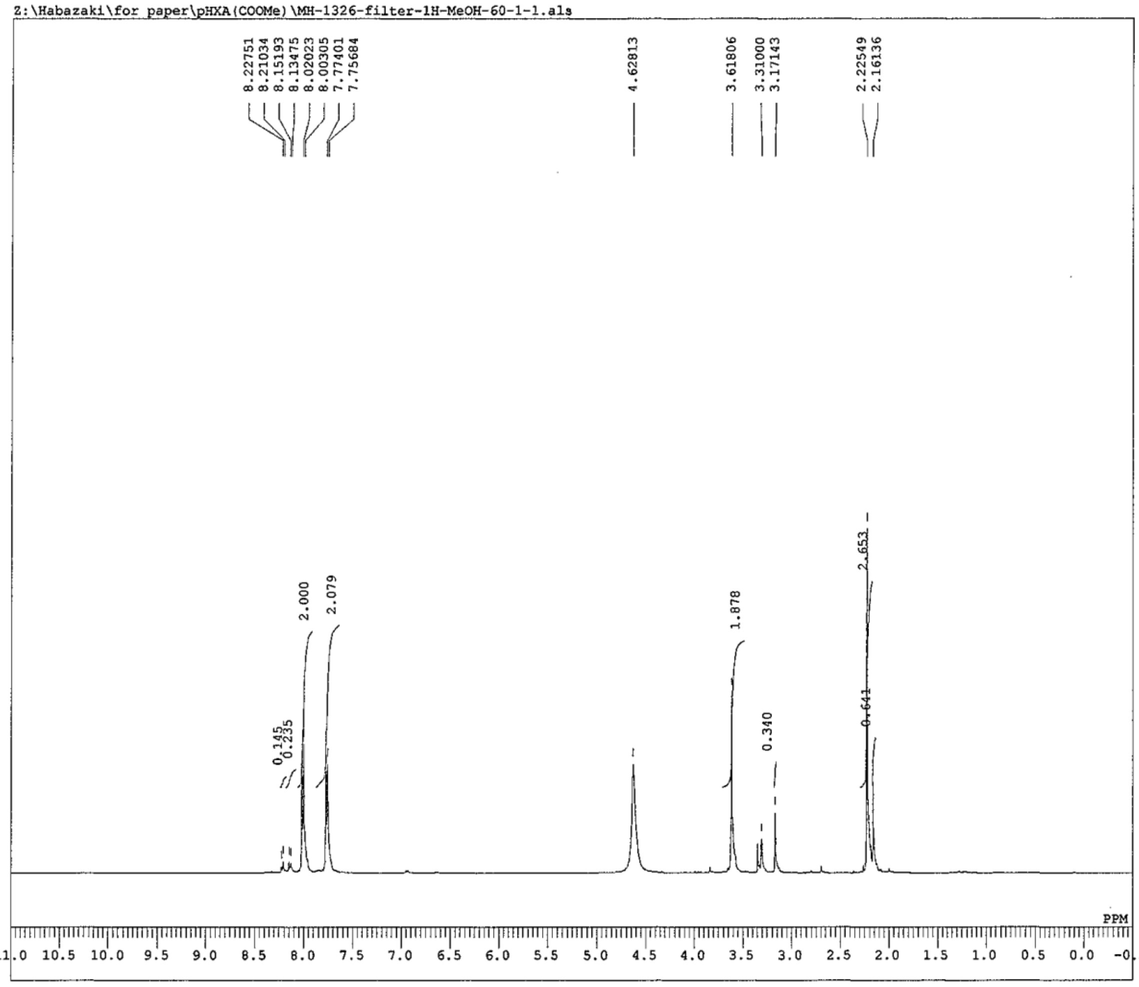

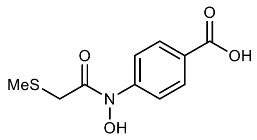

S42

* A ca. 10:1 mixture of *cis-trans* isomers at hydroxamic acid

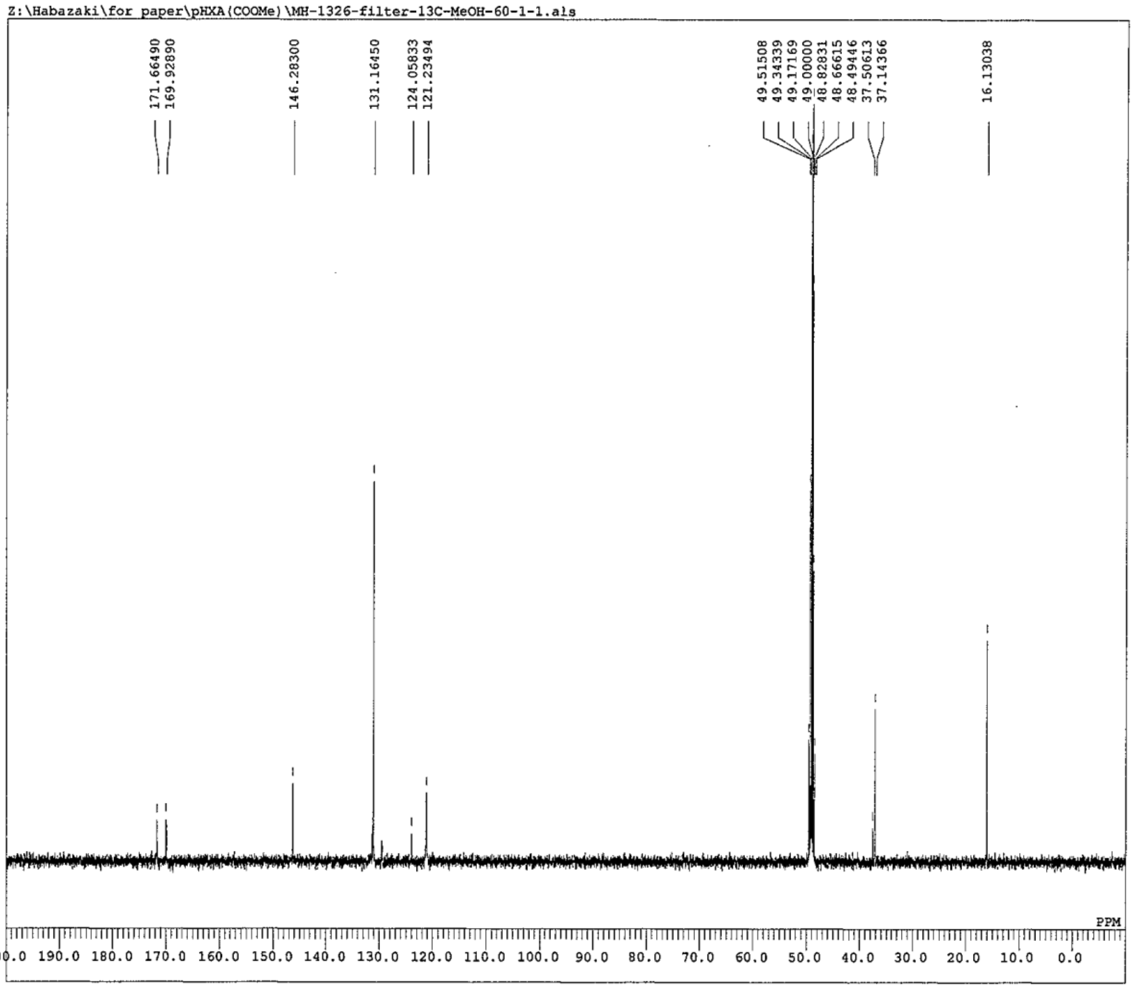

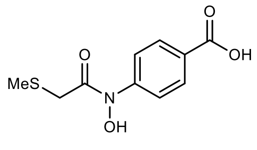

S42

* A ca. 10:1 mixture of *cis-trans* isomers at hydroxamic acid

Supplementary Spectral Data 29. ^{1}H and ^{13}C NMR spectra of compound S1

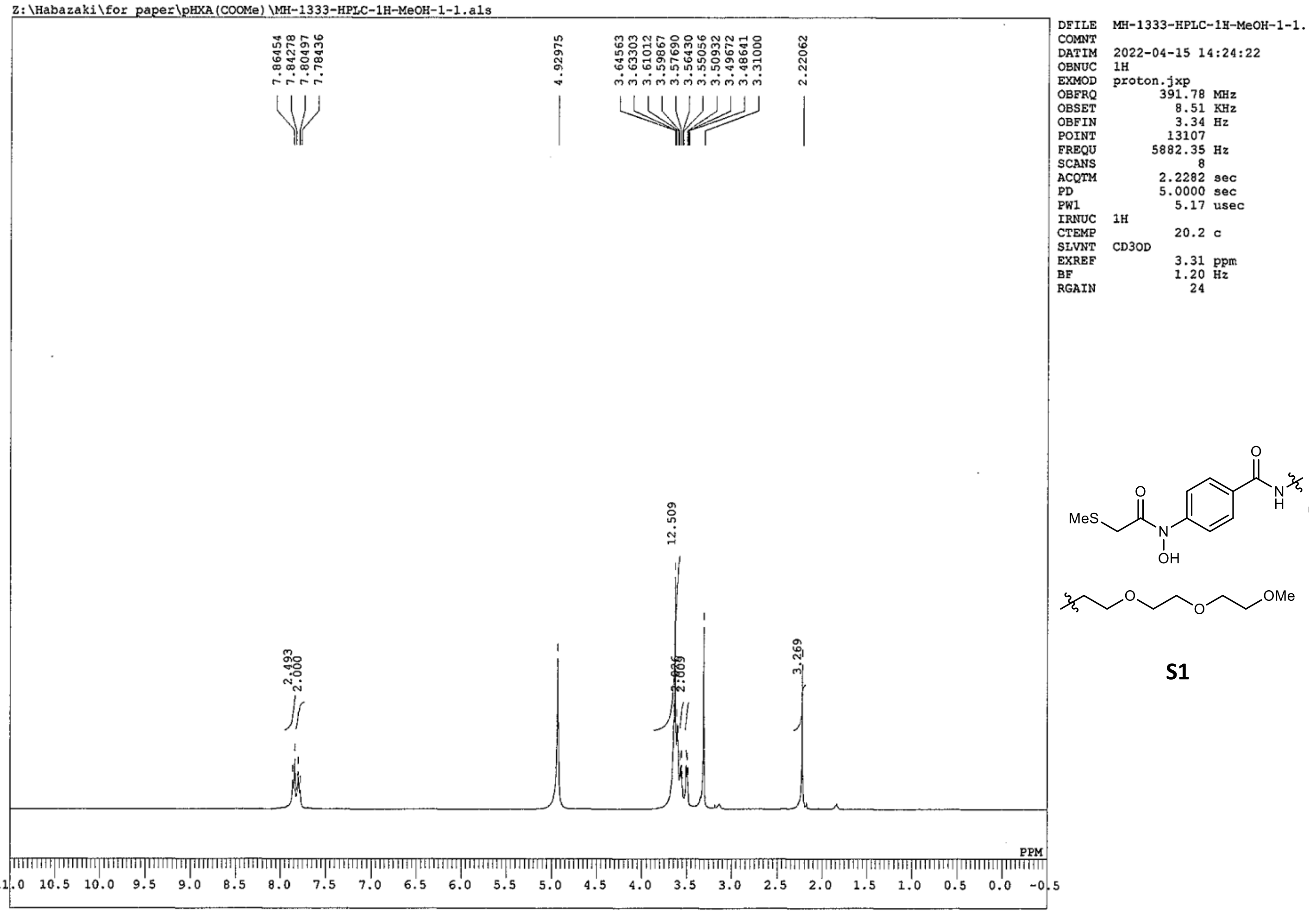

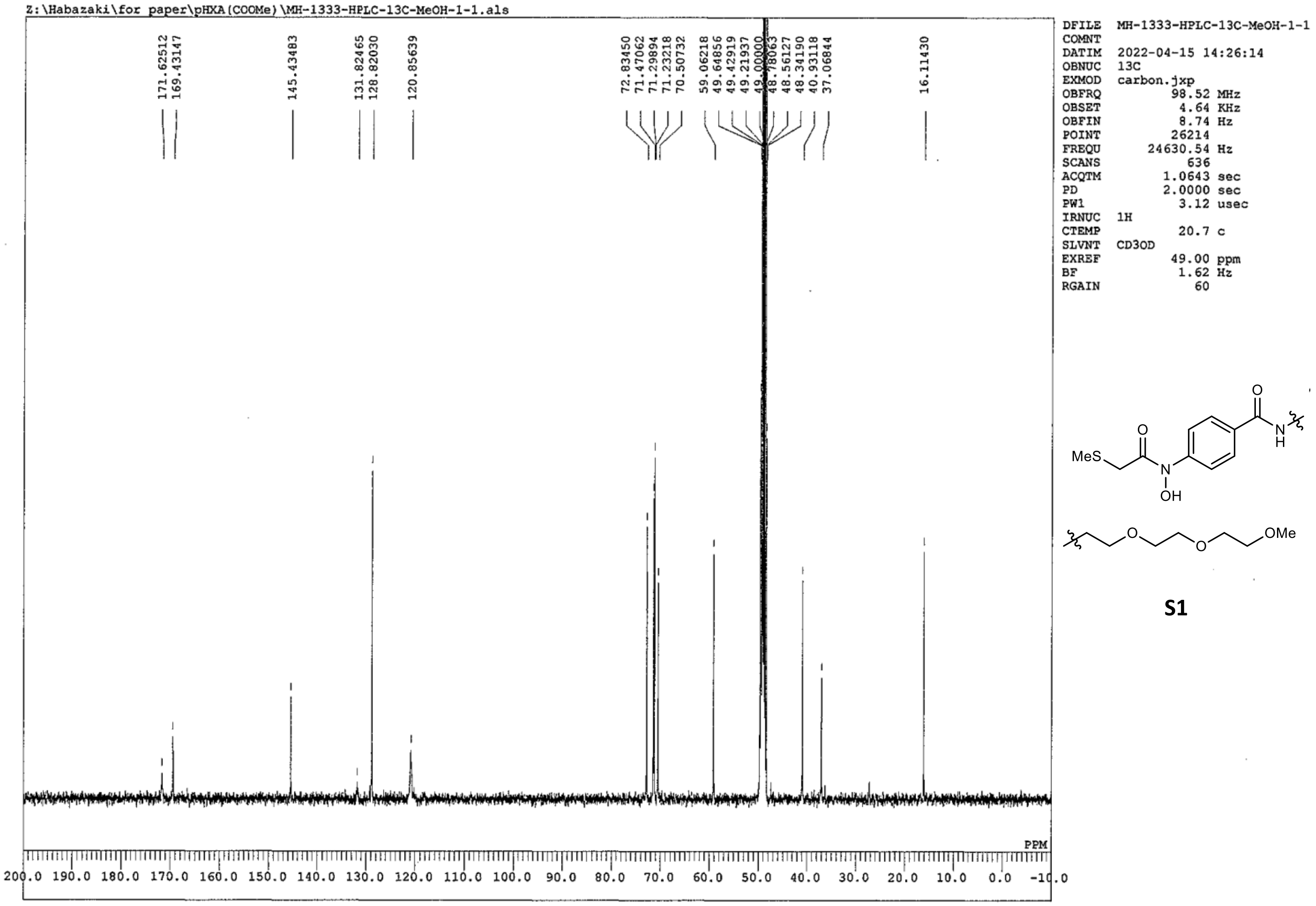

Supplementary Spectral Data 30. ^{1}H and ^{13}C NMR spectra of compound S2

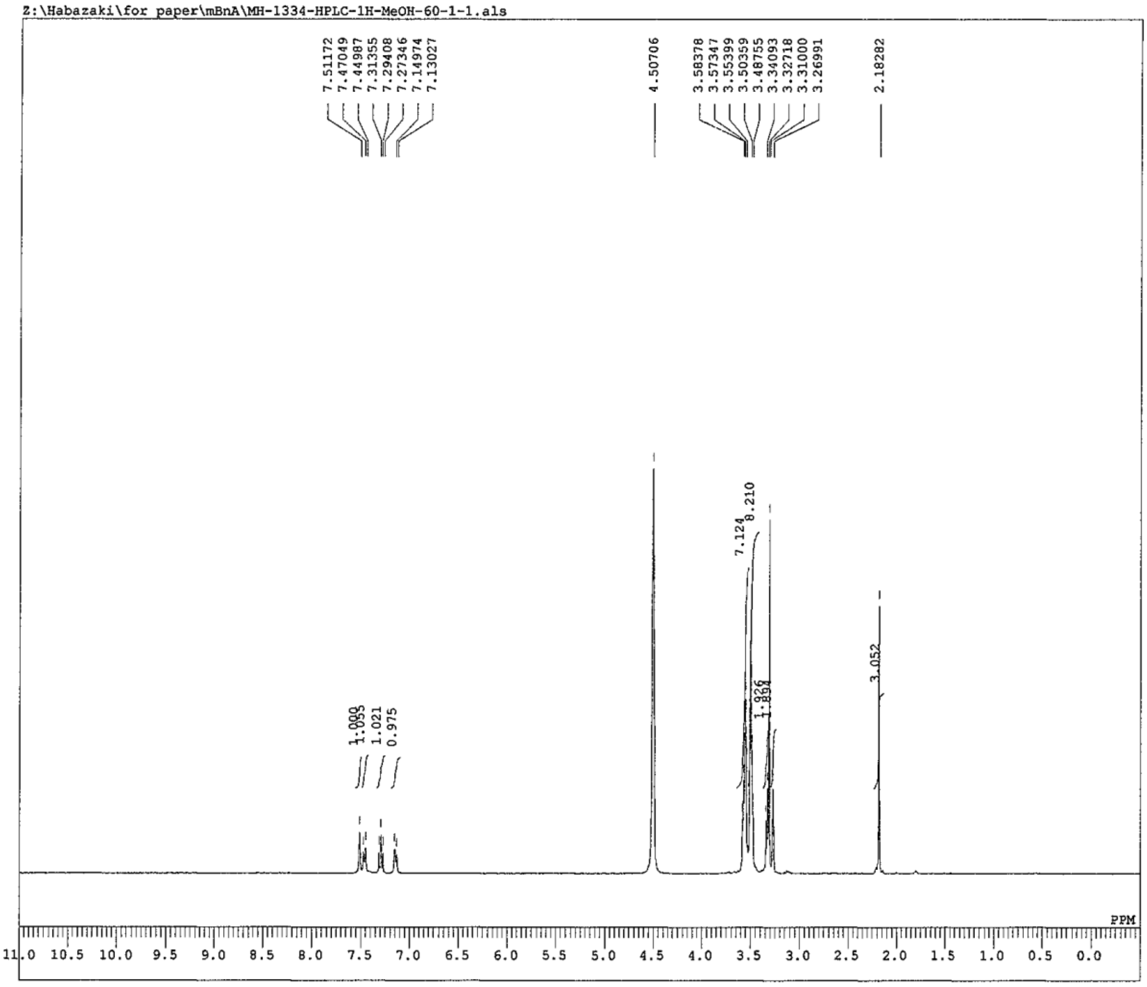


```
DFILE  MH-1334-HPLC-1H-MeOH-60-1-
COMNT
DATIM  2022-04-10 17:20:42
OBNUC  1H
EXMOD  proton.jxp
OBFRQ      391.78 MHz
OBSET        8.51 KHz
OBFIN        3.34 Hz
POINT       13107
FREQU     5882.35 Hz
SCANS           8
ACQTM      2.2282 sec
PD         5.0000 sec
PW1          5.17 usec
IRNUC  1H
CTEMP        60.0 c
SLVNT  CD3OD
EXREF        3.31 ppm
BF           0.62 Hz
RGAIN          38
```

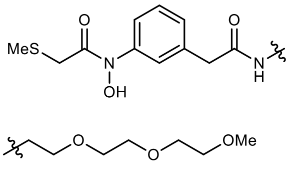

S2

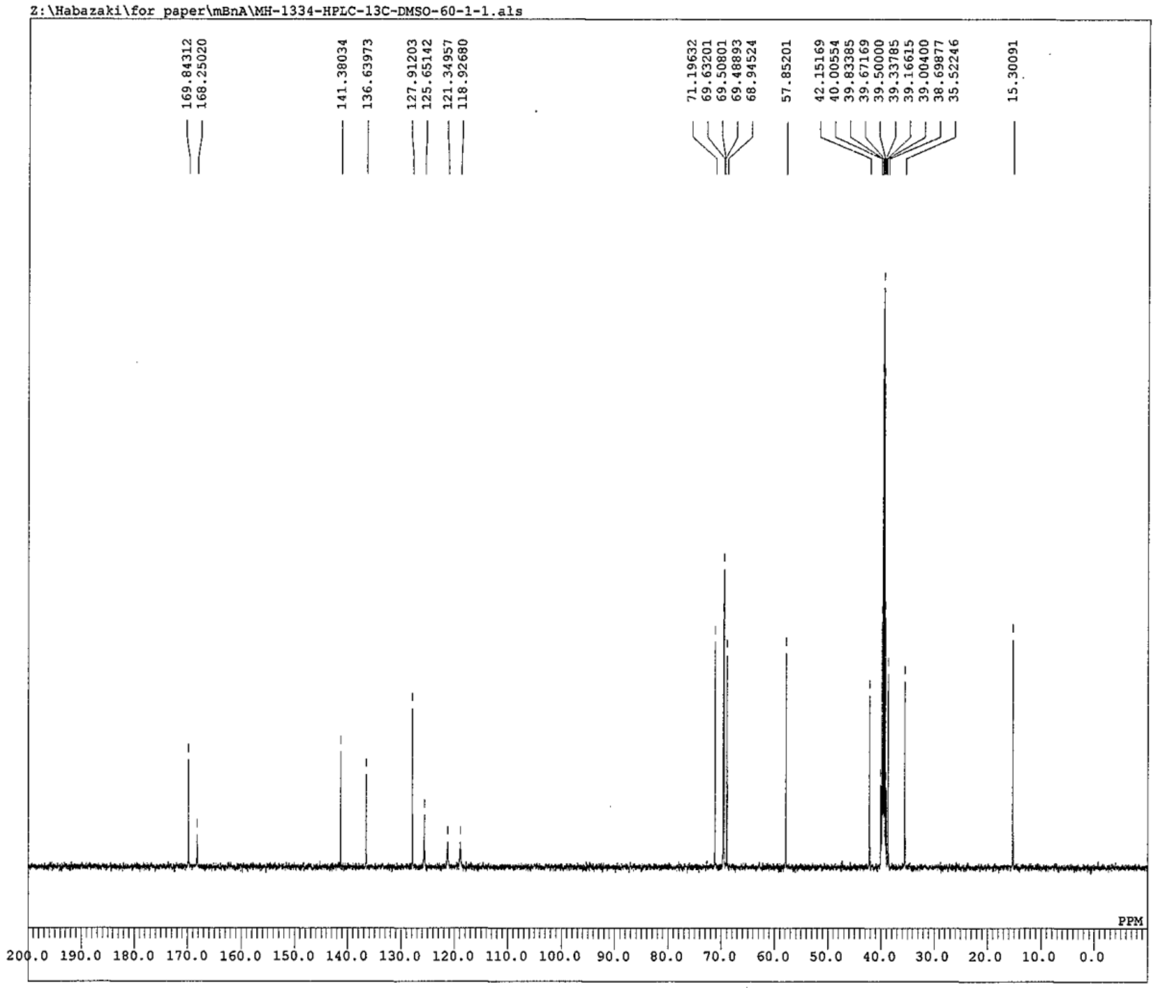


```
DFILE  MH-1334-HPLC-13C-DMSO-60-1
COMNT
DATIM  2022-04-10 17:47:35
OBNUC  13C
EXMOD  carbon.jxp
OBFRQ      125.77 MHz
OBSET        7.87 KHz
OBFIN        4.21 Hz
POINT       26214
FREQU    31446.54 Hz
SCANS       21363
ACQTM      0.8336 sec
PD         2.0000 sec
PW1          3.40 usec
IRNUC  1H
CTEMP        60.0 c
SLVNT  DMSO
EXREF       39.50 ppm
BF           1.62 Hz
RGAIN          60
```

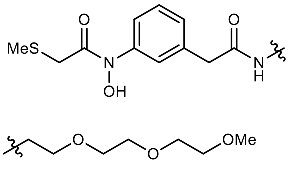

S2

Supplementary Spectral Data 31. ^{1}H and ^{13}C NMR spectra of compound S45

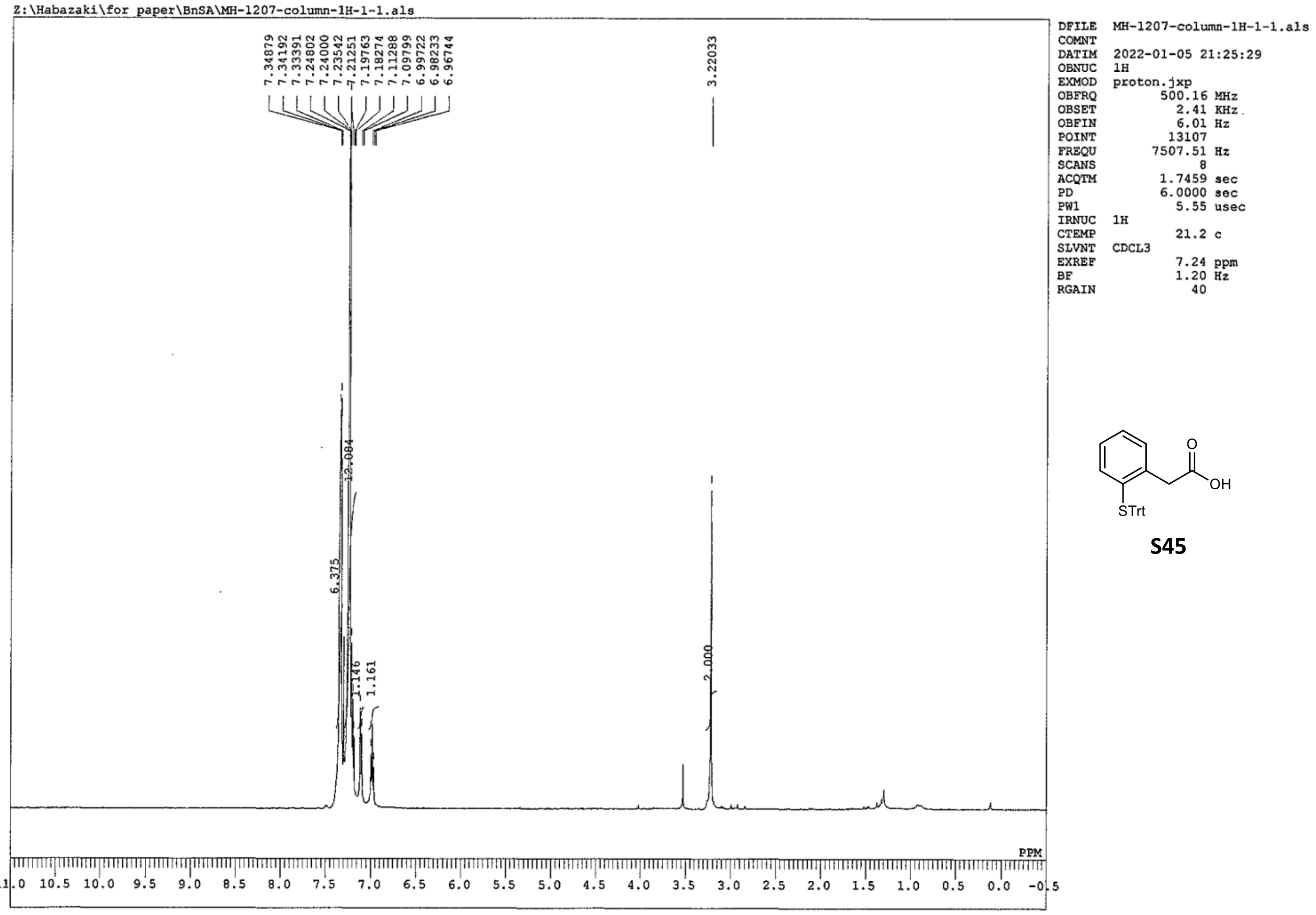

S45

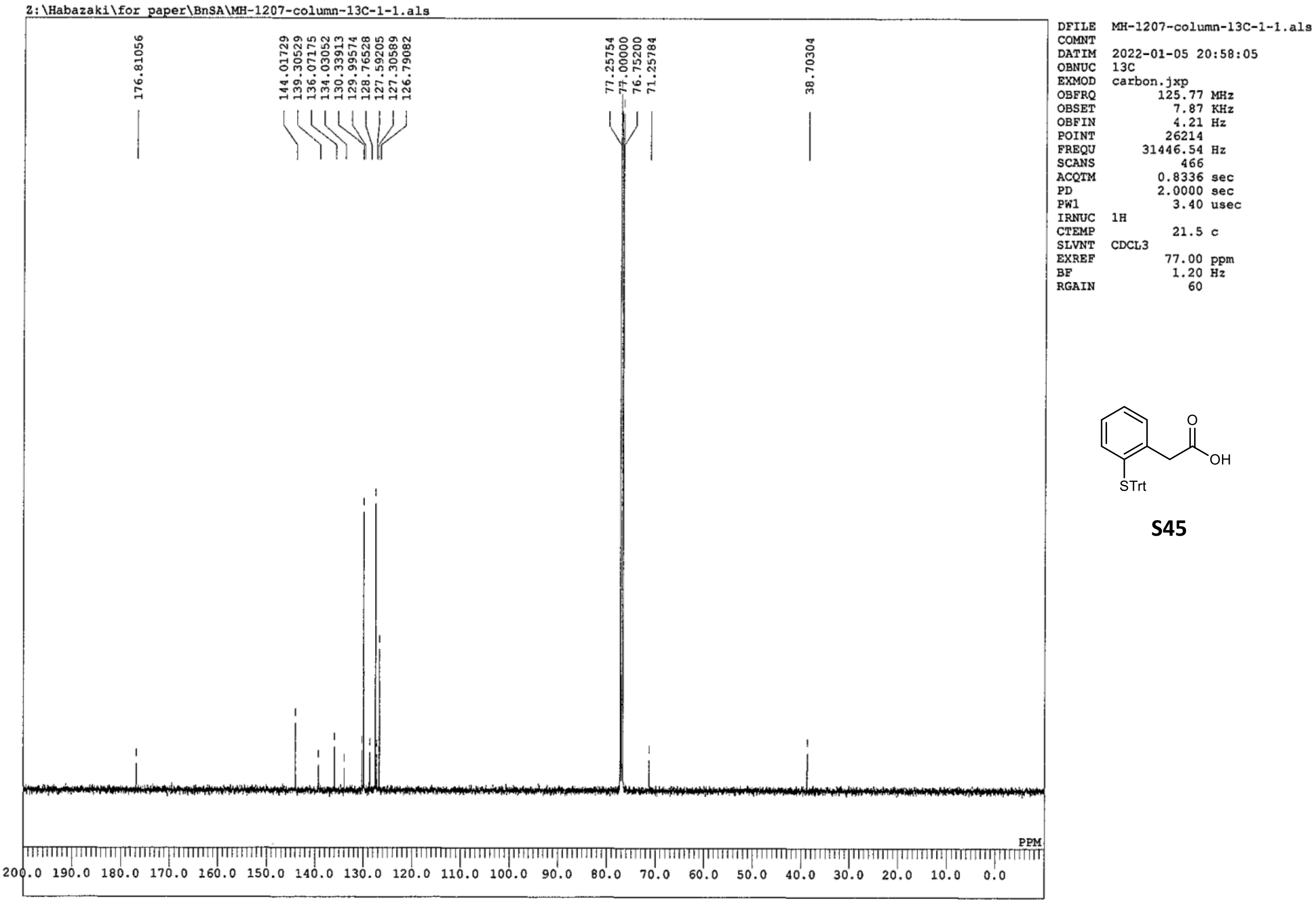

S45

Supplementary Spectral Data 32. ^{1}H and ^{13}C NMR spectra of compound S46

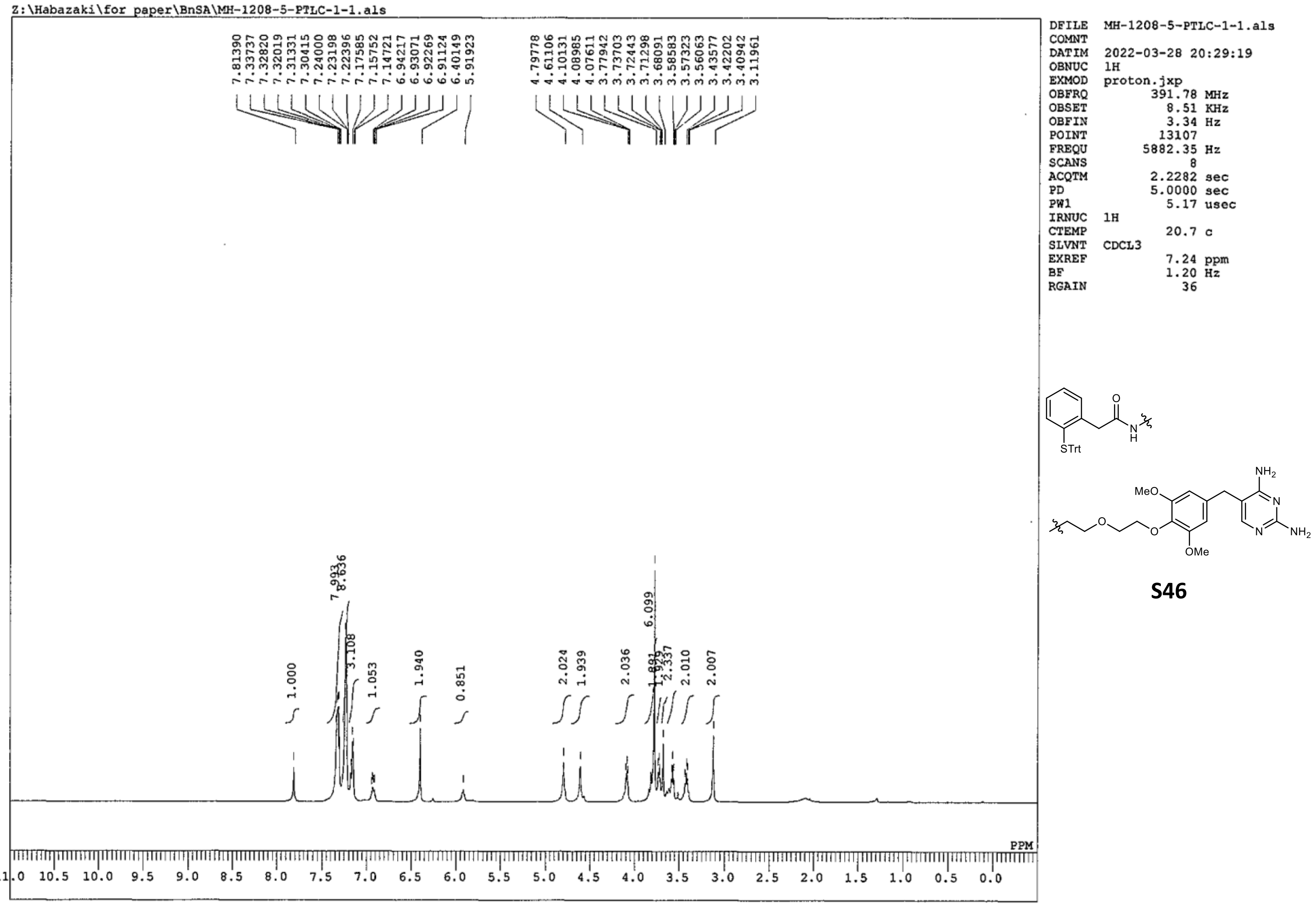

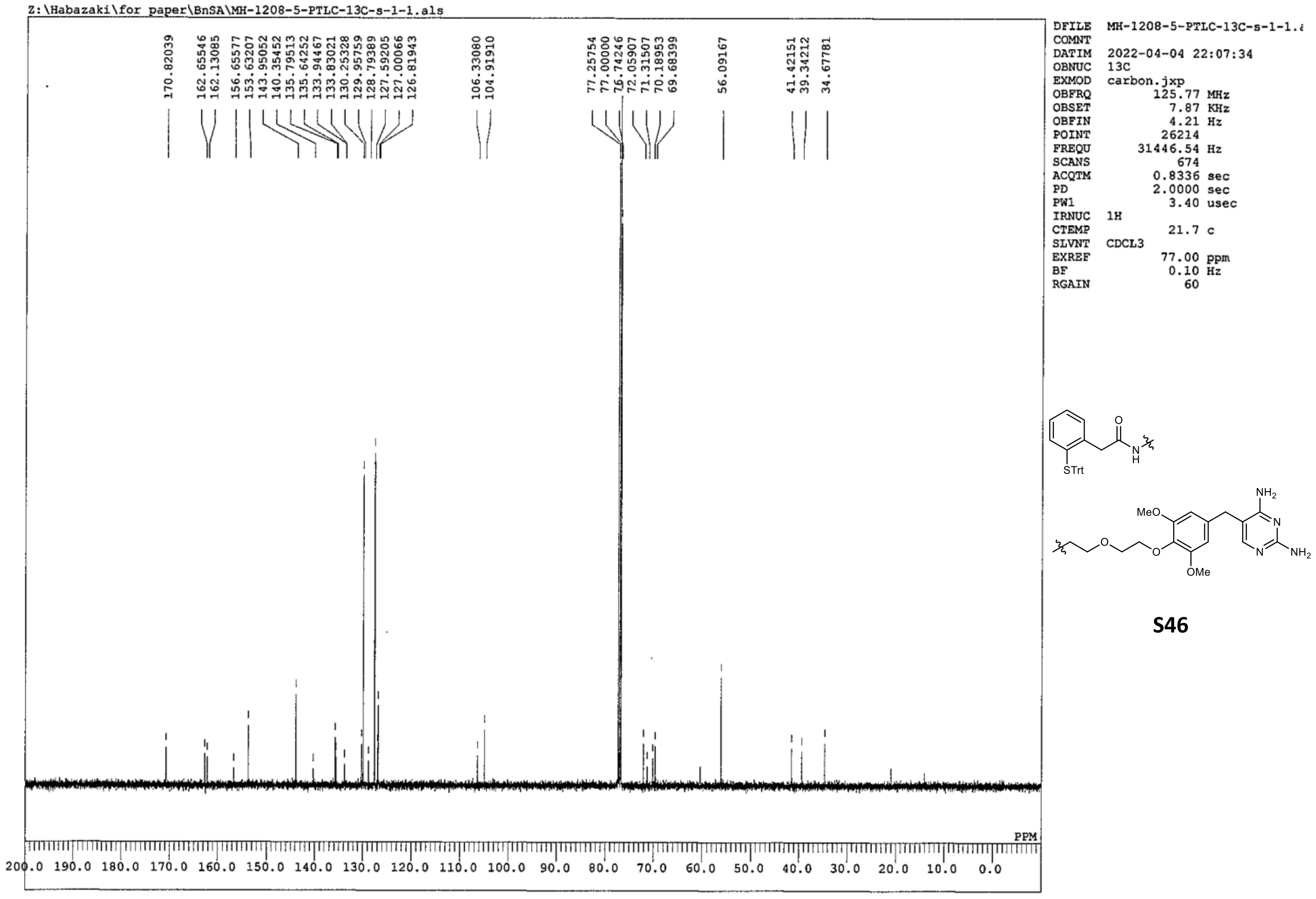

Supplementary Spectral Data 33. ^{1}H and ^{13}C NMR spectra of compound S5

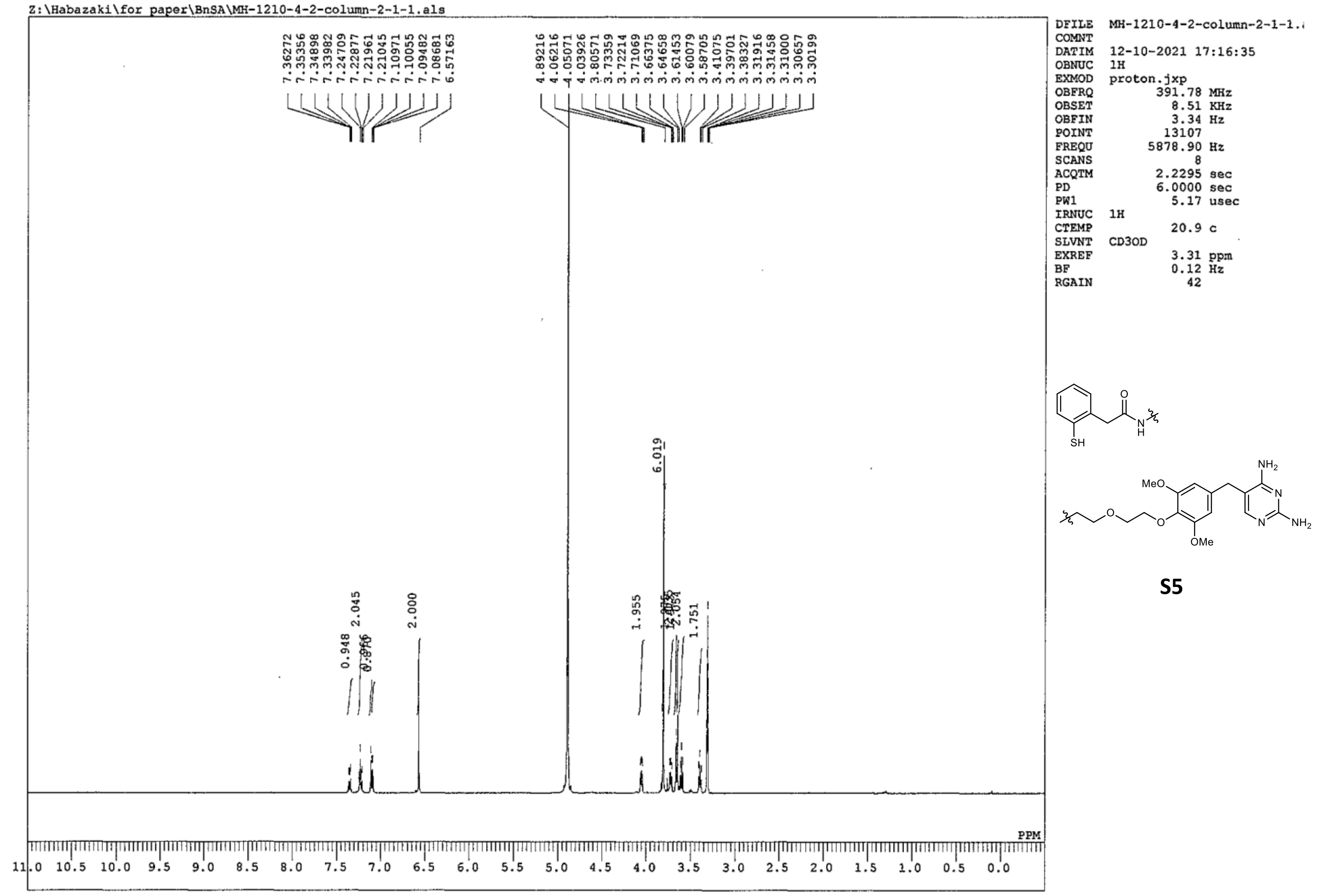

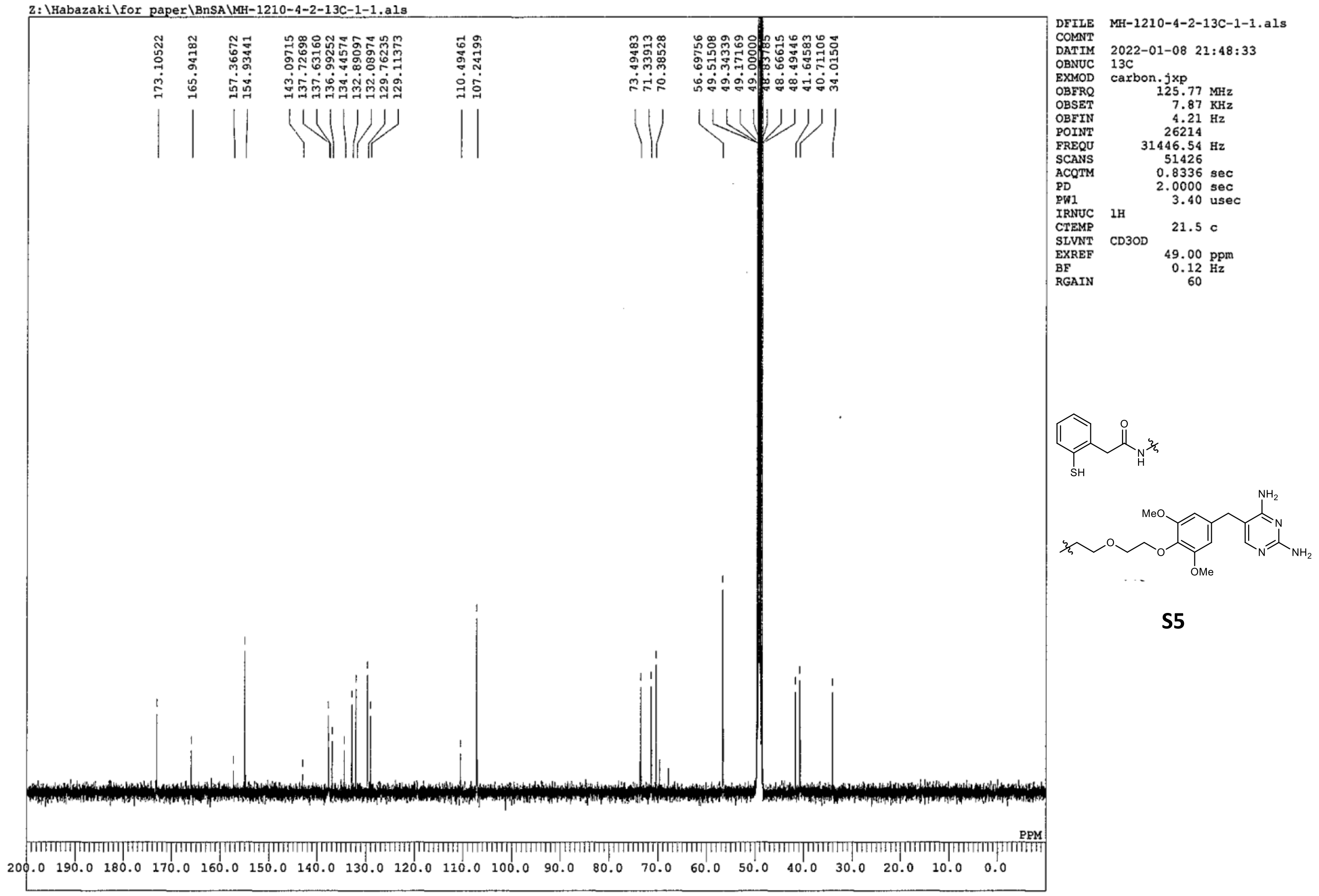

Supplement: Supplementary file 1 — Supplementary Information [file 41467_2023_41426_MOESM1_ESM.pdf]
